# Underlying Mechanism and Active Ingredients of Tianma Gouteng Acting on Cerebral Infarction as Determined *via* Network Pharmacology Analysis Combined With Experimental Validation

**DOI:** 10.3389/fphar.2021.760503

**Published:** 2021-11-16

**Authors:** Xiaolei Tang, Jing Lu, Haoyuan Chen, Lu Zhai, Yuxin Zhang, Huijuan Lou, Yufeng Wang, Liwei Sun, Bailin Song

**Affiliations:** ^1^ Research Center of Traditional Chinese Medicine, the Affiliated Hospital of Changchun University of Chinese Medicine, Changchun, China; ^2^ College of Traditional Chinese Medicine, Changchun University of Chinese Medicine, Changchun, China; ^3^ College of Pharmacy, Changchun University of Chinese Medicine, Changchun, China; ^4^ College of Acupuncture and Tuina, Changchun University of Chinese Medicine, Changchun, China; ^5^ Department of Tuina, the Affiliated Hospital to Changchun University of Chinese Medicine, Changchun, China; ^6^ Northeast Asian Research Institute of Traditional Chinese Medicine, Changchun University of Chinese Medicine, Changchun, China

**Keywords:** cerebral infarction, tianma gouteng, molecular docking, network pharmacology, KEGG pathway

## Abstract

Cerebral infarction (CI), a common cerebrovascular disease worldwide, is caused by unknown factors common to many diseases, including hypokalemia, respiratory diseases, and lower extremity venous thrombosis. Tianma Gouteng (TMGT), a traditional Chinese Medicine (TCM) prescription, has been used for the clinical treatment of CI. In this study, high-performance liquid chromatography (HPLC) fingerprint analysis was used to detect and identify major chemical constituents of TMGT. TCMSP and BATMAN-TCM databases were used to screen for active TMGT constituent compounds, while the GeneCards database was used to screen for protein targets associated with CI. Next, GO and KEGG enrichment analysis of these core nodes were performed to determine the identities of key associated biological processes and signal pathways. Meanwhile, a total of six possible gene targets of TMGT, including NFKBIA, PPARG, IL6, IL1B, CXCL8, and HIF1A, were selected for further study using two cellular models of CI. For one model, PC12 cells were treated under oxygen and glucose deprivation (OGD) conditions to generate an OGD cellular model of CI, while for the other model, BV2 cells were stimulated with lipopolysaccharide (LPS) to generate a cellular model of CI-associated inflammation. Ultimately TMGT treatment increased PPARγ expression and downregulated the expression of p-P65, p-IκBα, and HIF-1α in both OGD-induced and LPS-induced cell models of CI. In addition, molecular docking analysis showed that one TMGT chemical constituent, quercetin, may be a bioactive TMGT compound with activity that may be associated with the alleviation of neuronal damage and neuroinflammation triggered by CI. Moreover, additional data obtained in this work revealed that TMGT could inhibit neuroinflammation and protect brain cells from OGD-induced and LPS-induced damage by altering HIF-1α/PPARγ/NF-κB pathway functions. Thus, targeting this pathway through TMGT administration to CI patients may be a strategy for alleviating nerve injury and neuroinflammation triggered by CI.

## Introduction

Stroke, a common cerebrovascular disease worldwide with high morbidity and mortality, can seriously impact an individual’s quality of life (Takeda et al., 2021). Results of statistical analysis of current trends indicate that the incidence of ischemic stroke will remain high in the coming decades (Howard and Goff, 2012). Cerebral infarction (CI) is a state of stroke accompanied by the obstruction of blood flow that leads to the interruption of blood supply to CNS tissues. Various biological processes are involved in the development or progression of CI, including neuroinflammation and ischemia-hypoxic injury. Clinically, multiple interventions are used to treat stroke due to the complex pathogenic mechanisms associated with the disorder. However, patient outcomes are often unsatisfactory, prompting researchers to find new drugs or treatment strategies to ameliorate CNS damage resulting from CI.

The biological process of neuroinflammation, which often occurs after brain ischemic injury, can often promote accelerated progression of CI (Xu et al., 2018). Previous studies had revealed that activated B cell nuclear factor kappa light chain enhancer (NF-κB) was closely associated with the development of CI-induced neuroinflammation (Gogoleva et al., 2019; Hammond et al., 2019). Interestingly, components of NF-κB, p65, and p50 have been shown to be phosphorylated under conditions of CI-induced inflammation (Famakin and Vemuganti, 2020). In this scenario, activated IκBα was phosphorylated and degraded by IκB kinases (IKKs) in resting cells and then induced the activation of p65/p50NF-κB, thereby promoting the transcription of many pro-inflammatory cytokine genes (Li X. et al., 2020), implying that blocking the activated NF-κB signal might be an effective strategy for inhibiting CI-induced neuroinflammation. Also, previous studies had reported that the increased angiogenesis could provide enough oxygen and nutrients to support greater survival of neurons after injury, thereby reducing CI-induced CNS damage and restoring nerve function (Liu et al., 2014). Meanwhile, angiogenesis and nerve function recovery after CI have been shown to be closely linked to PPARγ (peroxisome proliferator-activated receptor γ) expression. Notably, PPARγ, a key nuclear transcription factor, was shown to play a role in neuroprotection after CNS injury was reported to be associated with neuroinflammation (Li J. et al., 2020). In an *in vitro* animal study, PPARγ was identified as a therapeutic target due to its observed ability to induce microglial activation and increase the expression of inflammatory factors in MCAO rats (Ye et al., 2021).

Traditional Chinese Medicine (TCM) has become a research hot spot in recent years due to its many advantages as a treatment for CI (Das et al., 2020). Notably, one TCM formulation, TMGT, has been widely used to treat brain-related diseases, including CI. TMGT contains two components, gastrodiae and uncaria (Table 1). Gastrodia has been commonly used clinically to treat CI due to its effects on different biological processes. For example, gastrodia may promote microvascular regeneration and prevent neuronal apoptosis through the upregulation of the expression of anti-apoptotic proteins such as Bcl-2, while suppressing Bax and caspase 3 expression and increasing VEGF levels. In MCAO mice, gastrodia treatment restrained the progression of the CI-associated inflammatory response by reducing CRP and IL-1β expression (Yu et al., 2010; Shen et al., 2015; Peng et al., 2018). In addition, uncaria, which is another therapeutic drug used to treat nerve-related diseases, was shown to prevent cerebral ischemic damage by maintaining blood–brain barrier integrity through the inhibition of tight junction degradation and MMP-9 activity (Seo et al., 2015). Moreover, uncaria has been shown to prevent ischemic injury by activating the PI3K/AKT/mTOR signal pathway and inhibiting the TLR/NF-κB pathway (Huang et al., 2014). Nevertheless, although Tianma and Gouteng have been used widely for the clinical treatment of CI, the molecular mechanisms underlying their beneficial effects are unknown.

**TABLE 1:** The components of the Tianma Gouteng (TMGT) formula.

Chinese name	Latin name in Chinese pharmacopoeia (Ch.P.)	Family	Weight (g)	Plant part (s), processing	Vucher specimen
Tian ma	Gastrodiae Rhizoma	Orchidaceae	10	Root	201213-1
Gou teng	Uncariae Ramulus Cum Uncis	Rubiaceae	10	Hooked stems	201213-2

Network pharmacology analysis is a relatively new bioinformatics method that can be used to elucidate synergistic effects and potential mechanisms of action of multicompound and multitarget drugs through the analysis of various complex interaction networks (Wang et al., 2013). In this work, network pharmacology analysis was employed to reveal possible activities and molecular mechanisms of action of TMGT for alleviating CNS damage induced by CI.

## Materials and Methods

### Preparation of TMGT and High-Performance Liquid Chromatography Analysis

**FIGURE 1:**
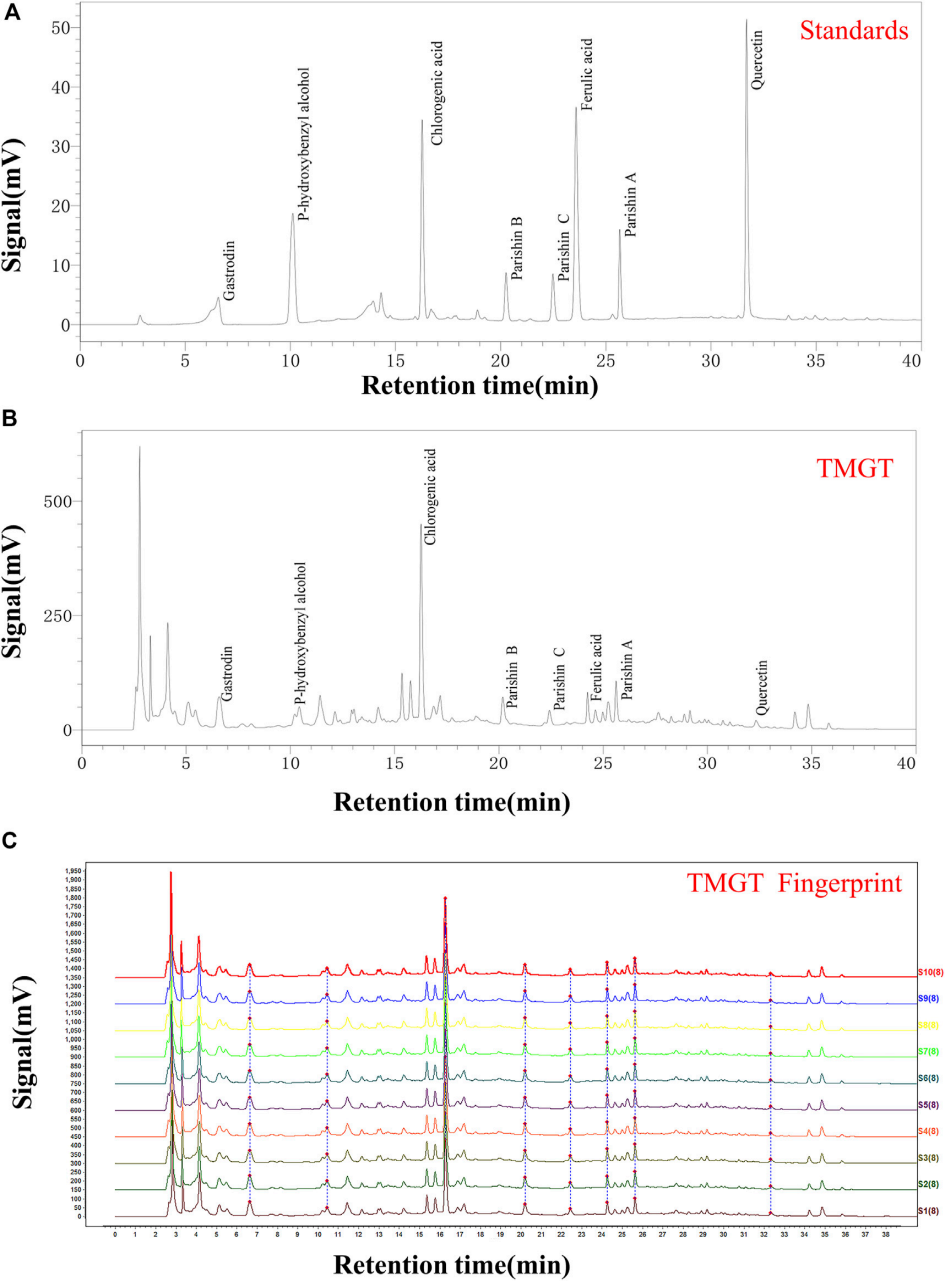
HPLC chromatogram of TMGT. **(A)** HPLC chromatograms of mixed standards including gastrin, *p*-hydroxybenzyl alcohol, chlorogenic acid, parishin B, parishin C, ferulic acid, parishin A, and quercetin, with UV detection conducted at 254 nm. **(B)** HPLC chromatogram of TMGT, with UV detection conducted at 254 nm. **(C)** Data showing reproducibility of HPLC fingerprints for 10 batches of TMGT (S1-S10) are shown. The *X*-axis represents the retention time, and the *Y*-axis represents signal strength.

We obtained TMGT lyophilized powder from the Research Center of Traditional Chinese Medicine Affiliated Hospital of Changchun University of Chinese Medicine (Changchun, China). Each medicinal material used for experiments was tested by the Chinese Medicine Appraisal Laboratory of Changchun University of Chinese Medicine. The final yield of the aqueous extract of TMGT was 43.19%. The aqueous extract of TMGT was stored at −80°C before use. TMGT powder (prepared as previously described) was dissolved in 50% methanol made with ultrapure water to generate a solution of concentration 70 mg/ml for use in HPLC analysis (Deng et al., 2020). Eight different batches of TMGT powder were subjected to chromatographic separation using a ZORBAX 300 Exten-C18 column (4.6 × 250 mm, 5 μm, Agilent, CA, United States) then were analyzed for chemical composition using an HPLC system (LC-2030c, Shimadzu Corporation, Kyoto, Japan) coupled with an ultraviolet-visible (UV) detector (Lu et al., 2020). Acetonitrile (Mobile phase A) and 0.1% phosphoric acid in water (Mobile phase B) served as HPLC mobile phases (Supplementary Table S1). The flow rate was 1.0 ml/min at 30°C, and the detection wavelength was set to 254 nm. Gastrodin, parishin A, parishin B, parishin C, chlorogenic acid, ferulic acid, quercetin, and *p*-hydroxybenzyl alcohol served as known reference standards for use in compositional analysis of TMGT based on retention time (Figure 1A, Shanghai Yuanye Biotechnology, Shanghai, China). Chemical constituents corresponding to eight major HPLC peaks obtained for TMGT extract were identified based on comparisons of retention times to those of known standards (Figure 1B). Relative proportions of gastrodin, *p*-hydroxybenzyl alcohol, chlorogenic acid, parishin B, parishin C, ferulic acid, parishin A, and quercetin were 9.929, 0.382, 1.806, 0.078, 0.035, 0.045, 0.060, and 0.005%, respectively. Quality assessments of TMGT preparations, which were conducted using Chinese Medicine Chromatographic Fingerprint Similarity Evaluation System (2012 Edition) guidelines, demonstrated a high degree of overall TMGT batch-to-batch reproducibility, as reflected by the results of HPLC fingerprint analysis (Figure 1C). In fact, for all eight TMGT batches, the overall similarity exceeded 98%, thus demonstrating batch-to-batch consistency and reproducibility of TMGT preparations generated in this study.

### Construction of Multiple Interaction Network Between TMGT and CI

Keywords of “GouTeng,” representing GT associated active ingredients, and “TianMa,” representing TM-associated active ingredients, were used to search TCMSP (http://tcmspw.com/) and BATMAN-TCM (http://bionet.ncpsb.org.cn/batman-tcm/) databases, respectively (An et al., 2020). Next, we screened the GeneCards database (https://auth.lifemapsc.com/) to obtain CI-related proteins, and transcriptomic information (Wang Y. et al., 2020; Chen et al., 2021). Based on the abovementioned screening results, we drew a Venn diagram to display the interaction networks of TMGT-associated components and CI-related targets using Venny (2.1.0) (http://bioinformatics.psb.ugent.be/webtools/venn/) (Wang et al., 2021). Finally, we constructed the interaction network of chemical composition-targets of action, and the potential targets of TMGT against CI using Cytoscape (3.6.1) (Xu et al., 2021).

### Enrichment Analysis of Genes and Pathways

GO enrichment analysis was performed to predict possible functions of target genes using the DAVID 6.8 database (https://david.ncifcrf.gov/). KEGG (Kyoto Encyclopedia of Genes and Genes) pathway enrichment was carried out to predict pathways associated with proteins corresponding to genes predicted using OmicShare tools (https://www.omicshare.com/tools/) (Xu et al., 2020). *Homo sapiens* was selected as the species item, and *p <* 0.05 was chosen as the screening condition.

### Construction of Molecular Docking Models

Interactions between bioactive ingredients of TMGT and CI-associated target genes were verified through the construction of molecular docking models using the Maestro 11.9 software (Li et al., 2021). In brief, the crystal structures of the target proteins corresponding to predicted target genes were first downloaded from the RCSB Protein Data Bank database (https://www.rcsb.org/) then were imported into Maestro. Next, water molecules and irrelevant ligands in the crystal structure were systematically removed by adding hydrogen atoms using OPLS3e force field, then the adjusted crystal structures were optimized by determining their energy-minimized conformations. As described previously, structures of active ingredients of TGMT were obtained from the PubChem database (https://pubchem.ncbi.nlm.nih.gov/) and were saved in SDF format (Yuan et al., 2021). Using the LigPrep module of Maestro, the energy-minimized conformation was selected using force-field OPLS3e and Epik set to pH 7.0 ± 2.0 to produce the candidate ligand for docking. The setting “Dock ligands with length” of the Glide module was defined as 15 Å for use in creating a binding pocket. Finally, the XP docking method in the ligand docking module was selected for docking analysis, and the RMSD (root mean square deviation) value was chosen to evaluate the displacement of the two state coordinates before and after docking. The Glide Score was selected to evaluate the binding of the ligand and the receptor, and the PyMOL software was chosen to visualize the top-ranked compounds.

### Cell Culture and Treatment

The BV2 and PC12 cell lines were purchased from The American Type Culture Collection (ATCC, Rockefeller, Maryland, United States). BV2 cell lines were cultured in MEM with 10% fetal bovine serum (FBS, CLARK Bioscience, Claymont, DE, United States), while PC12 cell lines were cultured in DMEM (Gibco, New York, NY, United States) supplemented with 7.5% FBS (CLARK Bioscience) and 2.5% horse serum (Gibco). Both cell lines were cultured in a medium supplemented with penicillin-streptomycin (each at 100 Units/ml, MedChemExpress, Shanghai, China) at 37°C in an incubator with humidified atmosphere and 5% CO_2_. Before they were used in the experiments, PC12 cells were incubated with 50 ng/ml nerve growth factor (NGF, Gibco) for 24 h to induce neurite formation in order to establish the neuronal cell model (Hu et al., 2018; Huang et al., 2018). To establish OGD-induced cell injury models, differentiated PC12 cells were maintained in a serum-free, glucose-free medium and incubated under oxygen-free conditions (95% N_2_, 5% CO_2_) in a BioSpa automated incubator (BioTek, United States) for 0–8 h. Cells of the control group were cultured in a normal medium, while cells in OGD or LPS model groups were subjected to OGD or LPS exposure, respectively. For treatment groups, TMGT or quercetin (40 μM) extracts were dissolved in DMSO to create high-concentration stock solutions that were further diluted to generate solutions of various concentrations according to the requirements of individual experiments. Next, cells were pretreated with different concentrations of TMGT or quercetin for 24 h followed by exposure to OGD conditions or LPS (Le et al., 2020).

### Cell Viability Assay

PC12 or BV2 cells were seeded into wells of a 96-well plate at a density of 3 × 10^3^ cells/well, then a 100-μl DMEM/MEM medium was added to each well. PC12 cells were treated with TMGT or quercetin (PHR1488, Sigma-Aldrich) after the cells grew to 80% confluence in each well followed by incubation for 24 h. Untreated cells cultured under OGD conditions (for the same amounts of time as for treated cells) served as controls. Next, 3-(4,5-dimethylthiazol-2-yl)-2,5-diphenyltetrazolium bromide (MTT, 5 mg/ml, Sigma-Aldrich) was added to cells to determine cell viability according to the instructions provided with the kit. A total concentration of 10% MTT (v/v) was added to cells in different groups, then DMSO (150 μl) was added to each well to dissolve the cells (Babaei et al., 2021). The OD value of each well was detected using a microplate reader (TECAN Infinite M200pro, ZH, Switzerland) at a wavelength of 490 nm.

### Lactate Dehydrogenase Detection

PC12 cells were seeded into wells of 96-well plates at a density of 3 × 10^3^ cells/well and exposed to OGD conditions for 2 h followed by no treatment or treatment with TMGT or quercetin for 24 h. LDH release was measured using an assay kit to detect levels of LDH in supernatants of PC12 cells (Jin et al., 2019). Based on absorbance values at 450 nm measured according to the manufacturer’s instructions.

### Enzyme-Linked Immunosorbent Assay

Supernatants of OGD-induced PC12 cells and LPS-induced BV2 cells treated with different concentrations of TMGT or quercetin were collected. ELISAs were performed to evaluate the levels of the inflammatory factors (IL-6, IL-1β, and TNF-α) in PC12 and BV2 cells according to the manufacturer's instructions (He et al., 2017). ELISA kits to detect IL-6 (# BMS603-2, # ERA31RBX5), IL-1β (# BMS6002TEN, # BMS630TEN), and TNF-α (# 88-7324-86, # KRC3011C) were obtained from Thermo Fisher Scientific (Waltham, MA, United States).

### Western Blotting

PC12/BV2 cells were treated with RIPA lysate buffer containing a protease inhibitor (Roche, Basel, Switzerland) and a phosphatase inhibitor (Roche). Concentrations of proteins within supernatants were determined using a BCA kit (Roche). Proteins (35 μg per lane) were loaded into wells of SDS-PAGE gels followed by electrophoresis to separate proteins by molecular weight (8–10% SDS-PAGE separation gel and a 5% SDS-PAGE concentrated gel). Next, proteins were transferred to polyvinylidene fluoride membranes (PVDF, Roche) using a wet transfer method. PVDF membranes bound to proteins were blocked by immersion in 5% (w/v) bovine serum albumin (BSA), then membranes were probed with antibodies anti-IκBα, ab76429; anti-HIF-1 alpha, ab179483, anti-IKB alpha (phospho S36), ab133462; Abcam, Cambridge, MA, United States; anti-NF-κB p65, #8242; anti-phospho-NF-κB p65 (Ser536), #3033; anti-β-Actin, #4970 (Cell Signaling Technology, Beverly, MA, United States); and anti-PPAR gamma, bs-0530R (Bioss, Beijing, China) as dilutions of 1:1,000 followed by incubation at 4°C overnight. Next, membranes were incubated with secondary antibody (HRP) at room temperature for 1 h. An enhanced ECL reagent was added to membranes to visualize target proteins attached to membranes (Qi et al., 2019). Antibody-binding to target protein as a measure of the relative protein expression level was calculated by measuring areas and intensities of bands using AlphaView SA.

### Statistical Analysis

GraphPad Prism 6.0 software (San Diego, California, United States) was used for all data analysis. Experiments in this study were repeated three times. The one-way ANOVA test (Turkey’s post hoc) was chosen to calculate the significance of multiple comparisons. The level of significance was set to *p* < 0.05.

## Results

### Interaction Networks of TMGT Related Active Ingredient and CT Associated Targets

**FIGURE 2:**
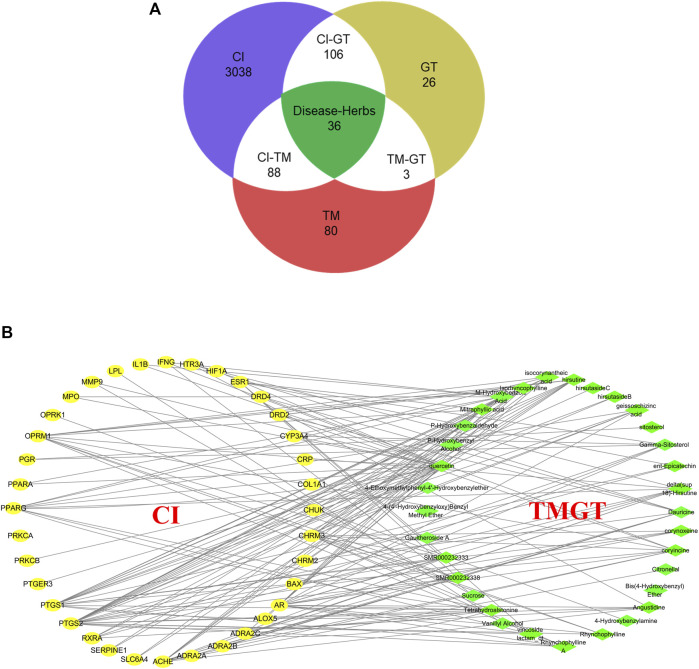
Interaction network of TMGT-related active ingredient and CI-associated targets. **(A)** Venn diagram describing target distribution of TMGT and cerebral infarction. (TM, Gastrodia, Tianma in Chinese; GT, Uncaria, Gouteng in Chinese; CI, cerebral infarction) **(B)** The network of a putative component target. TMGT active ingredients are colored green, and target proteins associated with CI are colored yellow. Each edge identifies the relationship between the active ingredient molecule and the target protein.

Screening of published literature for information regarding TMGT active ingredients using the keywords “TianMa” and “GouTeng” led to the identification of a total of 46 active ingredients (Supplementary Table S2). These ingredients were subsequently verified through searches of TCMSP and BATMAN-TCM databases based on the criteria that included oral bioavailability OB ≥30% and drug-like DL ≥0.18. Meanwhile, a total of 3,268 genes were searched for CI-related targets after entering “Cerebral Infarction” in GeneCards, then the results were subjected to VENNY analysis, which led to the identification of 36 common targets of CI and TMGT, as shown in Figure 2A. Ultimately, the selected 46 compounds and 36 targets were imported into Cytoscape (3.6.1) to generate an interaction network (Figure 2B) containing 69 nodes and 169 sidebars.

### PPI Network Construction

**FIGURE 3:**
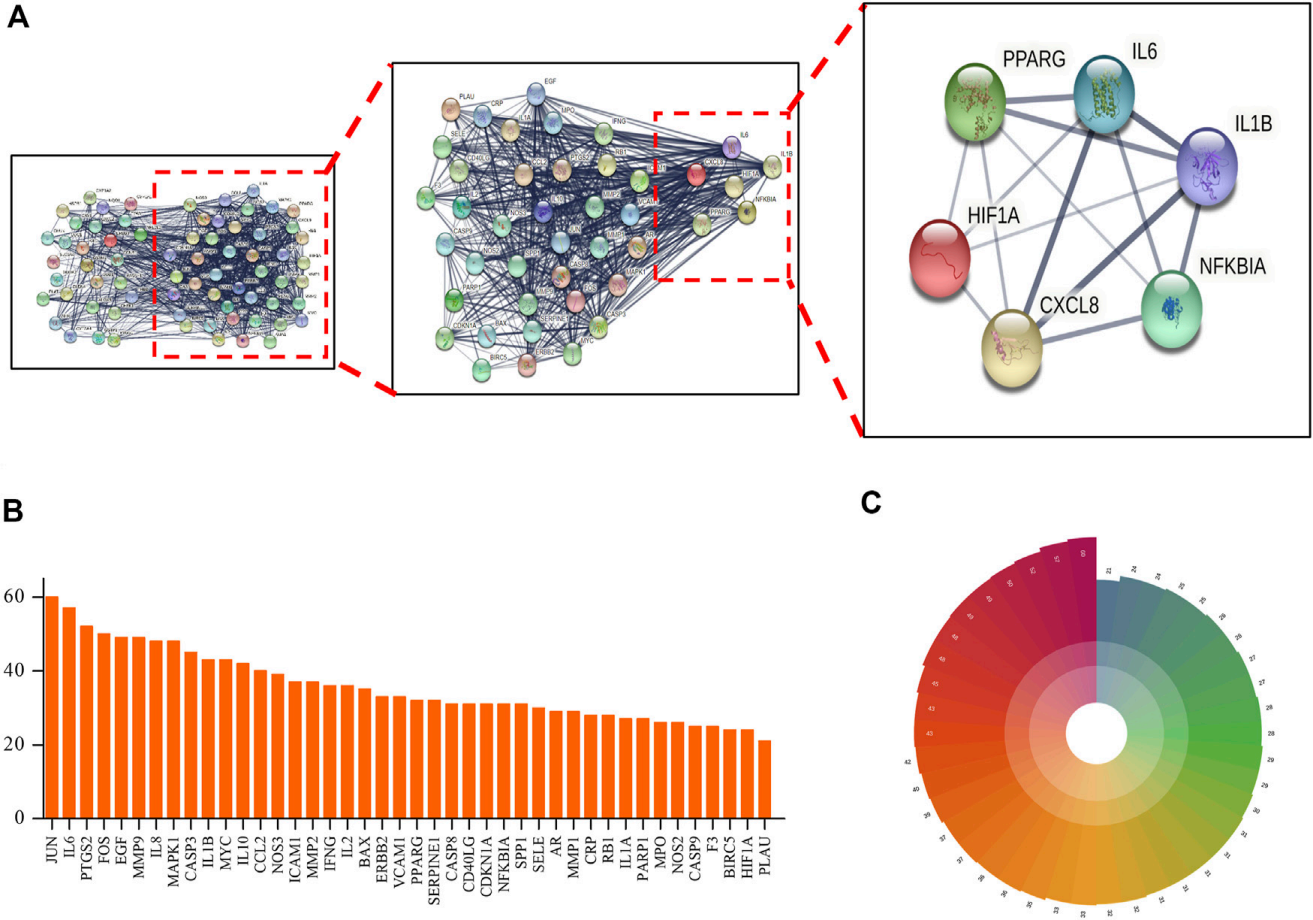
Protein–protein interaction (PPI) network of TMGT target for the treatment of cerebral infarction. **(A)** STRING analysis of the PPI network. The circles represent genes; the results inside the circles represent protein structures. Lines represent interactions between protein targets, with line thickness indicating strength of supporting data. **(B)** Statistical analysis of neighboring nodes in the PPI network. The *X*-axis indicates the name of protein, and the *Y*-axis indicates the number of interconnected network neighboring nodes. **(C)** Polar bar histogram. Different colors represent different groups. The length of the column indicates the relative magnitude of grouped data statistical significance (the longer the column, the more significant the data).

Target genes identified from analysis of interaction networks (mentioned in Figure 3) were input into STRING (https://string-db.org/) to generate the protein–protein interaction (PPI) network. Next, indirect connections were made based on reported functions of selected target proteins or protein characteristics obtained from related published studies (Figure 3A), resulting in the creation of a PPI network containing 70 nodes, with neighboring nodes >20 in the PPI network listed (Figures 3B,C). Among them, JUN protein had the highest score due to its connections with 60 adjacent nodes. Putative close relationships among adjacent nodes were predicted using a polar coordinate histogram. We speculate that targets identified through this analysis may play important roles in mechanisms underlying beneficial TMGT effects on CI-induced CNS cell damage.

### GO and KEGG Enrichment Analyses

**FIGURE 4:**
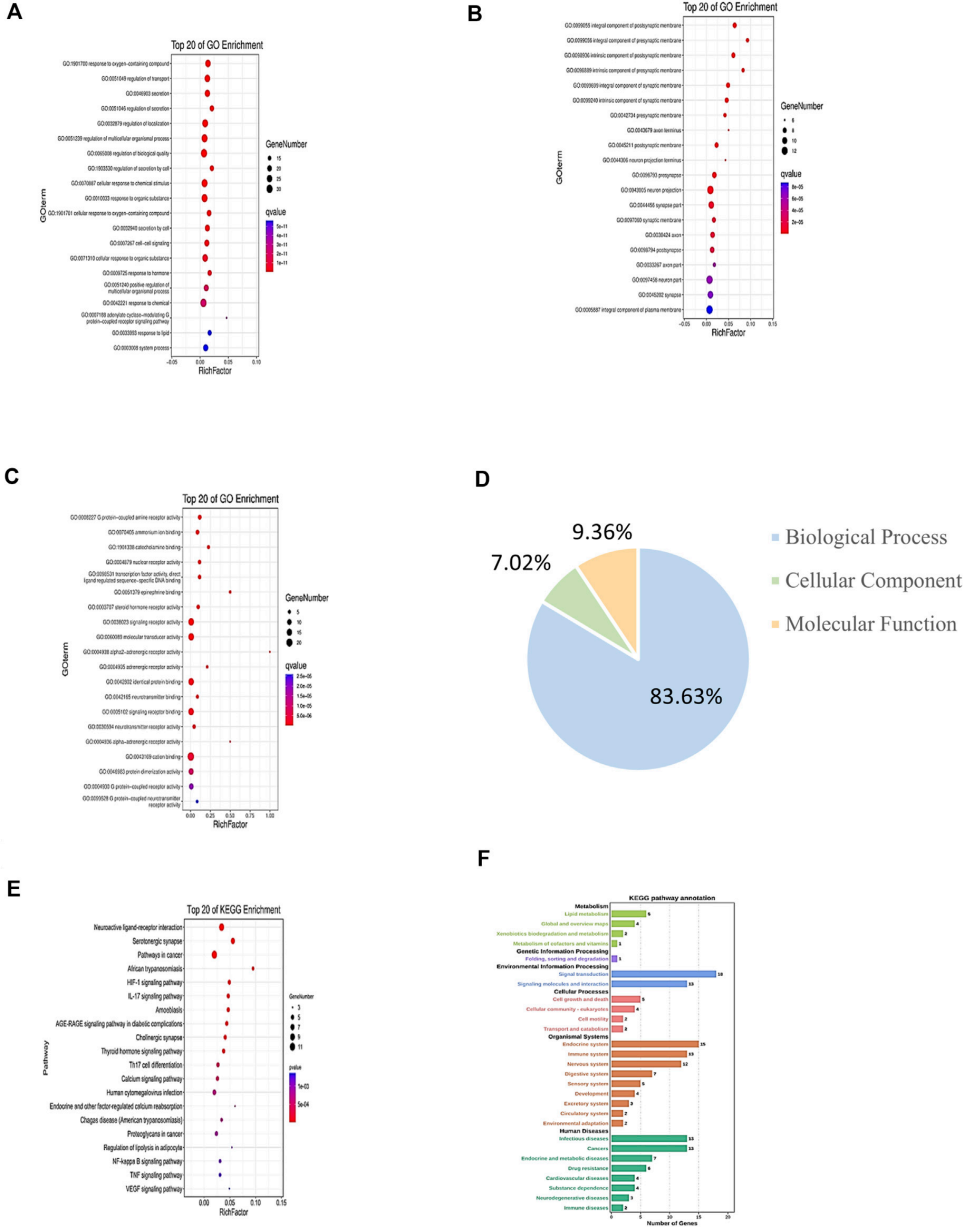
GO and KEGG enrichment analyses of target genes of CI under TMGT treatment. GO annotation results are displayed under three main categories: **(A)** biological process, **(B)** molecular function, and **(C)** cellular component. **(D)** Three types of GO gene and gene product attributes in database (blue: biological process; orange: molecular function; green: cellular component); KEGG pathway enrichment analysis of targets: **(E)** KEGG enrichment analysis of pathways for the top 20 targets. **(F)** KEGG pathway annotation. The bubble size represents the number of genes enriched passage; the larger the bubble, the more enriched the genes. Bubble colors represent significant enrichment; the redder the color, the higher the degree of enrichment.

For GO enrichment analysis, a total of 3,848 GO entries were obtained for the 46 common targets. Results showed that 83.63% of targets were functionally associated with “Biological Process (BP),” 7.02% with “Cellular Component” (CC), and 9.36% with “Molecular Function” (MF). However, the proportions of the three abovementioned categories were listed as 54.42, 3.38, and 5.46% for results with *p* values <0.05. The top 20 molecules were selected for use in drawing a bubble chart (Figure 4). The results suggested that the top 20 molecules were mainly related to neuron projection terminus, neurotransmitter binding, and positive regulation of multicellular organismal process. Meanwhile, KEGG pathways were shown to consist of aggregates of pathway maps based on current knowledge of molecular interaction networks (Su et al., 2018). Followed by the KEGG enrichment pathway, 169 genes functionally associated with key pathways were obtained, among which 46.75% exhibited differential expression characters. The top 20 selected pathways are shown in Supplementary Table S3. These results revealed that key pathways associated with common target proteins were mainly related to functional terms neuroactive ligand–receptor interaction, the HIF-1 signaling pathway, and the NF-kappa B signaling pathway (Figures 4E,F).

### Molecular Docking

**FIGURE 5:**
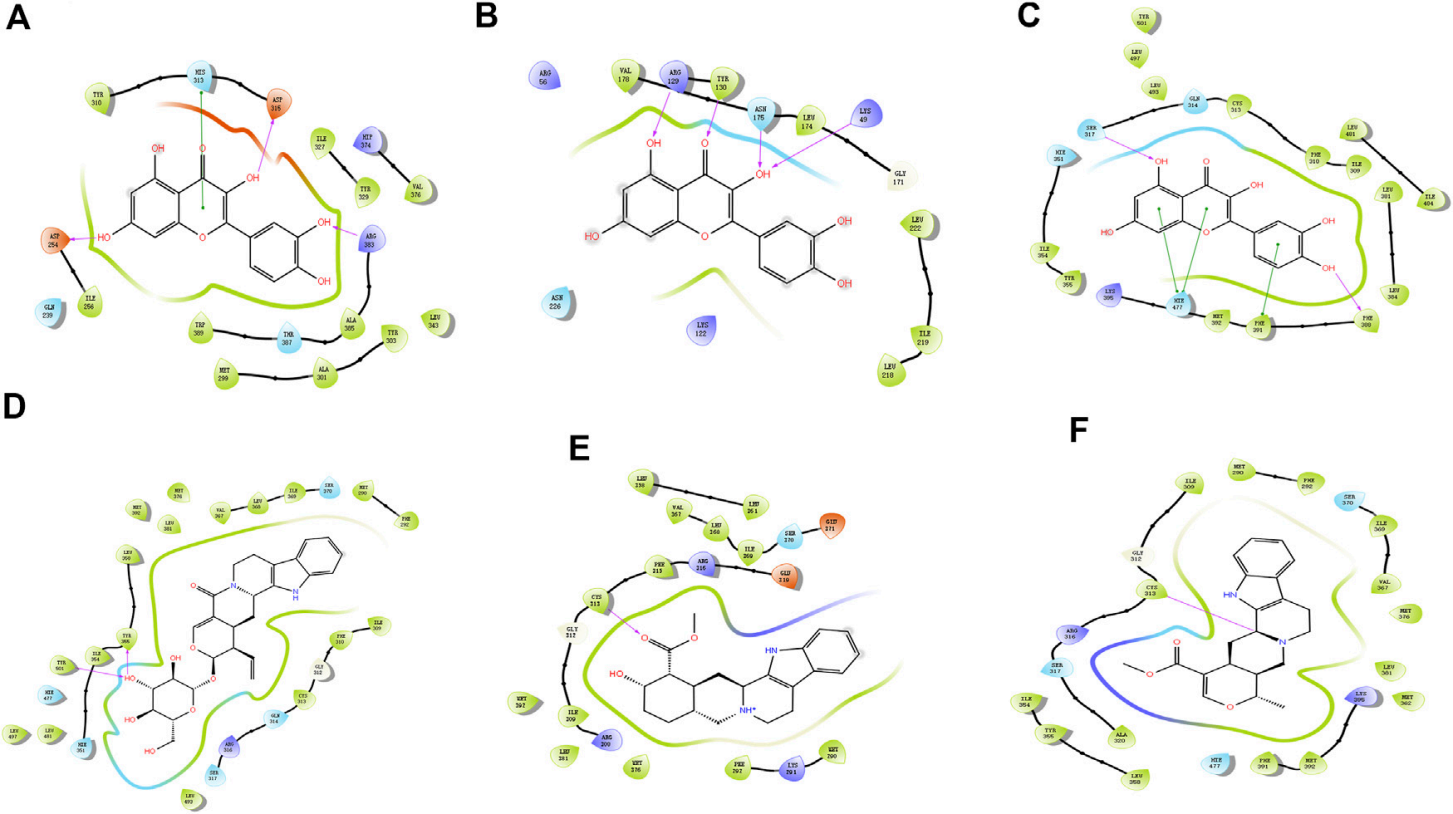
Molecular docking map. **(A)** HIF1A (HIF-1α)-quercetin. **(B)** NFKBIA (IκBα)-quercetin. **(C)** PPARG (PPARγ)-quercetin. **(D)** PPARG (PPARγ)-Vincoside lactam_qt. **(E)** PPARG (PPARγ)-Yohimbine. **(F)** PPARG (PPARγ)-Tetrahydroalstonine. The color indicates the type of residue: red—acidic, green—hydrophobic, purple—basic, and blue—polar. Protein–ligand interactions are indicated as lines between ligand atoms and protein residues: purple represents H-bonding to the protein backbone, while green represents pi-pi stacking interactions involving side chains of proteins. Gray spheres are used to mark ligand atoms exposed to solvent.

Molecular docking technology is a common method for exploring functions/effects of drugs (Li et al., 2021). Here we chose core proteins for docking studies, including PPARG, HIF1A, and NFKBIA, which were identified from the PPI network as research objects (Figure 5). TMGT active ingredients that interacted with these three core proteins were molecularly docked. Figure 5 shows the 2D interaction molecular docking diagram. From Figures 5A–C, it can be seen that quercetin could form hydrogen bonds with several amino acid residues of HIF1A (Figure 5A), NFKBIA (Figure 5B), and PPARG (Figure 5C) to form stable spatial structures (with binding energies <-5 kcal/mol, mentioned in Supplementary Table S4). Thus, quercetin may be a key bioactive core ingredient of TMGT. Meanwhile, PPARG also bound stably to vincoside lactam_qt (Figure 5D), yohimbine (Figure 5E), and tetrahydroalstonine (Figure 5F). PPARG bound most strongly with the indole alkaloid vincoside lactam_qt (with binding energies = 11.54 kcal/mol), a component commonly incorporated in anti-inflammatory and antivasoconstriction drugs, thus suggesting that TMGT may exert beneficial blood vessel dilation and anti-inflammatory effects. Notably, the root-mean-square deviation (RMSD) values of all of the abovementioned molecular docking models were <2Å, thus demonstrating the reliability of these models.

### Protective Effect of TMGT on Cell Viability and LDH Leakage

**FIGURE 6:**
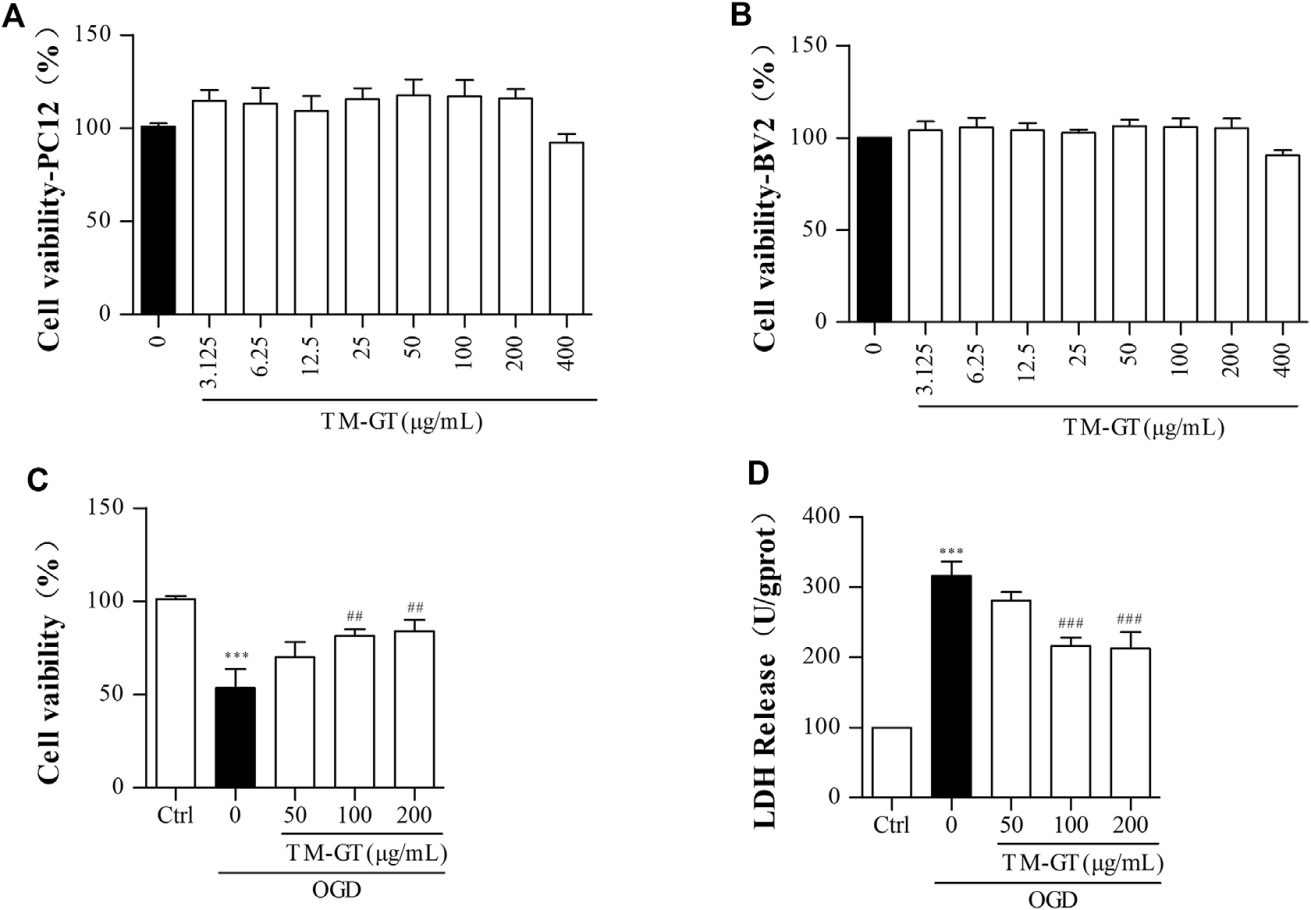
Effects of TM-GT on cell viability and LDH leakage in different cells. After the pretreatment with different concentrations of TMGT for 24 h **(A)** PC12 cells was measured by MTT assay of cell viability effect of TMGT. **(B)** BV2 cells were measured by MTT assay of cell viability effect of TMGT. **(C, D)** Effect of TMGT on the cell viability and LDH leakage in OGD-induced PC12 cells. Cells in the OGD model group were cultured under OGD conditions for 2 h, and cells in treatment groups were exposed to different concentrations of TMGT for 24 h prior to culture under OGD conditions. ^*∗∗∗*^ *p* < 0.001 versus the control group; ^*##*^ *p* < 0.01 and ^*###*^ *p* < 0.001 versus the OGD group.

As shown in Figures 6A,B, we first conducted MTT assays to confirm that TMGT treatment did not decrease the viability of PC12 and BV2 cells. Results showed that 24-h TMGT treatment concentrations ranging from 3.125 to 400 μg/ml had no significant effect on the survival of PC12 and BV2 cells. To establish OGD models, PC12 cells were incubated under anaerobic (95% N_2_, 5% CO_2_) and low-glucose conditions for different amounts of time. As shown in Supplementary Figure S1A, cells cultured under OGD conditions exhibited decreased cell viability in a time-dependent manner based on OGD exposure duration. Moreover, 2-h cell culture under OGD conditions obviously decreased cell viability (to 53.77%) as compared to control group viability. As shown in Figure 6C, OGD-induced decline in cell viability was reversed by cell treatment with various concentrations of TMGT. Specifically, cell viability could be greatly increased by as much as 1.31-fold by treatment with a TMGT concentration of 50 μg/ml, suggesting protective effects of TMGT against OCD-induced PC12 cell damage. Also, OGD exposure of PC12 cells led to greatly increased LDH release by the cells as compared to that of control cells. By contrast, dramatically decreased LDH release by PC12 cells (by 0.67- to 0.89-fold) was observed after TMGT treatment (Figure 6C), suggesting that TMGT treatment protected PC12 cells from OCD-induced damage.

### TMGT Inhibited the Inflammatory Response in PC12 Cells and BV2 Cells

**FIGURE 7:**
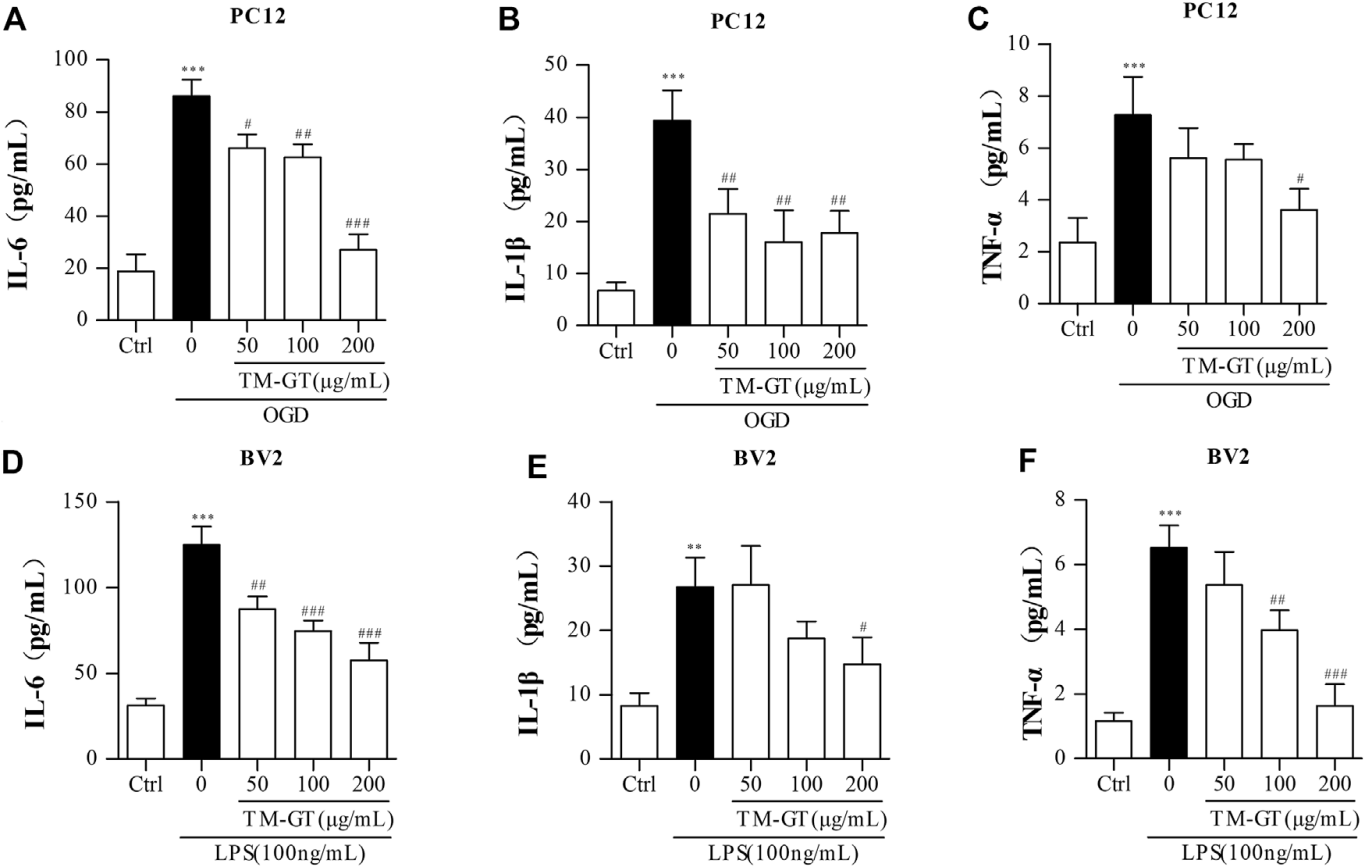
Detection of inflammatory factors. **(A–C)** After PC12 cells were pretreated with TMGT for 24 h, they were cultured under OGD conditions for 2 h, then levels of IL-6, IL-1β, and TNF-α in the supernatants of PC12 cells were detected by ELISA kits. **(D–F)** After the pretreatment with different concentrations of TMGT for 24 h followed stimulation with 100 ng/ml LPS for 6 h, the levels of IL-6, IL-1β, and TNF-α in the supernatant liquid of BV2 cells were detected by ELISA kits. ^*∗*^ *p* < 0.05 and ^∗*∗∗*^ *p* < 0.001 versus the control group; ^*#*^ *p* < 0.05, ^*##*^ *p* < 0.01, and ^*###*^ *p* < 0.001 versus the LPS or OGD group.

In general, the occurrence of diseases is accompanied by inflammatory biological processes. Based on the aforementioned MTT results and the results of experiments performed using PC12 cells, we found that 2-h culture of cells under OGD conditions significantly increased IL-6 and TNF-α release by PC12 cells (Supplementary Figures S2A,B). We also conducted ELISAs to assess levels of inflammatory factors in PC12 cells under TMGT treatment. Figures 7A–C show that quantities of IL-6, IL-1β, and TNF-α secreted by PC12 cells cultured under OGD conditions were all significantly increased (relative to quantities secreted by control cells without OGD exposure); however, markedly lower quantities of all three factors were secreted by cells that had been pretreated with TMGT prior to culture under OGD conditions.

To establish a microglial inflammation model, BV2 cells were treated with different concentrations of LPS for various amounts of time, then IL-6 and TNF-α release from the cells was measured *via* ELISAs. Results showed significantly increased release of IL-6 and TNF-α after cells were exposed to 100 ng/ml of LPS for 6 h (Supplementary Figures S2C,D), with decreased release of IL-6, IL-1β, and TNF-α observed in cells pretreated with TGMT prior to LPS exposure in a TMGT-dose-dependent manner (Figures 7D–F). Taken together, these findings suggested that TMGT treatment may inhibit CI-induced neuroinflammation.

### TMGT Inhibited PC12 Cell- and BV2 Cell Associated Inflammatory Responses Through the HIF-1α/PPARγ/NF-κB Pathway

**FIGURE 8:**
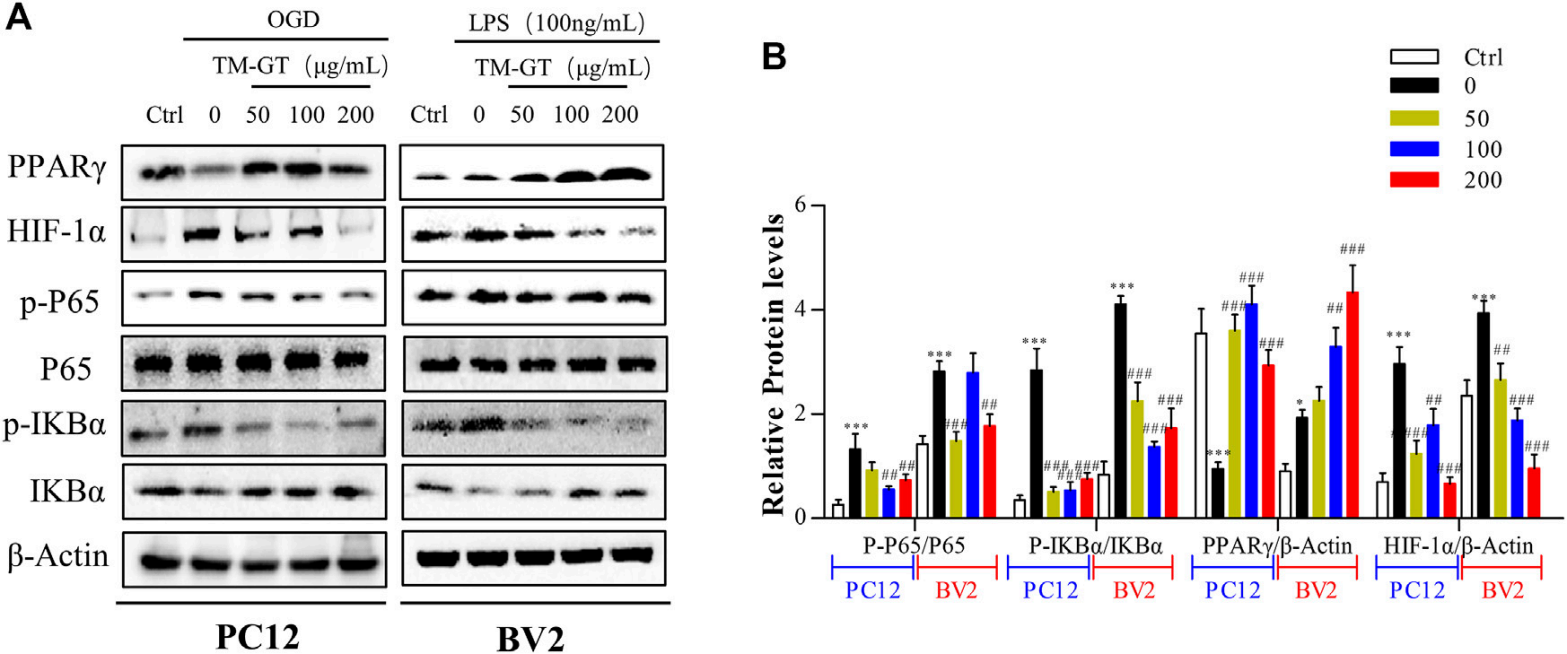
Effects of TMGT on the HIF-1α/PPARγ/NF-κB pathway in OGD-induced PC12 cells and LPS-induced BV2cells. **(A)** Western blot analysis of the protein expression of HIF-1α, PPARγ, and the nuclear and cytosolic P65, IκBα, and *p*-IκBα with β-actin as the respective internal standards. **(B)** Quantitative analysis of the protein expression levels of p-P65/P65, *p*-IκBα/IκBα, PPARγ, and HIF-1α relative to β-actin. The bands were quantified with AlphaView SA, and the intensity of each protein band was normalized to the intensity of β-actin. ^*∗*^ *p* < 0.05 and ^*∗∗∗*^ *p* < 0.001 versus the Ctrl group; ^*##*^ *p* < 0.01, ^*##*^ *p* < 0.01, and ^*###*^ *p* < 0.001 versus the LPS or OGD group.

Based on abovementioned network pharmacology results, the HIF-1α/PPARγ/NF-κB signaling pathway may be the key pathway targeted by TMGT to protecting effects against CNS damage induced by CI. Therefore, we focused on this signaling pathway in our subsequent investigation of signaling-based mechanisms underlying beneficial TMGT effects on OGD-exposed PC12 cells and LPS-exposed BV2 cells. Previous evidence obtained in other studies had pointed out that modulation of PPARγ/NF-κB signaling pathway activity prevented neuronal damage after craniocerebral injury by suppressing intracellular inflammation (Song et al., 2018; Feng et al., 2020). In this work, we found, as shown in Figure 8, that levels of PPARγ were significantly decreased, while *p*-IκBα levels were markedly increased either in OGD-induced PC12 cells or in LPS-induced BV2 cells. After TMGT treatment, levels of activated p-P65, HIF-1α, and IKBα were all significantly altered in a TMGT concentration-dependent manner relative to model group levels, in accordance with predicted pharmacology network analysis results. Thus, these data implied that TMGT intervention may alleviate CI-triggered neuronal and microglial cell damage and neuroinflammatory processes by regulating the HIF-1α/PPARγ/NF-κB pathway.

### Quercetin Inhibited Nerve Damage in PC12 Cells and BV2 Cells

**FIGURE 9:**
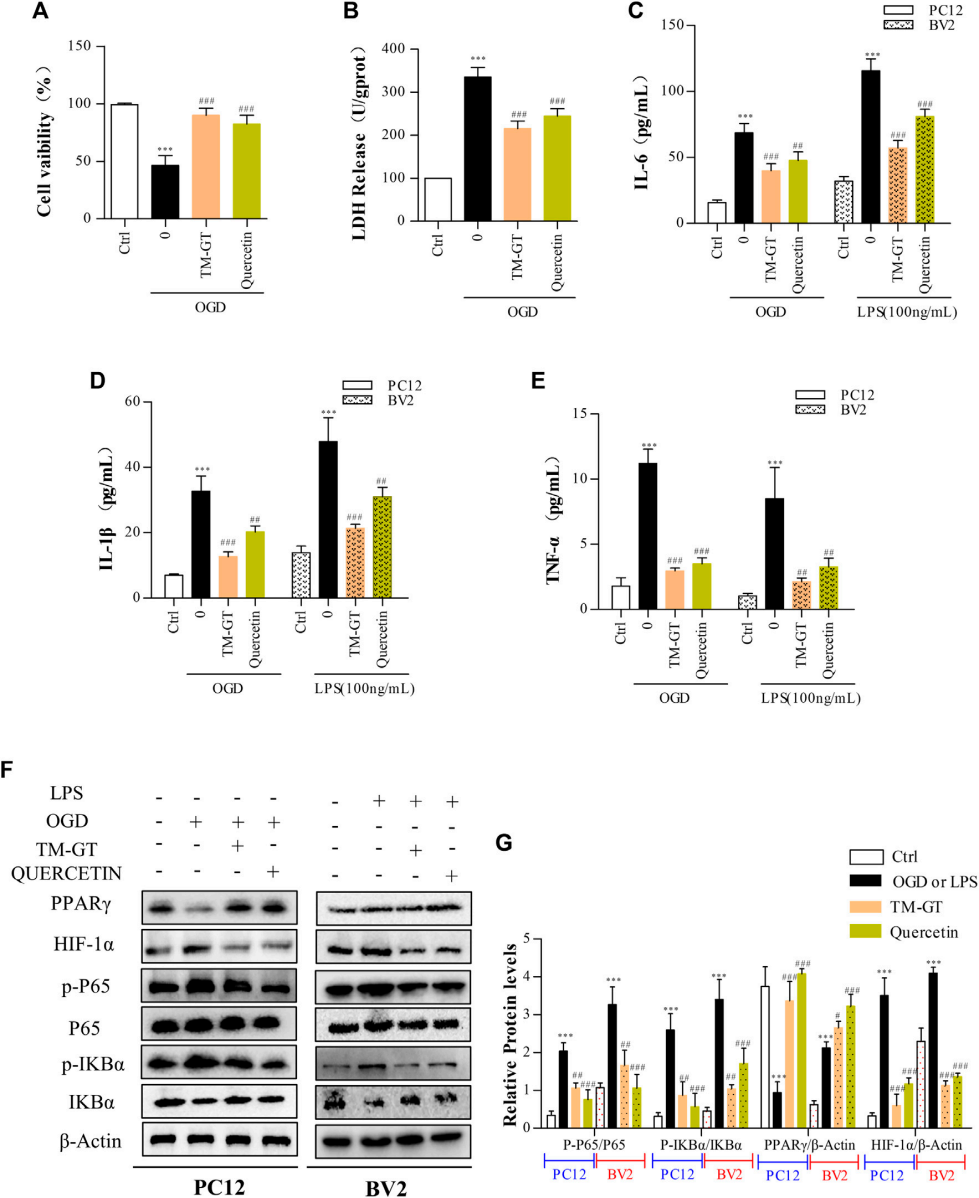
Intervention of quercetin on cerebral infarction. Methods used for quercetin (40 μM) administration and modeling were equivalent to corresponding TMGT methods. **(A, B)** Effect of quercetin on cell viability and LDH leakage from OGD-induced PC12 cells. **(C–E)** After quercetin pretreatment, the levels of IL-6, IL-1β, and TNF-α in supernatants of PC12 cells and BV2 cells were detected using ELISA kits. **(F, G)** Western blot analysis of protein expression of HIF-1α, PPARγ, nuclear and cytosolic p65, IκBα, and *p*-IκBα with β-actin as the respective internal standards. ^*∗∗*^ *p* < 0.01 and ^*∗∗∗*^ *p* < 0.001 versus the Ctrl group; ^*#*^ *p* < 0.05, ^*##*^ *p* < 0.01, and ^*###*^ *p* < 0.001 versus the LPS or OGD group.

According to our molecular docking-based predictions, quercetin may be a key bioactive component involved in TMGT prevention of CI-induced CNS nerve damage, aligning with a previous study. That study reported that quercetin could reduce neurocytotoxicity under OGD conditions through its effects on the ERK/AKT pathway, while also exerting anti-inflammatory and anti-apoptotic effects by inhibiting the activation of MCAO-induced apoptotic pathway signal molecules caspase-3 and PARP, thereby preventing functional impairment of nerve cells (Park et al., 2018; Wang YY. et al., 2020). In a similar vein, evaluation of TMGT effects on OGD-induced PC12 cells (89.95%) revealed an increase in cell viability to 82.40% after quercetin treatment (Figure 9A), and no significant difference was found between effects observed for TMGT and quercetin on cell viability. Similar effects of quercetin in LDH levels were discovered in OGD-induced PC12 cells (Figure 9B). To further explore the relationship between quercetin and nerve inflammation, the expression levels of inflammatory factors were evaluated using two cell lines based on models of CI. As shown in Figures 9C,D, it can be seen that quercetin has an inhibitory effect on the inflammatory factors secreted by OGD-induced PC12 cells and LPS-induced BV2 cells, but it is not as effective as TMGT. Using this strategy, the expression levels of HIF-1α, P65, and PPARγ were tested in cells treated with or without quercetin to uncover the putative regulatory pathway acted on by quercetin to prevent neuronal damage and neuroinflammation (Figures 9F,G). These results suggested that quercetin treatment could prevent neuronal damage and CI-induced inflammatory processes by regulating HIF-1α/PPARγ/NF-κB pathway activity. The abovementioned results thus verify our previous hypothesis that quercetin is a key bioactive component of TMGT that may exert beneficial effects for alleviating cellular dysfunction and neuroinflammation induced by CI.

## Discussion

**FIGURE 10:**
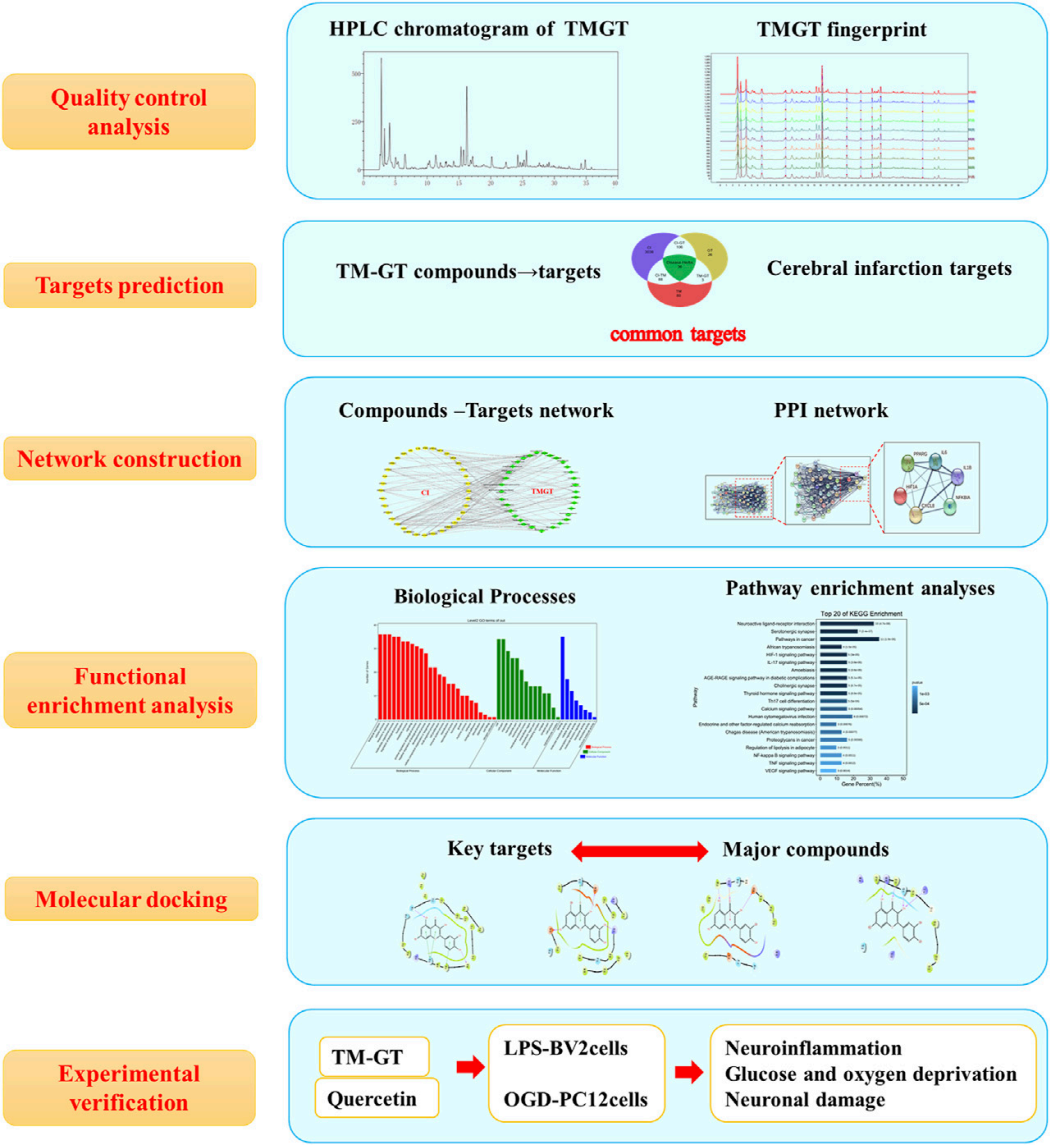
Flowchart map showing the experiment approach for determining the pharmacological and mechanism of TMGT used for the treatment of cerebral infarction by integrating target identification, network construction, enrichment analysis, molecular docking, and experimental validation study.

CI is a common neuroinflammatory disease caused by complex underlying pathological processes. TMGT is a TCM formulation that is widely used for the treatment of CI. In this study, we investigated possible correlations between TMGT bioactive effects and mechanisms for alleviating CNS damage induced by CI (Figure 10). Network pharmacology analysis showed that TMGT effects overlapped with CI-associated processes of neuroinflammation, angiogenesis, and cell apoptosis. Subsequent *in vitro* experiments revealed that inflammatory responses were inhibited and neuronal hypoxia damage was alleviated after TMGT treatment of OGD-exposed PC12 cells and LPS-exposed BV2 cells. Molecular docking technology was introduced to analyze the possible binding ability of CI-associated core targets to TMGT constituent compounds. The results of this analysis indicated that quercetin, tetrahydroalstonine, yohimbine, and vincoside lactam_qt were major TMGT components with strong binding affinities to CI-associated target proteins. Therefore, we evaluated quercetin, a flavonoid compound possessing chemical groups with the strongest predicted binding strengths to CI-associated core targets, to investigate possible molecular mechanisms underlying TMGT beneficial effects on OGD-induced PC12 and LPS-stimulated BV2 cellular functions. Collectively, our results demonstrated that quercetin treatment increased the viability of OGD-exposed PC12 cells, inhibited the release of inflammatory factors, and regulated the expression of key HIF-1α/PPARγ/NF-κB pathway proteins, thereby suggesting that quercetin was the major active ingredient of TMGT. However, quercetin suppression of inflammatory responses was not as good as suppression achieved using intact TMGT, prompting us to search for additional coactive TMGT components. Based on our molecular docking results, we found that three other TMGT components, tetrahydroalstonine, yohimbine, and vincoside lactam_qt, could also bind strongly to PPARγ. Taken together, these results indicate that in addition to flavonoids, indole alkaloids and other bioactive TMGT compounds may also exert synergistic effects to prevent CI-induced cellular dysfunction and neuroinflammation.

Studies have shown that alkaloids can effectively improve cerebral blood circulation and metabolism, and dilate blood vessels, while also inhibiting atherosclerosis, a cause of Alzheimer’s disease and other cardiovascular and cerebrovascular diseases (Geetha and Ramachandran, 2021). Both indole alkaloids and flavonoids have anti-inflammatory effects and are often incorporated in anti-inflammatory drug formulations (Choi et al., 2021; Yang et al., 2021). Therefore, other TMGT active ingredients and their associated molecular mechanisms for preventing CI-induced cellular dysfunction and neuroinflammation warrant further study.

Inflammatory responses are involved in all stages of stroke-related injury, including early cerebral-infarction tissue repair and regeneration after ischemia (Ghelani Drishti et al., 2021) and neuroinflammation that alters neuronal structure and function (Mehta et al., 2017). Microglial, innate immune cells of the CNS can be activated and polarized to assume various phenotypes in response to stroke-induced stimulatory signals to greatly influence stroke prognosis (Dong et al., 2021). Following CI, microglial activation occurs that leads to significantly increased aggregation of inflammatory cells and secretion of cytokines, with concurrent induction of acute neuroinflammation triggered by neuronal hypoxia within brain tissues. In various *in vitro* experiment models, inflammatory factors, such as TNF-α, IL-1β, and IL-6, have been shown to be associated with CI-induced brain injury (Hovhannesyan and Hovhannisyan, 2019; Jimenez-Almonte et al., 2019). In accordance with the results of previous studies, the results of this study revealed that the levels of inflammatory factors, including TNF-α, IL-1β, and IL-6, were significantly reduced by TMGT treatment.

Intriguingly, our results demonstrated that HIF-1α/PPARγ/NF-κB pathway activity was closely tied to the neuroinflammatory response triggered by CI. Under neuron-damaging conditions, HIF-1α/PPARγ/NF-κB signal pathway-related proteins, including HIF-1α, IκBα, and P65, were activated. Activation of these proteins further triggered downstream biological responses that inhibited the expression of inflammatory factors, alleviated glucose and oxygen deprivation response, and reduced the severity of CI-induced brain dysfunction. Based on molecular docking analysis results, three TMGT constituent compounds were shown to bind strongly with PPARγ. Notably, a search of previous studies revealed that an observed neuroprotective effect of PPARγ against CI-induced CNS injury had been reported previously (Wu et al., 2018). In another study, PPARγ functioned as a key regulator of CNS nerve function under conditions of non-infectious chronic inflammation (Machado et al., 2019).

## Conclusion

In this study, HPLC fingerprint analysis was used to detect and identify major chemical components of TMGT. Network pharmacology analysis conducted in this work revealed that the HIF-1α/PPARγ/NF-κB pathway may be associated with the molecular mechanism whereby TMGT alleviates neuronal damage and neuroinflammation induced by CI. Moreover, *in vitro* experiments using PC12 and BV2 cell-based models of CI-induced hypoxia and glucose deprivation damage and neuroinflammation, respectively, showed that TMGT treatment may reduce neuronal cell damage and prevent neuroinflammation. Furthermore, molecular docking experiments verified that quercetin, a component of TMGT, could bind with high affinity to proteins associated with CI-induced CNS damage. Taken together, the findings of this study may provide new insights to better understand TMGT activities and mechanisms of action for protecting CNS cells from damage induced by CI.

## Data Availability

The original contributions presented in the study are included in the article/Supplementary Material, further inquiries can be directed to the corresponding authors.

## Abbreviations

BATMAN-TCM, bioinformatics analysis tool for molecular mechANism of traditional Chinese medicine; CI, cerebral infarction; GO, gene ontology; HIF-1α, hypoxia-inducible factor 1-alpha; HPLC, high-performance liquid chromatography; IL-6, interleukin-6; IL-1β, interleukin-1β; IκBα, IkappaB-Alpha; KEGG, Kyoto Encyclopedia of Genes and Genomes; LDH, lactate dehydrogenase; NF-κB, nuclear factor kappa-B, P65; PPARγ, peroxisome proliferator-activated receptor gamma; PPI, protein–protein interaction; PVDF, polyvinylidene fluoride; TCM, traditional Chinese medicine; TCMSP, TCM systems pharmacology; TMGT, Tianma Gouteng; TNF-α, tumor necrosis factor-alpha.

## References

[B1] An L. Lin Y. Li L. Kong M. Lou Y. Wu J. (2020). Integrating Network Pharmacology and Experimental Validation to Investigate the Effects and Mechanism of Astragalus Flavonoids against Hepatic Fibrosis. Front. Pharmacol. 11, 618262. 10.3389/fphar.2020.618262 33551818 PMC7862122

[B2] Babaei A. Mousavi S. M. Ghasemi M. Pirbonyeh N. Soleimani M. Moattari A. (2021). Gold Nanoparticles Show Potential *In Vitro* Antiviral and Anticancer Activity. Life Sci. 284, 119652. 10.1016/j.lfs.2021.119652 34051217

[B3] Chen Y. Dong J. Yang D. Qian Q. Wang P. Yang X. (2021). Synergistic Network Pharmacology for Traditional Chinese Medicine Liangxue Tongyu Formula in Acute Intracerebral Hemorrhagic Stroke. Neural Plast. 2021, 8874296. 10.1155/2021/8874296 33727915 PMC7936909

[B4] Choi J.-H. Lee E.-B. Jang H.-H. Cha Y.-S. Park Y.-S. Lee S.-H. (2021). Allium Hookeri Extracts Improve Scopolamine-Induced Cognitive Impairment via Activation of the Cholinergic System and Anti-neuroinflammation in Mice. Nutrients 13, 2890. 10.3390/nu13082890 34445062 PMC8400157

[B5] Das A. Sil A. Jaiswal S. Rajeev R. Thole A. Jafferany M. (2020). A Study to Evaluate Depression and Perceived Stress Among Frontline Indian Doctors Combating the COVID-19 Pandemic. Prim. Care Companion CNS Disord. 22, 20m02716. 10.4088/PCC.20m02716 33031651

[B6] Deng L. H. Li L. Zhai Y. Michael S. Yang C. Y. Guo R. (2020). Tianma Gouteng Decoction Exerts Cardiovascular Protection by Upregulating OPG and TRAIL in Spontaneously Hypertensive Rats. Evid. Based Complement. Alternat Med. 2020, 3439191. 10.1155/2020/3439191 33133215 PMC7593748

[B7] Dong R. Huang R. Wang J. Liu H. Xu Z. (2021). Effects of Microglial Activation and Polarization on Brain Injury after Stroke. Front. Neurol. 12, 620948. 10.3389/fneur.2021.620948 34276530 PMC8280287

[B8] Famakin B. M. Vemuganti R. (2020). Toll-Like Receptor 4 Signaling in Focal Cerebral Ischemia: a Focus on the Neurovascular Unit. Mol. Neurobiol. 57, 2690–2701. 10.1007/s12035-020-01906-5 32306272

[B9] Feng D. Zhou H. Jin X. Wei J. Zhang Q. Gu Y. (2020). Electroacupuncture Pretreatment Alleviates LPS-Induced Acute Respiratory Distress Syndrome via Regulating the PPAR Gamma/NF-Kappa B Signaling Pathway. Evid. Based Complement. Alternat Med. 2020, 4594631. 10.1155/2020/4594631 32774418 PMC7396021

[B10] Geetha R. G. Ramachandran S. (2021). Recent Advances in the Anti-inflammatory Activity of Plant-Derived Alkaloid Rhynchophylline in Neurological and Cardiovascular Diseases. Pharmaceutics 13, 1170. 10.3390/pharmaceutics13081170 34452133 PMC8400357

[B31] Ghelani Drishti P. Ah Kim H. Zhang Shenpeng R. (2021). Ischemic Stroke and Infection: A Brief Update on Mechanisms and Potential Therapies. Biochem. Pharmacol. 193. 10.1016/j.bcp.2021.114768 34543657

[B11] Gogoleva V. S. Drutskaya M. S. Atretkhany K. S. (2019). The Role of Microglia in the Homeostasis of the Central Nervous System and Neuroinflammation]. Mol. Biol. (Mosk) 53, 790–798. 10.1134/S0026898419050057 31661478

[B12] Hammond T. R. Marsh S. E. Stevens B. (2019). Immune Signaling in Neurodegeneration. Immunity 50, 955–974. 10.1016/j.immuni.2019.03.016 30995509 PMC6822103

[B13] He J. Lv L. Wang Z. Huo C. Zheng Z. Yin B. (2017). Pulvis Fellis Suis Extract Attenuates Ovalbumin-Induced Airway Inflammation in Murine Model of Asthma. J. Ethnopharmacol. 207, 34–41. 10.1016/j.jep.2017.06.016 28624362

[B14] Hovhannesyan R. A. Hovhannisyan I. G. (2019). Platelet Aggregation and Interleukins Indicators Impacting the Outcomes of Ischemic Stroke. J. Stroke Cerebrovasc. Dis. 28, 2038–2044. 10.1016/j.jstrokecerebrovasdis.2019.02.023 30878372

[B15] Howard G. Goff D. C. (2012). Population Shifts and the Future of Stroke: Forecasts of the Future burden of Stroke. Ann. N. Y Acad. Sci. 1268, 14–20. 10.1111/j.1749-6632.2012.06665.x 22994216 PMC3727892

[B16] Hu R. Cao Q. Sun Z. Chen J. Zheng Q. Xiao F. (2018). A Novel Method of Neural Differentiation of PC12 Cells by Using Opti-MEM as a Basic Induction Medium. Int. J. Mol. Med. 41, 195–201. 10.3892/ijmm.2017.3195 29115371 PMC5746309

[B17] Huang H. Zhong R. Xia Z. Song J. Feng L. (2014). Neuroprotective Effects of Rhynchophylline against Ischemic Brain Injury via Regulation of the Akt/mTOR and TLRs Signaling Pathways. Molecules 19, 11196–11210. 10.3390/molecules190811196 25079660 PMC6270871

[B18] Huang Q. Lan T. Lu J. Zhang H. Zhang D. Lou T. (2018). DiDang Tang Inhibits Endoplasmic Reticulum Stress-Mediated Apoptosis Induced by Oxygen Glucose Deprivation and Intracerebral Hemorrhage through Blockade of the GRP78-IRE1/PERK Pathways. Front. Pharmacol. 9, 1423. 10.3389/fphar.2018.01423 30564125 PMC6288198

[B19] Jiménez-Almonte J. H. King J. D. Luo T. D. Aneja A. Moghadamian E. (2019). Classifications in Brief: Sanders Classification of Intraarticular Fractures of the Calcaneus. Clin. Orthop. Relat. Res. 477, 467–471. 10.1097/CORR.0000000000000539 30664605 PMC6370083

[B20] Jin W. Xu X. Chen X. Qi W. Lu J. Yan X. (2019). Protective Effect of Pig Brain Polypeptides against Corticosterone-Induced Oxidative Stress, Inflammatory Response, and Apoptosis in PC12 Cells. Biomed. Pharmacother. 115, 108890. 10.1016/j.biopha.2019.108890 31022597

[B21] Le K. Song Z. Deng J. Peng X. Zhang J. Wang L. (2020). Quercetin Alleviates Neonatal Hypoxic-Ischemic Brain Injury by Inhibiting Microglia-Derived Oxidative Stress and TLR4-Mediated Inflammation. Inflamm. Res. 69, 1201–1213. 10.1007/s00011-020-01402-5 32944799

[B22] Li J. Zhang K. Zhang Q. Zhou X. Wen L. Ma J. (2020a). PPAR-γ Mediates Ta-VNS-Induced Angiogenesis and Subsequent Functional Recovery after Experimental Stroke in Rats. Biomed. Res. Int. 2020, 8163789. 10.1155/2020/8163789 32775443 PMC7396041

[B23] Li X. Liao Y. Dong Y. Li S. Wang F. Wu R. (2020b). Mib2 Deficiency Inhibits Microglial Activation and Alleviates Ischemia-Induced Brain Injury. Aging Dis. 11, 523–535. 10.14336/AD.2019.0807 32489699 PMC7220279

[B24] Li X. Lin B. Lin Z. Ma Y. Wang Q. Zheng Y. (2021). Exploration in the Mechanism of Fucosterol for the Treatment of Non-small Cell Lung Cancer Based on Network Pharmacology and Molecular Docking. Sci. Rep. 11, 4901. 10.1038/s41598-021-84380-w 33649481 PMC7921686

[B25] Liu J. Wang Y. Akamatsu Y. Lee C. C. Stetler R. A. Lawton M. T. (2014). Vascular Remodeling after Ischemic Stroke: Mechanisms and Therapeutic Potentials. Prog. Neurobiol. 115, 138–156. 10.1016/j.pneurobio.2013.11.004 24291532 PMC4295834

[B26] Lu J. Huang Q. Zhang D. Lan T. Zhang Y. Tang X. (2020). The Protective Effect of DiDang Tang against AlCl3-Induced Oxidative Stress and Apoptosis in PC12 Cells through the Activation of SIRT1-Mediated Akt/Nrf2/HO-1 Pathway. Front. Pharmacol. 11, 466. 10.3389/fphar.2020.00466 32372957 PMC7179660

[B27] Machado M. M. F. Bassani T. B. Cóppola-Segovia V. Moura E. L. R. Zanata S. M. Andreatini R. (2019). PPAR-γ Agonist Pioglitazone Reduces Microglial Proliferation and NF-κB Activation in the Substantia Nigra in the 6-hydroxydopamine Model of Parkinson's Disease. Pharmacol. Rep. 71, 556–564. 10.1016/j.pharep.2018.11.005 31132685

[B28] Mehta V. Parashar A. Udayabanu M. (2017). Quercetin Prevents Chronic Unpredictable Stress Induced Behavioral Dysfunction in Mice by Alleviating Hippocampal Oxidative and Inflammatory Stress. Physiol. Behav. 171, 69–78. 10.1016/j.physbeh.2017.01.006 28069457

[B29] Park D. J. Shah F. A. Koh P. O. (2018). Quercetin Attenuates Neuronal Cells Damage in a Middle Cerebral Artery Occlusion Animal Model. J. Vet. Med. Sci. 80, 676–683. 10.1292/jvms.17-0693 29563391 PMC5938200

[B38] Peng Z. Wang S. Chen G. (2018). Gastrodin Alleviates Cerebral Ischemic Damage in Mice by Improving Anti-oxidant and Anti-inflammation Activities and Inhibiting Apoptosis Pathway. Neurochem. Res. 40 (4), 661–673. 10.1007/s11064-015-1513-5 25582916

[B30] Qi W. Xu X. Wang M. Li X. Wang C. Sun L. (2019). Inhibition of Wee1 Sensitizes AML Cells to ATR Inhibitor VE-822-Induced DNA Damage and Apoptosis. Biochem. Pharmacol. 164, 273–282. 10.1016/j.bcp.2019.04.022 31014753

[B32] Seo H. B. Kang B. K. Kim J. H. Choi Y. W. Hong J. W. Choi B. T. (2015). Partially Purified Components of Uncaria Sinensis Attenuate Blood Brain Barrier Disruption after Ischemic Brain Injury in Mice. BMC Complement. Altern. Med. 15, 157. 10.1186/s12906-015-0678-4 26012470 PMC4443505

[B33] Shen Y. C. Lu C. K. Liou K. T. Hou Y. C. Lin Y. L. Wang Y. H. (2015). Common and Unique Mechanisms of Chinese Herbal Remedies on Ischemic Stroke Mice Revealed by Transcriptome Analyses. J. Ethnopharmacol. 173, 370–382. 10.1016/j.jep.2015.07.018 26239152

[B34] Song M. T. Ruan J. Zhang R. Y. Deng J. Ma Z. Q. Ma S. P. (2018). Astragaloside IV Ameliorates Neuroinflammation-Induced Depressive-like Behaviors in Mice via the PPARγ/NF-κB/NLRP3 Inflammasome axis. Acta Pharmacol. Sin 39, 1559–1570. 10.1038/aps.2017.208 29795356 PMC6289360

[B35] Su H. Zhang H. Wei X. Pan D. Jing L. Zhao D. (2018). Comparative Proteomic Analysis of *Rana chensinensis* Oviduct. Molecules 23. 10.3390/molecules23061384 PMC6099995 29890619

[B36] Takeda H. Yamaguchi T. Yano H. Tanaka J. (2021). Microglial Metabolic Disturbances and Neuroinflammation in Cerebral Infarction. J. Pharmacol. Sci. 145, 130–139. 10.1016/j.jphs.2020.11.007 33357771

[B37] Wang L. Li Z. Zhao X. Liu W. Liu Y. Yang J. (2013). A Network Study of Chinese Medicine Xuesaitong Injection to Elucidate a Complex Mode of Action with Multicompound, Multitarget, and Multipathway. Evid. Based Complement. Alternat Med. 2013, 652373. 10.1155/2013/652373 24058375 PMC3766588

[B39] Wang Y. Xiao G. He S. Liu X. Zhu L. Yang X. (2020a). Protection against Acute Cerebral Ischemia/reperfusion Injury by QiShenYiQi via Neuroinflammatory Network Mobilization. Biomed. Pharmacother. 125, 109945. 10.1016/j.biopha.2020.109945 32028240

[B40] Wang Y. Y. Chang C. Y. Lin S. Y. Wang J. D. Wu C. C. Chen W. Y. (2020b). Quercetin Protects against Cerebral Ischemia/reperfusion and Oxygen Glucose Deprivation/reoxygenation Neurotoxicity. J. Nutr. Biochem. 83, 108436. 10.1016/j.jnutbio.2020.108436 32599520

[B49] Wang M. Zhong B. Li M. (2021). Identification of Potential Core Genes and Pathways Predicting Pathogenesis in Head and Neck Squamous Cell Carcinoma. Biosci. Rep. 41. 10.1042/BSR20204148 PMC8164109 33982750

[B41] Wu X. J. Sun X. H. Wang S. W. Chen J. L. Bi Y. H. Jiang D. X. (2018). Mifepristone Alleviates Cerebral Ischemia-Reperfusion Injury in Rats by Stimulating PPAR γ. Eur. Rev. Med. Pharmacol. Sci. 22, 5688–5696. 10.26355/eurrev_201809_15836 30229846

[B42] Xu H. Qin W. Hu X. Mu S. Zhu J. Lu W. (2018). Lentivirus-mediated Overexpression of OTULIN Ameliorates Microglia Activation and Neuroinflammation by Depressing the Activation of the NF-κB Signaling Pathway in Cerebral Ischemia/reperfusion Rats. J. Neuroinflamm. 15, 83. 10.1186/s12974-018-1117-5 PMC5856386 29544517

[B43] Xu H. H. Li S. M. Xu R. Fang L. Xu H. Tong P. J. (2020). Predication of the Underlying Mechanism of Bushenhuoxue Formula Acting on Knee Osteoarthritis via Network Pharmacology-Based Analyses Combined with Experimental Validation. J. Ethnopharmacol. 263, 113217. 10.1016/j.jep.2020.113217 32763417

[B44] Xu C. Li R. Wu J. (2021). Effects of Yuanhu- Zhitong Tablets on Alcohol-Induced Conditioned Place Preference in Mice. Biomed. Pharmacother. 133, 110962. 10.1016/j.biopha.2020.110962 33166765

[B45] Yang M. Wang Y. Fan Z. Xue Q. Njateng G. S. S. Liu Y. (2021). Chemical Constituents and Anti-inflammatory Activity of the Total Alkaloid Extract from Melodinus Cochinchinensis (Lour.) Merr. And its Inhibition of the NF-κB and MAPK Signaling Pathways. Phytomedicine 91, 153684. 10.1016/j.phymed.2021.153684 34400050

[B46] Ye Z. Hu J. Xu H. Sun B. Jin Y. Zhang Y. (2021). Serum Exosomal microRNA-27-3p Aggravates Cerebral Injury and Inflammation in Patients with Acute Cerebral Infarction by Targeting PPARγ. Inflammation 44, 1035–1048. 10.1007/s10753-020-01399-3 33394189

[B47] Yu S. S. Zhao J. Zheng W. P. Zhao Y. (2010). Neuroprotective Effect of 4-hydroxybenzyl Alcohol against Transient Focal Cerebral Ischemia via Anti-apoptosis in Rats. Brain Res. 1308, 167–175. 10.1016/j.brainres.2009.10.037 19857470

[B48] Yuan C. Wang M. H. Wang F. Chen P. Y. Ke X. G. Yu B. (2021). Network Pharmacology and Molecular Docking Reveal the Mechanism of Scopoletin against Non-small Cell Lung Cancer. Life Sci. 270, 119105. 10.1016/j.lfs.2021.119105 33497736

