# Supplementary material for: Underlying Mechanism and Active Ingredients of Tianma Gouteng Acting on Cerebral Infarction as Determined via Network Pharmacology Analysis Combined With Experimental Validation
Source: Front Pharmacol. 2021 Nov 16;12:760503. doi: 10.3389/fphar.2021.760503 (PMC8635202; doi:10.3389/fphar.2021.760503)
Supplement: Supplementary file 1 [file Table1.docx]

**Supplementary Table S1** HPLC gradient elution process

| Time(min) | Mobile phase A(%) | Mobile phase B(%) |
| --- | --- | --- |
| 0-5 | 2 | 98 |
| 6-10 | 8 | 92 |
| 11-18 | 13 | 87 |
| 19-24 | 21 | 79 |
| 25-28 | 30 | 70 |
| 29-32 | 35 | 65 |
| 33-40 | 55 | 45 |
