# Supplementary material for: Underlying Mechanism and Active Ingredients of Tianma Gouteng Acting on Cerebral Infarction as Determined via Network Pharmacology Analysis Combined With Experimental Validation
Source: Front Pharmacol. 2021 Nov 16;12:760503. doi: 10.3389/fphar.2021.760503 (PMC8635202; doi:10.3389/fphar.2021.760503)
Supplement: Supplementary file 2 [file DataSheet1.zip › original data/GO&KEGG/KEGG pathway.htm]

out


out Pathway Enrichment

| # | Pathway | out (31) | All (8514) | Pvalue | Qvalue | Pathway ID |
| 1 | Neuroactive ligand-receptor interaction | 10 | 293 | 0.000000 | 0.000008 | ko04080 |
| 2 | Serotonergic synapse | 7 | 125 | 0.000000 | 0.000021 | ko04726 |
| 3 | Pathways in cancer | 11 | 550 | 0.000002 | 0.000108 | ko05200 |
| 4 | African trypanosomiasis | 4 | 42 | 0.000015 | 0.000618 | ko05143 |
| 5 | HIF-1 signaling pathway | 5 | 102 | 0.000030 | 0.000863 | ko04066 |
| 6 | IL-17 signaling pathway | 5 | 106 | 0.000036 | 0.000863 | ko04657 |
| 7 | Amoebiasis | 5 | 106 | 0.000036 | 0.000863 | ko05146 |
| 8 | AGE-RAGE signaling pathway in diabetic complications | 5 | 114 | 0.000051 | 0.001071 | ko04933 |
| 9 | Cholinergic synapse | 5 | 121 | 0.000067 | 0.001267 | ko04725 |
| 10 | Thyroid hormone signaling pathway | 5 | 131 | 0.000098 | 0.001664 | ko04919 |
| 11 | Th17 cell differentiation | 5 | 186 | 0.000505 | 0.007651 | ko04659 |
| 12 | Calcium signaling pathway | 5 | 189 | 0.000543 | 0.007651 | ko04020 |
| 13 | Human cytomegalovirus infection | 6 | 307 | 0.000719 | 0.008730 | ko05163 |
| 14 | Endocrine and other factor-regulated calcium reabsorption | 3 | 50 | 0.000763 | 0.008730 | ko04961 |
| 15 | Chagas disease (American trypanosomiasis) | 4 | 116 | 0.000775 | 0.008730 | ko05142 |
| 16 | Proteoglycans in cancer | 5 | 210 | 0.000877 | 0.009259 | ko05205 |
| 17 | Regulation of lipolysis in adipocyte | 3 | 56 | 0.001063 | 0.010211 | ko04923 |
| 18 | NF-kappa B signaling pathway | 4 | 127 | 0.001088 | 0.010211 | ko04064 |
| 19 | TNF signaling pathway | 4 | 130 | 0.001186 | 0.010552 | ko04668 |
| 20 | VEGF signaling pathway | 3 | 61 | 0.001364 | 0.011523 | ko04370 |
| 21 | Dopaminergic synapse | 4 | 142 | 0.001645 | 0.013235 | ko04728 |
| 22 | Fluid shear stress and atherosclerosis | 4 | 149 | 0.001963 | 0.014907 | ko05418 |
| 23 | Arachidonic acid metabolism | 3 | 70 | 0.002029 | 0.014907 | ko00590 |
| 24 | Leishmaniasis | 4 | 155 | 0.002267 | 0.015965 | ko05140 |
| 25 | Non-small cell lung cancer | 3 | 74 | 0.002379 | 0.016082 | ko05223 |
| 26 | Hepatitis B | 4 | 160 | 0.002545 | 0.016542 | ko05161 |
| 27 | Gastric acid secretion | 3 | 77 | 0.002665 | 0.016680 | ko04971 |
| 28 | MicroRNAs in cancer | 4 | 168 | 0.003037 | 0.018329 | ko05206 |
| 29 | Insulin secretion | 3 | 90 | 0.004145 | 0.024157 | ko04911 |
| 30 | Gap junction | 3 | 98 | 0.005262 | 0.027616 | ko04540 |
| 31 | Salivary secretion | 3 | 100 | 0.005567 | 0.027616 | ko04970 |
| 32 | Small cell lung cancer | 3 | 100 | 0.005567 | 0.027616 | ko05222 |
| 33 | Inflammatory mediator regulation of TRP channels | 3 | 101 | 0.005724 | 0.027616 | ko04750 |
| 34 | Morphine addiction | 3 | 101 | 0.005724 | 0.027616 | ko05032 |
| 35 | Prostate cancer | 3 | 101 | 0.005724 | 0.027616 | ko05215 |
| 36 | Pancreatic secretion | 3 | 102 | 0.005883 | 0.027616 | ko04972 |
| 37 | Choline metabolism in cancer | 3 | 109 | 0.007070 | 0.032295 | ko05231 |
| 38 | Aldosterone-regulated sodium reabsorption | 2 | 37 | 0.007894 | 0.035107 | ko04960 |
| 39 | C-type lectin receptor signaling pathway | 3 | 116 | 0.008391 | 0.035412 | ko04625 |
| 40 | Leukocyte transendothelial migration | 3 | 117 | 0.008591 | 0.035412 | ko04670 |
| 41 | Parathyroid hormone synthesis, secretion and action | 3 | 117 | 0.008591 | 0.035412 | ko04928 |
| 42 | Antifolate resistance | 2 | 40 | 0.009183 | 0.036950 | ko01523 |
| 43 | PI3K-Akt signaling pathway | 5 | 374 | 0.010536 | 0.041407 | ko04151 |
| 44 | Relaxin signaling pathway | 3 | 138 | 0.013444 | 0.051638 | ko04926 |
| 45 | Ovarian Steroidogenesis | 2 | 51 | 0.014643 | 0.054991 | ko04913 |
| 46 | Malaria | 2 | 55 | 0.016902 | 0.062095 | ko05144 |
| 47 | Influenza A | 4 | 277 | 0.017276 | 0.062121 | ko05164 |
| 48 | Measles | 3 | 155 | 0.018320 | 0.064500 | ko05162 |
| 49 | Oxytocin signaling pathway | 3 | 158 | 0.019270 | 0.066461 | ko04921 |
| 50 | Long-term depression | 2 | 60 | 0.019920 | 0.067330 | ko04730 |
| 51 | Retrograde endocannabinoid signaling | 3 | 168 | 0.022633 | 0.074999 | ko04723 |
| 52 | Estrogen signaling pathway | 3 | 171 | 0.023701 | 0.075308 | ko04915 |
| 53 | cGMP - PKG signaling pathway | 3 | 172 | 0.024063 | 0.075308 | ko04022 |
| 54 | mTOR signaling pathway | 3 | 172 | 0.024063 | 0.075308 | ko04150 |
| 55 | Acute myeloid leukemia | 2 | 68 | 0.025181 | 0.077374 | ko05221 |
| 56 | Long-term potentiation | 2 | 70 | 0.026576 | 0.080202 | ko04720 |
| 57 | Cytosolic DNA-sensing pathway | 2 | 72 | 0.028002 | 0.082311 | ko04623 |
| 58 | Amphetamine addiction | 2 | 73 | 0.028726 | 0.082311 | ko05031 |
| 59 | B cell receptor signaling pathway | 2 | 74 | 0.029458 | 0.082311 | ko04662 |
| 60 | Osteoclast differentiation | 3 | 187 | 0.029857 | 0.082311 | ko04380 |
| 61 | Bile secretion | 2 | 75 | 0.030197 | 0.082311 | ko04976 |
| 62 | Glioma | 2 | 75 | 0.030197 | 0.082311 | ko05214 |
| 63 | MAPK signaling pathway | 4 | 332 | 0.031144 | 0.083545 | ko04010 |
| 64 | Transcriptional misregulation in cancers | 3 | 195 | 0.033225 | 0.085084 | ko05202 |
| 65 | PPAR signaling pathway | 2 | 79 | 0.033228 | 0.085084 | ko03320 |
| 66 | Fc epsilon RI signaling pathway | 2 | 79 | 0.033228 | 0.085084 | ko04664 |
| 67 | EGFR tyrosine kinase inhibitor resistance | 2 | 82 | 0.035577 | 0.088419 | ko01521 |
| 68 | Thyroid hormone synthesis | 2 | 82 | 0.035577 | 0.088419 | ko04918 |
| 69 | Drug metabolism - other enzymes | 2 | 84 | 0.037178 | 0.091058 | ko00983 |
| 70 | Toxoplasmosis | 3 | 207 | 0.038638 | 0.091747 | ko05145 |
| 71 | cAMP signaling pathway | 3 | 208 | 0.039108 | 0.091747 | ko04024 |
| 72 | ErbB signaling pathway | 2 | 87 | 0.039631 | 0.091747 | ko04012 |
| 73 | Adipocytokine signaling pathway | 2 | 87 | 0.039631 | 0.091747 | ko04920 |
| 74 | Rap1 signaling pathway | 3 | 211 | 0.040537 | 0.092431 | ko04015 |
| 75 | Focal adhesion | 3 | 212 | 0.041019 | 0.092431 | ko04510 |
| 76 | Chemical carcinogenesis | 2 | 90 | 0.042144 | 0.093715 | ko05204 |
| 77 | Fc gamma R-mediated phagocytosis | 2 | 95 | 0.046463 | 0.101977 | ko04666 |
| 78 | GnRH signaling pathway | 2 | 96 | 0.047346 | 0.102583 | ko04912 |
| 79 | Salmonella infection | 2 | 97 | 0.048235 | 0.103187 | ko05132 |
| 80 | Circadian entrainment | 2 | 99 | 0.050032 | 0.104387 | ko04713 |
| 81 | GABAergic synapse | 2 | 99 | 0.050032 | 0.104387 | ko04727 |
| 82 | Phosphatidylinositol signaling system | 2 | 105 | 0.055565 | 0.112909 | ko04070 |
| 83 | Melanogenesis | 2 | 105 | 0.055565 | 0.112909 | ko04916 |
| 84 | Kaposi sarcoma-associated herpesvirus infection | 3 | 241 | 0.056270 | 0.112909 | ko05167 |
| 85 | Ras signaling pathway | 3 | 243 | 0.057411 | 0.112909 | ko04014 |
| 86 | Aldosterone synthesis and secretion | 2 | 107 | 0.057456 | 0.112909 | ko04925 |
| 87 | T cell receptor signaling pathway | 2 | 114 | 0.064250 | 0.124808 | ko04660 |
| 88 | Endocrine resistance | 2 | 116 | 0.066240 | 0.127211 | ko01522 |
| 89 | Taste transduction | 2 | 117 | 0.067243 | 0.127685 | ko04742 |
| 90 | Glutamatergic synapse | 2 | 118 | 0.068250 | 0.128159 | ko04724 |
| 91 | Toll-like receptor signaling pathway | 2 | 122 | 0.072332 | 0.134331 | ko04620 |
| 92 | Vascular smooth muscle contraction | 2 | 125 | 0.075445 | 0.138589 | ko04270 |
| 93 | Sphingolipid signaling pathway | 2 | 132 | 0.082873 | 0.150597 | ko04071 |
| 94 | Platelet activation | 2 | 135 | 0.086125 | 0.154841 | ko04611 |
| 95 | Hepatitis C | 2 | 144 | 0.096108 | 0.170970 | ko05160 |
| 96 | Inflammatiory bowel disease (IBD) | 2 | 145 | 0.097237 | 0.171178 | ko05321 |
| 97 | Linoleic acid metabolism | 1 | 30 | 0.103824 | 0.180889 | ko00591 |
| 98 | Wnt signaling pathway | 2 | 159 | 0.113442 | 0.195629 | ko04310 |
| 99 | Type I diabetes mellitus | 2 | 166 | 0.121797 | 0.207916 | ko04940 |
| 100 | Th1 and Th2 cell differentiation | 2 | 168 | 0.124212 | 0.209919 | ko04658 |
| 101 | Herpes simplex infection | 3 | 341 | 0.125805 | 0.210506 | ko05168 |
| 102 | Necroptosis | 2 | 176 | 0.133992 | 0.219644 | ko04217 |
| 103 | Prion diseases | 1 | 40 | 0.136053 | 0.219644 | ko05020 |
| 104 | Non-alcoholic fatty liver disease (NAFLD) | 2 | 178 | 0.136465 | 0.219644 | ko04932 |
| 105 | Hepatocellular carcinoma | 2 | 178 | 0.136465 | 0.219644 | ko05225 |
| 106 | Thyroid cancer | 1 | 43 | 0.145501 | 0.229811 | ko05216 |
| 107 | Bladder cancer | 1 | 43 | 0.145501 | 0.229811 | ko05219 |
| 108 | Rheumatoid arthritis | 2 | 188 | 0.148984 | 0.233133 | ko05323 |
| 109 | NOD-like receptor signaling pathway | 2 | 193 | 0.155333 | 0.240838 | ko04621 |
| 110 | Alzheimer disease | 2 | 199 | 0.163023 | 0.249843 | ko05010 |
| 111 | Carbohydrate digestion and absorption | 1 | 49 | 0.164098 | 0.249843 | ko04973 |
| 112 | Cholesterol metabolism | 1 | 51 | 0.170210 | 0.256835 | ko04979 |
| 113 | Chemokine signaling pathway | 2 | 207 | 0.173387 | 0.259314 | ko04062 |
| 114 | Cocaine addiction | 1 | 53 | 0.176278 | 0.261325 | ko05030 |
| 115 | Regulation of actin cytoskeleton | 2 | 218 | 0.187818 | 0.276011 | ko04810 |
| 116 | Human papillomavirus infection | 3 | 416 | 0.191425 | 0.278887 | ko05165 |
| 117 | Vibrio cholerae infection | 1 | 59 | 0.194226 | 0.280549 | ko05110 |
| 118 | Pathogenic Escherichia coli infection | 1 | 63 | 0.205981 | 0.295007 | ko05130 |
| 119 | Proteasome | 1 | 64 | 0.208894 | 0.296664 | ko03050 |
| 120 | Shigellosis | 1 | 66 | 0.214688 | 0.302352 | ko05131 |
| 121 | Central carbon metabolism in cancer | 1 | 69 | 0.223302 | 0.311885 | ko05230 |
| 122 | Glycerolipid metabolism | 1 | 71 | 0.228994 | 0.314635 | ko00561 |
| 123 | Renal cell carcinoma | 1 | 71 | 0.228994 | 0.314635 | ko05211 |
| 124 | Steroid hormone biosynthesis | 1 | 72 | 0.231825 | 0.314724 | ko00140 |
| 125 | p53 signaling pathway | 1 | 73 | 0.234646 | 0.314724 | ko04115 |
| 126 | Mitophagy - animal | 1 | 73 | 0.234646 | 0.314724 | ko04137 |
| 127 | Prolactin signaling pathway | 1 | 74 | 0.237457 | 0.315986 | ko04917 |
| 128 | Retinol metabolism | 1 | 75 | 0.240258 | 0.317016 | ko00830 |
| 129 | Tuberculosis | 2 | 259 | 0.242880 | 0.317016 | ko05152 |
| 130 | Legionellosis | 1 | 77 | 0.245830 | 0.317016 | ko05134 |
| 131 | Pancreatic cancer | 1 | 77 | 0.245830 | 0.317016 | ko05212 |
| 132 | Epithelial cell signaling in Helicobacter pylori infection | 1 | 78 | 0.248601 | 0.317016 | ko05120 |
| 133 | RIG-I-like receptor signaling pathway | 1 | 79 | 0.251362 | 0.317016 | ko04622 |
| 134 | Chronic myeloid leukemia | 1 | 79 | 0.251362 | 0.317016 | ko05220 |
| 135 | Drug metabolism - cytochrome P450 | 1 | 83 | 0.262309 | 0.328372 | ko00982 |
| 136 | Metabolism of xenobiotics by cytochrome P450 | 1 | 87 | 0.273101 | 0.339368 | ko00980 |
| 137 | TGF-beta signaling pathway | 1 | 92 | 0.286376 | 0.350707 | ko04350 |
| 138 | Pertussis | 1 | 92 | 0.286376 | 0.350707 | ko05133 |
| 139 | Natural killer cell mediated cytotoxicity | 3 | 523 | 0.296426 | 0.360403 | ko04650 |
| 140 | Complement and coagulation cascades | 1 | 97 | 0.299416 | 0.361438 | ko04610 |
| 141 | ECM-receptor interaction | 1 | 98 | 0.301996 | 0.361967 | ko04512 |
| 142 | Protein digestion and absorption | 1 | 101 | 0.309682 | 0.367570 | ko04974 |
| 143 | Epstein-Barr virus infection | 2 | 309 | 0.311021 | 0.367570 | ko05169 |
| 144 | Graft-versus-host disease | 2 | 314 | 0.317812 | 0.372988 | ko05332 |
| 145 | Cytokine-cytokine receptor interaction | 2 | 330 | 0.339456 | 0.395642 | ko04060 |
| 146 | Glycerophospholipid metabolism | 1 | 117 | 0.349309 | 0.404338 | ko00564 |
| 147 | Insulin resistance | 1 | 122 | 0.361235 | 0.415298 | ko04931 |
| 148 | Oocyte meiosis | 1 | 131 | 0.382170 | 0.436397 | ko04114 |
| 149 | Autophagy - animal | 1 | 133 | 0.386731 | 0.438642 | ko04140 |
| 150 | FoxO signaling pathway | 1 | 139 | 0.400220 | 0.450915 | ko04068 |
| 151 | Apelin signaling pathway | 1 | 141 | 0.404653 | 0.452889 | ko04371 |
| 152 | Apoptosis | 1 | 153 | 0.430588 | 0.474845 | ko04210 |
| 153 | Adrenergic signaling in cardiomyocytes | 1 | 154 | 0.432699 | 0.474845 | ko04261 |
| 154 | Allograft rejection | 1 | 154 | 0.432699 | 0.474845 | ko05330 |
| 155 | Phospholipase D signaling pathway | 1 | 157 | 0.438987 | 0.477577 | ko04072 |
| 156 | Hippo signaling pathway | 1 | 159 | 0.443142 | 0.477577 | ko04390 |
| 157 | Parkinson disease | 1 | 161 | 0.447267 | 0.477577 | ko05012 |
| 158 | Gastric cancer | 1 | 161 | 0.447267 | 0.477577 | ko05226 |
| 159 | Breast cancer | 1 | 162 | 0.449318 | 0.477577 | ko05224 |
| 160 | Jak-STAT signaling pathway | 1 | 167 | 0.459465 | 0.485310 | ko04630 |
| 161 | Hematopoietic cell lineage | 1 | 176 | 0.477274 | 0.500990 | ko04640 |
| 162 | Axon guidance | 1 | 179 | 0.483084 | 0.503958 | ko04360 |
| 163 | Alcoholism | 1 | 189 | 0.502001 | 0.520479 | ko05034 |
| 164 | Systemic lupus erythematosus | 1 | 226 | 0.566339 | 0.583605 | ko05322 |
| 165 | Phagosome | 1 | 305 | 0.677914 | 0.694349 | ko04145 |
| 166 | Cellular senescence | 1 | 328 | 0.704792 | 0.717529 | ko04218 |
| 167 | HTLV-I infection | 1 | 386 | 0.763287 | 0.772428 | ko05166 |
| 168 | Metabolic pathways | 4 | 1416 | 0.782186 | 0.786842 | ko01100 |
| 169 | Antigen processing and presentation | 1 | 536 | 0.867268 | 0.867268 | ko04612 |

Pathway Detail

| # | Pathway | Pathway ID | K\_id | Differentially expressed genes | K\_id desc |
| 1 | Neuroactive ligand-receptor interaction | ko04080 || K04130 | ENSG00000181072 | CHRM2; muscarinic acetylcholine receptor M2 |
| K04131 | ENSG00000133019 | CHRM3; muscarinic acetylcholine receptor M3 |
| K04138 | ENSG00000150594 | ADRA2A; adrenergic receptor alpha-2A |
| K04139 | ENSG00000274286 | ADRA2B; adrenergic receptor alpha-2B |
| K04140 | ENSG00000184160 | ADRA2C; adrenergic receptor alpha-2C |
| K04145 | ENSG00000149295 | DRD2; dopamine receptor D2 |
| K04147 | ENSG00000069696 | DRD4; dopamine receptor D4 |
| K04214 | ENSG00000082556 | OPRK1; kappa-type opioid receptor |
| K04215 | ENSG00000112038 | OPRM1; mu-type opioid receptor |
| K04260 | ENSG00000050628 | PTGER3; prostaglandin E receptor 3 |

| 2 | Serotonergic synapse | ko04726 || K00461 | ENSG00000012779 | ALOX5; arachidonate 5-lipoxygenase [EC:1.13.11.34] |
| K00509 | ENSG00000095303 | PTGS1, COX1; prostaglandin-endoperoxide synthase 1 [EC:1.14.99.1] |
| K02677 | ENSG00000154229 | PRKCA; classical protein kinase C alpha type [EC:2.7.11.13] |
| K04819 | ENSG00000166736 | HTR3; 5-hydroxytryptamine receptor 3 |
| K05037 | ENSG00000108576 | SLC6A4, SERT; solute carrier family 6 (neurotransmitter transporter, serotonin) member 4 |
| K11987 | ENSG00000073756 | PTGS2, COX2; prostaglandin-endoperoxide synthase 2 [EC:1.14.99.1] |
| K19662 | ENSG00000166501 | PRKCB; classical protein kinase C beta type [EC:2.7.11.13] |

| 3 | Pathways in cancer | ko05200 || K01403 | ENSG00000100985 | MMP9; matrix metalloproteinase-9 (gelatinase B) [EC:3.4.24.35] |
| K02677 | ENSG00000154229 | PRKCA; classical protein kinase C alpha type [EC:2.7.11.13] |
| K04260 | ENSG00000050628 | PTGER3; prostaglandin E receptor 3 |
| K04467 | ENSG00000213341 | IKBKA, IKKA, CHUK; inhibitor of nuclear factor kappa-B kinase subunit alpha [EC:2.7.11.10] |
| K04687 | ENSG00000111537 | IFNG; interferon gamma |
| K08268 | ENSG00000100644 | HIF1A; hypoxia-inducible factor 1 alpha |
| K08524 | ENSG00000186350 | RXRA, NR2B1; retinoid X receptor alpha |
| K08550 | ENSG00000091831 | ESR1, NR3A1; estrogen receptor alpha |
| K08557 | ENSG00000169083 | AR, NR3C4; androgen receptor |
| K11987 | ENSG00000073756 | PTGS2, COX2; prostaglandin-endoperoxide synthase 2 [EC:1.14.99.1] |
| K19662 | ENSG00000166501 | PRKCB; classical protein kinase C beta type [EC:2.7.11.13] |

| 4 | African trypanosomiasis | ko05143 || K02677 | ENSG00000154229 | PRKCA; classical protein kinase C alpha type [EC:2.7.11.13] |
| K04519 | ENSG00000125538 | IL1B; interleukin 1 beta |
| K04687 | ENSG00000111537 | IFNG; interferon gamma |
| K19662 | ENSG00000166501 | PRKCB; classical protein kinase C beta type [EC:2.7.11.13] |

| 5 | HIF-1 signaling pathway | ko04066 || K02677 | ENSG00000154229 | PRKCA; classical protein kinase C alpha type [EC:2.7.11.13] |
| K03982 | ENSG00000106366 | SERPINE1, PAI1; plasminogen activator inhibitor 1 |
| K04687 | ENSG00000111537 | IFNG; interferon gamma |
| K08268 | ENSG00000100644 | HIF1A; hypoxia-inducible factor 1 alpha |
| K19662 | ENSG00000166501 | PRKCB; classical protein kinase C beta type [EC:2.7.11.13] |

| 6 | IL-17 signaling pathway | ko04657 || K01403 | ENSG00000100985 | MMP9; matrix metalloproteinase-9 (gelatinase B) [EC:3.4.24.35] |
| K04467 | ENSG00000213341 | IKBKA, IKKA, CHUK; inhibitor of nuclear factor kappa-B kinase subunit alpha [EC:2.7.11.10] |
| K04519 | ENSG00000125538 | IL1B; interleukin 1 beta |
| K04687 | ENSG00000111537 | IFNG; interferon gamma |
| K11987 | ENSG00000073756 | PTGS2, COX2; prostaglandin-endoperoxide synthase 2 [EC:1.14.99.1] |

| 7 | Amoebiasis | ko05146 || K02677 | ENSG00000154229 | PRKCA; classical protein kinase C alpha type [EC:2.7.11.13] |
| K04519 | ENSG00000125538 | IL1B; interleukin 1 beta |
| K04687 | ENSG00000111537 | IFNG; interferon gamma |
| K06236 | ENSG00000108821 | COL1A; collagen, type I, alpha |
| K19662 | ENSG00000166501 | PRKCB; classical protein kinase C beta type [EC:2.7.11.13] |

| 8 | AGE-RAGE signaling pathway in diabetic complications | ko04933 || K02677 | ENSG00000154229 | PRKCA; classical protein kinase C alpha type [EC:2.7.11.13] |
| K03982 | ENSG00000106366 | SERPINE1, PAI1; plasminogen activator inhibitor 1 |
| K04519 | ENSG00000125538 | IL1B; interleukin 1 beta |
| K06236 | ENSG00000108821 | COL1A; collagen, type I, alpha |
| K19662 | ENSG00000166501 | PRKCB; classical protein kinase C beta type [EC:2.7.11.13] |

| 9 | Cholinergic synapse | ko04725 || K01049 | ENSG00000087085 | ACHE; acetylcholinesterase [EC:3.1.1.7] |
| K02677 | ENSG00000154229 | PRKCA; classical protein kinase C alpha type [EC:2.7.11.13] |
| K04130 | ENSG00000181072 | CHRM2; muscarinic acetylcholine receptor M2 |
| K04131 | ENSG00000133019 | CHRM3; muscarinic acetylcholine receptor M3 |
| K19662 | ENSG00000166501 | PRKCB; classical protein kinase C beta type [EC:2.7.11.13] |

| 10 | Thyroid hormone signaling pathway | ko04919 || K02677 | ENSG00000154229 | PRKCA; classical protein kinase C alpha type [EC:2.7.11.13] |
| K08268 | ENSG00000100644 | HIF1A; hypoxia-inducible factor 1 alpha |
| K08524 | ENSG00000186350 | RXRA, NR2B1; retinoid X receptor alpha |
| K08550 | ENSG00000091831 | ESR1, NR3A1; estrogen receptor alpha |
| K19662 | ENSG00000166501 | PRKCB; classical protein kinase C beta type [EC:2.7.11.13] |

| 11 | Th17 cell differentiation | ko04659 || K04467 | ENSG00000213341 | IKBKA, IKKA, CHUK; inhibitor of nuclear factor kappa-B kinase subunit alpha [EC:2.7.11.10] |
| K04519 | ENSG00000125538 | IL1B; interleukin 1 beta |
| K04687 | ENSG00000111537 | IFNG; interferon gamma |
| K08268 | ENSG00000100644 | HIF1A; hypoxia-inducible factor 1 alpha |
| K08524 | ENSG00000186350 | RXRA, NR2B1; retinoid X receptor alpha |

| 12 | Calcium signaling pathway | ko04020 || K02677 | ENSG00000154229 | PRKCA; classical protein kinase C alpha type [EC:2.7.11.13] |
| K04130 | ENSG00000181072 | CHRM2; muscarinic acetylcholine receptor M2 |
| K04131 | ENSG00000133019 | CHRM3; muscarinic acetylcholine receptor M3 |
| K04260 | ENSG00000050628 | PTGER3; prostaglandin E receptor 3 |
| K19662 | ENSG00000166501 | PRKCB; classical protein kinase C beta type [EC:2.7.11.13] |

| 13 | Human cytomegalovirus infection | ko05163 || K02677 | ENSG00000154229 | PRKCA; classical protein kinase C alpha type [EC:2.7.11.13] |
| K04260 | ENSG00000050628 | PTGER3; prostaglandin E receptor 3 |
| K04467 | ENSG00000213341 | IKBKA, IKKA, CHUK; inhibitor of nuclear factor kappa-B kinase subunit alpha [EC:2.7.11.10] |
| K04519 | ENSG00000125538 | IL1B; interleukin 1 beta |
| K11987 | ENSG00000073756 | PTGS2, COX2; prostaglandin-endoperoxide synthase 2 [EC:1.14.99.1] |
| K19662 | ENSG00000166501 | PRKCB; classical protein kinase C beta type [EC:2.7.11.13] |

| 14 | Endocrine and other factor-regulated calcium reabsorption | ko04961 || K02677 | ENSG00000154229 | PRKCA; classical protein kinase C alpha type [EC:2.7.11.13] |
| K08550 | ENSG00000091831 | ESR1, NR3A1; estrogen receptor alpha |
| K19662 | ENSG00000166501 | PRKCB; classical protein kinase C beta type [EC:2.7.11.13] |

| 15 | Chagas disease (American trypanosomiasis) | ko05142 || K03982 | ENSG00000106366 | SERPINE1, PAI1; plasminogen activator inhibitor 1 |
| K04467 | ENSG00000213341 | IKBKA, IKKA, CHUK; inhibitor of nuclear factor kappa-B kinase subunit alpha [EC:2.7.11.10] |
| K04519 | ENSG00000125538 | IL1B; interleukin 1 beta |
| K04687 | ENSG00000111537 | IFNG; interferon gamma |

| 16 | Proteoglycans in cancer | ko05205 || K01403 | ENSG00000100985 | MMP9; matrix metalloproteinase-9 (gelatinase B) [EC:3.4.24.35] |
| K02677 | ENSG00000154229 | PRKCA; classical protein kinase C alpha type [EC:2.7.11.13] |
| K08268 | ENSG00000100644 | HIF1A; hypoxia-inducible factor 1 alpha |
| K08550 | ENSG00000091831 | ESR1, NR3A1; estrogen receptor alpha |
| K19662 | ENSG00000166501 | PRKCB; classical protein kinase C beta type [EC:2.7.11.13] |

| 17 | Regulation of lipolysis in adipocyte | ko04923 || K00509 | ENSG00000095303 | PTGS1, COX1; prostaglandin-endoperoxide synthase 1 [EC:1.14.99.1] |
| K04260 | ENSG00000050628 | PTGER3; prostaglandin E receptor 3 |
| K11987 | ENSG00000073756 | PTGS2, COX2; prostaglandin-endoperoxide synthase 2 [EC:1.14.99.1] |

| 18 | NF-kappa B signaling pathway | ko04064 || K04467 | ENSG00000213341 | IKBKA, IKKA, CHUK; inhibitor of nuclear factor kappa-B kinase subunit alpha [EC:2.7.11.10] |
| K04519 | ENSG00000125538 | IL1B; interleukin 1 beta |
| K11987 | ENSG00000073756 | PTGS2, COX2; prostaglandin-endoperoxide synthase 2 [EC:1.14.99.1] |
| K19662 | ENSG00000166501 | PRKCB; classical protein kinase C beta type [EC:2.7.11.13] |

| 19 | TNF signaling pathway | ko04668 || K01403 | ENSG00000100985 | MMP9; matrix metalloproteinase-9 (gelatinase B) [EC:3.4.24.35] |
| K04467 | ENSG00000213341 | IKBKA, IKKA, CHUK; inhibitor of nuclear factor kappa-B kinase subunit alpha [EC:2.7.11.10] |
| K04519 | ENSG00000125538 | IL1B; interleukin 1 beta |
| K11987 | ENSG00000073756 | PTGS2, COX2; prostaglandin-endoperoxide synthase 2 [EC:1.14.99.1] |

| 20 | VEGF signaling pathway | ko04370 || K02677 | ENSG00000154229 | PRKCA; classical protein kinase C alpha type [EC:2.7.11.13] |
| K11987 | ENSG00000073756 | PTGS2, COX2; prostaglandin-endoperoxide synthase 2 [EC:1.14.99.1] |
| K19662 | ENSG00000166501 | PRKCB; classical protein kinase C beta type [EC:2.7.11.13] |

| 21 | Dopaminergic synapse | ko04728 || K02677 | ENSG00000154229 | PRKCA; classical protein kinase C alpha type [EC:2.7.11.13] |
| K04145 | ENSG00000149295 | DRD2; dopamine receptor D2 |
| K04147 | ENSG00000069696 | DRD4; dopamine receptor D4 |
| K19662 | ENSG00000166501 | PRKCB; classical protein kinase C beta type [EC:2.7.11.13] |

| 22 | Fluid shear stress and atherosclerosis | ko05418 || K01403 | ENSG00000100985 | MMP9; matrix metalloproteinase-9 (gelatinase B) [EC:3.4.24.35] |
| K04467 | ENSG00000213341 | IKBKA, IKKA, CHUK; inhibitor of nuclear factor kappa-B kinase subunit alpha [EC:2.7.11.10] |
| K04519 | ENSG00000125538 | IL1B; interleukin 1 beta |
| K04687 | ENSG00000111537 | IFNG; interferon gamma |

| 23 | Arachidonic acid metabolism | ko00590 || K00461 | ENSG00000012779 | ALOX5; arachidonate 5-lipoxygenase [EC:1.13.11.34] |
| K00509 | ENSG00000095303 | PTGS1, COX1; prostaglandin-endoperoxide synthase 1 [EC:1.14.99.1] |
| K11987 | ENSG00000073756 | PTGS2, COX2; prostaglandin-endoperoxide synthase 2 [EC:1.14.99.1] |

| 24 | Leishmaniasis | ko05140 || K04519 | ENSG00000125538 | IL1B; interleukin 1 beta |
| K04687 | ENSG00000111537 | IFNG; interferon gamma |
| K11987 | ENSG00000073756 | PTGS2, COX2; prostaglandin-endoperoxide synthase 2 [EC:1.14.99.1] |
| K19662 | ENSG00000166501 | PRKCB; classical protein kinase C beta type [EC:2.7.11.13] |

| 25 | Non-small cell lung cancer | ko05223 || K02677 | ENSG00000154229 | PRKCA; classical protein kinase C alpha type [EC:2.7.11.13] |
| K08524 | ENSG00000186350 | RXRA, NR2B1; retinoid X receptor alpha |
| K19662 | ENSG00000166501 | PRKCB; classical protein kinase C beta type [EC:2.7.11.13] |

| 26 | Hepatitis B | ko05161 || K01403 | ENSG00000100985 | MMP9; matrix metalloproteinase-9 (gelatinase B) [EC:3.4.24.35] |
| K02677 | ENSG00000154229 | PRKCA; classical protein kinase C alpha type [EC:2.7.11.13] |
| K04467 | ENSG00000213341 | IKBKA, IKKA, CHUK; inhibitor of nuclear factor kappa-B kinase subunit alpha [EC:2.7.11.10] |
| K19662 | ENSG00000166501 | PRKCB; classical protein kinase C beta type [EC:2.7.11.13] |

| 27 | Gastric acid secretion | ko04971 || K02677 | ENSG00000154229 | PRKCA; classical protein kinase C alpha type [EC:2.7.11.13] |
| K04131 | ENSG00000133019 | CHRM3; muscarinic acetylcholine receptor M3 |
| K19662 | ENSG00000166501 | PRKCB; classical protein kinase C beta type [EC:2.7.11.13] |

| 28 | MicroRNAs in cancer | ko05206 || K01403 | ENSG00000100985 | MMP9; matrix metalloproteinase-9 (gelatinase B) [EC:3.4.24.35] |
| K02677 | ENSG00000154229 | PRKCA; classical protein kinase C alpha type [EC:2.7.11.13] |
| K11987 | ENSG00000073756 | PTGS2, COX2; prostaglandin-endoperoxide synthase 2 [EC:1.14.99.1] |
| K19662 | ENSG00000166501 | PRKCB; classical protein kinase C beta type [EC:2.7.11.13] |

| 29 | Insulin secretion | ko04911 || K02677 | ENSG00000154229 | PRKCA; classical protein kinase C alpha type [EC:2.7.11.13] |
| K04131 | ENSG00000133019 | CHRM3; muscarinic acetylcholine receptor M3 |
| K19662 | ENSG00000166501 | PRKCB; classical protein kinase C beta type [EC:2.7.11.13] |

| 30 | Gap junction | ko04540 || K02677 | ENSG00000154229 | PRKCA; classical protein kinase C alpha type [EC:2.7.11.13] |
| K04145 | ENSG00000149295 | DRD2; dopamine receptor D2 |
| K19662 | ENSG00000166501 | PRKCB; classical protein kinase C beta type [EC:2.7.11.13] |

| 31 | Salivary secretion | ko04970 || K02677 | ENSG00000154229 | PRKCA; classical protein kinase C alpha type [EC:2.7.11.13] |
| K04131 | ENSG00000133019 | CHRM3; muscarinic acetylcholine receptor M3 |
| K19662 | ENSG00000166501 | PRKCB; classical protein kinase C beta type [EC:2.7.11.13] |

| 32 | Small cell lung cancer | ko05222 || K04467 | ENSG00000213341 | IKBKA, IKKA, CHUK; inhibitor of nuclear factor kappa-B kinase subunit alpha [EC:2.7.11.10] |
| K08524 | ENSG00000186350 | RXRA, NR2B1; retinoid X receptor alpha |
| K11987 | ENSG00000073756 | PTGS2, COX2; prostaglandin-endoperoxide synthase 2 [EC:1.14.99.1] |

| 33 | Inflammatory mediator regulation of TRP channels | ko04750 || K02677 | ENSG00000154229 | PRKCA; classical protein kinase C alpha type [EC:2.7.11.13] |
| K04519 | ENSG00000125538 | IL1B; interleukin 1 beta |
| K19662 | ENSG00000166501 | PRKCB; classical protein kinase C beta type [EC:2.7.11.13] |

| 34 | Morphine addiction | ko05032 || K02677 | ENSG00000154229 | PRKCA; classical protein kinase C alpha type [EC:2.7.11.13] |
| K04215 | ENSG00000112038 | OPRM1; mu-type opioid receptor |
| K19662 | ENSG00000166501 | PRKCB; classical protein kinase C beta type [EC:2.7.11.13] |

| 35 | Prostate cancer | ko05215 || K01403 | ENSG00000100985 | MMP9; matrix metalloproteinase-9 (gelatinase B) [EC:3.4.24.35] |
| K04467 | ENSG00000213341 | IKBKA, IKKA, CHUK; inhibitor of nuclear factor kappa-B kinase subunit alpha [EC:2.7.11.10] |
| K08557 | ENSG00000169083 | AR, NR3C4; androgen receptor |

| 36 | Pancreatic secretion | ko04972 || K02677 | ENSG00000154229 | PRKCA; classical protein kinase C alpha type [EC:2.7.11.13] |
| K04131 | ENSG00000133019 | CHRM3; muscarinic acetylcholine receptor M3 |
| K19662 | ENSG00000166501 | PRKCB; classical protein kinase C beta type [EC:2.7.11.13] |

| 37 | Choline metabolism in cancer | ko05231 || K02677 | ENSG00000154229 | PRKCA; classical protein kinase C alpha type [EC:2.7.11.13] |
| K08268 | ENSG00000100644 | HIF1A; hypoxia-inducible factor 1 alpha |
| K19662 | ENSG00000166501 | PRKCB; classical protein kinase C beta type [EC:2.7.11.13] |

| 38 | Aldosterone-regulated sodium reabsorption | ko04960 || K02677 | ENSG00000154229 | PRKCA; classical protein kinase C alpha type [EC:2.7.11.13] |
| K19662 | ENSG00000166501 | PRKCB; classical protein kinase C beta type [EC:2.7.11.13] |

| 39 | C-type lectin receptor signaling pathway | ko04625 || K04467 | ENSG00000213341 | IKBKA, IKKA, CHUK; inhibitor of nuclear factor kappa-B kinase subunit alpha [EC:2.7.11.10] |
| K04519 | ENSG00000125538 | IL1B; interleukin 1 beta |
| K11987 | ENSG00000073756 | PTGS2, COX2; prostaglandin-endoperoxide synthase 2 [EC:1.14.99.1] |

| 40 | Leukocyte transendothelial migration | ko04670 || K01403 | ENSG00000100985 | MMP9; matrix metalloproteinase-9 (gelatinase B) [EC:3.4.24.35] |
| K02677 | ENSG00000154229 | PRKCA; classical protein kinase C alpha type [EC:2.7.11.13] |
| K19662 | ENSG00000166501 | PRKCB; classical protein kinase C beta type [EC:2.7.11.13] |

| 41 | Parathyroid hormone synthesis, secretion and action | ko04928 || K02677 | ENSG00000154229 | PRKCA; classical protein kinase C alpha type [EC:2.7.11.13] |
| K08524 | ENSG00000186350 | RXRA, NR2B1; retinoid X receptor alpha |
| K19662 | ENSG00000166501 | PRKCB; classical protein kinase C beta type [EC:2.7.11.13] |

| 42 | Antifolate resistance | ko01523 || K04467 | ENSG00000213341 | IKBKA, IKKA, CHUK; inhibitor of nuclear factor kappa-B kinase subunit alpha [EC:2.7.11.10] |
| K04519 | ENSG00000125538 | IL1B; interleukin 1 beta |

| 43 | PI3K-Akt signaling pathway | ko04151 || K02677 | ENSG00000154229 | PRKCA; classical protein kinase C alpha type [EC:2.7.11.13] |
| K04130 | ENSG00000181072 | CHRM2; muscarinic acetylcholine receptor M2 |
| K04467 | ENSG00000213341 | IKBKA, IKKA, CHUK; inhibitor of nuclear factor kappa-B kinase subunit alpha [EC:2.7.11.10] |
| K06236 | ENSG00000108821 | COL1A; collagen, type I, alpha |
| K08524 | ENSG00000186350 | RXRA, NR2B1; retinoid X receptor alpha |

| 44 | Relaxin signaling pathway | ko04926 || K01403 | ENSG00000100985 | MMP9; matrix metalloproteinase-9 (gelatinase B) [EC:3.4.24.35] |
| K02677 | ENSG00000154229 | PRKCA; classical protein kinase C alpha type [EC:2.7.11.13] |
| K06236 | ENSG00000108821 | COL1A; collagen, type I, alpha |

| 45 | Ovarian Steroidogenesis | ko04913 || K00461 | ENSG00000012779 | ALOX5; arachidonate 5-lipoxygenase [EC:1.13.11.34] |
| K11987 | ENSG00000073756 | PTGS2, COX2; prostaglandin-endoperoxide synthase 2 [EC:1.14.99.1] |

| 46 | Malaria | ko05144 || K04519 | ENSG00000125538 | IL1B; interleukin 1 beta |
| K04687 | ENSG00000111537 | IFNG; interferon gamma |

| 47 | Influenza A | ko05164 || K02677 | ENSG00000154229 | PRKCA; classical protein kinase C alpha type [EC:2.7.11.13] |
| K04519 | ENSG00000125538 | IL1B; interleukin 1 beta |
| K04687 | ENSG00000111537 | IFNG; interferon gamma |
| K19662 | ENSG00000166501 | PRKCB; classical protein kinase C beta type [EC:2.7.11.13] |

| 48 | Measles | ko05162 || K04467 | ENSG00000213341 | IKBKA, IKKA, CHUK; inhibitor of nuclear factor kappa-B kinase subunit alpha [EC:2.7.11.10] |
| K04519 | ENSG00000125538 | IL1B; interleukin 1 beta |
| K04687 | ENSG00000111537 | IFNG; interferon gamma |

| 49 | Oxytocin signaling pathway | ko04921 || K02677 | ENSG00000154229 | PRKCA; classical protein kinase C alpha type [EC:2.7.11.13] |
| K11987 | ENSG00000073756 | PTGS2, COX2; prostaglandin-endoperoxide synthase 2 [EC:1.14.99.1] |
| K19662 | ENSG00000166501 | PRKCB; classical protein kinase C beta type [EC:2.7.11.13] |

| 50 | Long-term depression | ko04730 || K02677 | ENSG00000154229 | PRKCA; classical protein kinase C alpha type [EC:2.7.11.13] |
| K19662 | ENSG00000166501 | PRKCB; classical protein kinase C beta type [EC:2.7.11.13] |

| 51 | Retrograde endocannabinoid signaling | ko04723 || K02677 | ENSG00000154229 | PRKCA; classical protein kinase C alpha type [EC:2.7.11.13] |
| K11987 | ENSG00000073756 | PTGS2, COX2; prostaglandin-endoperoxide synthase 2 [EC:1.14.99.1] |
| K19662 | ENSG00000166501 | PRKCB; classical protein kinase C beta type [EC:2.7.11.13] |

| 52 | Estrogen signaling pathway | ko04915 || K01403 | ENSG00000100985 | MMP9; matrix metalloproteinase-9 (gelatinase B) [EC:3.4.24.35] |
| K04215 | ENSG00000112038 | OPRM1; mu-type opioid receptor |
| K08550 | ENSG00000091831 | ESR1, NR3A1; estrogen receptor alpha |

| 53 | cGMP - PKG signaling pathway | ko04022 || K04138 | ENSG00000150594 | ADRA2A; adrenergic receptor alpha-2A |
| K04139 | ENSG00000274286 | ADRA2B; adrenergic receptor alpha-2B |
| K04140 | ENSG00000184160 | ADRA2C; adrenergic receptor alpha-2C |

| 54 | mTOR signaling pathway | ko04150 || K02677 | ENSG00000154229 | PRKCA; classical protein kinase C alpha type [EC:2.7.11.13] |
| K04467 | ENSG00000213341 | IKBKA, IKKA, CHUK; inhibitor of nuclear factor kappa-B kinase subunit alpha [EC:2.7.11.10] |
| K19662 | ENSG00000166501 | PRKCB; classical protein kinase C beta type [EC:2.7.11.13] |

| 55 | Acute myeloid leukemia | ko05221 || K04467 | ENSG00000213341 | IKBKA, IKKA, CHUK; inhibitor of nuclear factor kappa-B kinase subunit alpha [EC:2.7.11.10] |
| K10789 | ENSG00000005381 | MPO; myeloperoxidase [EC:1.11.2.2] |

| 56 | Long-term potentiation | ko04720 || K02677 | ENSG00000154229 | PRKCA; classical protein kinase C alpha type [EC:2.7.11.13] |
| K19662 | ENSG00000166501 | PRKCB; classical protein kinase C beta type [EC:2.7.11.13] |

| 57 | Cytosolic DNA-sensing pathway | ko04623 || K04467 | ENSG00000213341 | IKBKA, IKKA, CHUK; inhibitor of nuclear factor kappa-B kinase subunit alpha [EC:2.7.11.10] |
| K04519 | ENSG00000125538 | IL1B; interleukin 1 beta |

| 58 | Amphetamine addiction | ko05031 || K02677 | ENSG00000154229 | PRKCA; classical protein kinase C alpha type [EC:2.7.11.13] |
| K19662 | ENSG00000166501 | PRKCB; classical protein kinase C beta type [EC:2.7.11.13] |

| 59 | B cell receptor signaling pathway | ko04662 || K04467 | ENSG00000213341 | IKBKA, IKKA, CHUK; inhibitor of nuclear factor kappa-B kinase subunit alpha [EC:2.7.11.10] |
| K19662 | ENSG00000166501 | PRKCB; classical protein kinase C beta type [EC:2.7.11.13] |

| 60 | Osteoclast differentiation | ko04380 || K04467 | ENSG00000213341 | IKBKA, IKKA, CHUK; inhibitor of nuclear factor kappa-B kinase subunit alpha [EC:2.7.11.10] |
| K04519 | ENSG00000125538 | IL1B; interleukin 1 beta |
| K04687 | ENSG00000111537 | IFNG; interferon gamma |

| 61 | Bile secretion | ko04976 || K08524 | ENSG00000186350 | RXRA, NR2B1; retinoid X receptor alpha |
| K17689 | ENSG00000160868 | CYP3A4; cytochrome P450 family 3 subfamily A polypeptide 4 [EC:1.14.13.32 1.14.14.55 1.14.14.56 1.14.14.57 1.14.14.73 1.14.13.-] |

| 62 | Glioma | ko05214 || K02677 | ENSG00000154229 | PRKCA; classical protein kinase C alpha type [EC:2.7.11.13] |
| K19662 | ENSG00000166501 | PRKCB; classical protein kinase C beta type [EC:2.7.11.13] |

| 63 | MAPK signaling pathway | ko04010 || K02677 | ENSG00000154229 | PRKCA; classical protein kinase C alpha type [EC:2.7.11.13] |
| K04467 | ENSG00000213341 | IKBKA, IKKA, CHUK; inhibitor of nuclear factor kappa-B kinase subunit alpha [EC:2.7.11.10] |
| K04519 | ENSG00000125538 | IL1B; interleukin 1 beta |
| K19662 | ENSG00000166501 | PRKCB; classical protein kinase C beta type [EC:2.7.11.13] |

| 64 | Transcriptional misregulation in cancers | ko05202 || K01403 | ENSG00000100985 | MMP9; matrix metalloproteinase-9 (gelatinase B) [EC:3.4.24.35] |
| K08524 | ENSG00000186350 | RXRA, NR2B1; retinoid X receptor alpha |
| K10789 | ENSG00000005381 | MPO; myeloperoxidase [EC:1.11.2.2] |

| 65 | PPAR signaling pathway | ko03320 || K01059 | ENSG00000175445 | LPL; lipoprotein lipase [EC:3.1.1.34] |
| K08524 | ENSG00000186350 | RXRA, NR2B1; retinoid X receptor alpha |

| 66 | Fc epsilon RI signaling pathway | ko04664 || K00461 | ENSG00000012779 | ALOX5; arachidonate 5-lipoxygenase [EC:1.13.11.34] |
| K02677 | ENSG00000154229 | PRKCA; classical protein kinase C alpha type [EC:2.7.11.13] |

| 67 | EGFR tyrosine kinase inhibitor resistance | ko01521 || K02677 | ENSG00000154229 | PRKCA; classical protein kinase C alpha type [EC:2.7.11.13] |
| K19662 | ENSG00000166501 | PRKCB; classical protein kinase C beta type [EC:2.7.11.13] |

| 68 | Thyroid hormone synthesis | ko04918 || K02677 | ENSG00000154229 | PRKCA; classical protein kinase C alpha type [EC:2.7.11.13] |
| K19662 | ENSG00000166501 | PRKCB; classical protein kinase C beta type [EC:2.7.11.13] |

| 69 | Drug metabolism - other enzymes | ko00983 || K10789 | ENSG00000005381 | MPO; myeloperoxidase [EC:1.11.2.2] |
| K17689 | ENSG00000160868 | CYP3A4; cytochrome P450 family 3 subfamily A polypeptide 4 [EC:1.14.13.32 1.14.14.55 1.14.14.56 1.14.14.57 1.14.14.73 1.14.13.-] |

| 70 | Toxoplasmosis | ko05145 || K00461 | ENSG00000012779 | ALOX5; arachidonate 5-lipoxygenase [EC:1.13.11.34] |
| K04467 | ENSG00000213341 | IKBKA, IKKA, CHUK; inhibitor of nuclear factor kappa-B kinase subunit alpha [EC:2.7.11.10] |
| K04687 | ENSG00000111537 | IFNG; interferon gamma |

| 71 | cAMP signaling pathway | ko04024 || K04130 | ENSG00000181072 | CHRM2; muscarinic acetylcholine receptor M2 |
| K04145 | ENSG00000149295 | DRD2; dopamine receptor D2 |
| K04260 | ENSG00000050628 | PTGER3; prostaglandin E receptor 3 |

| 72 | ErbB signaling pathway | ko04012 || K02677 | ENSG00000154229 | PRKCA; classical protein kinase C alpha type [EC:2.7.11.13] |
| K19662 | ENSG00000166501 | PRKCB; classical protein kinase C beta type [EC:2.7.11.13] |

| 73 | Adipocytokine signaling pathway | ko04920 || K04467 | ENSG00000213341 | IKBKA, IKKA, CHUK; inhibitor of nuclear factor kappa-B kinase subunit alpha [EC:2.7.11.10] |
| K08524 | ENSG00000186350 | RXRA, NR2B1; retinoid X receptor alpha |

| 74 | Rap1 signaling pathway | ko04015 || K02677 | ENSG00000154229 | PRKCA; classical protein kinase C alpha type [EC:2.7.11.13] |
| K04145 | ENSG00000149295 | DRD2; dopamine receptor D2 |
| K19662 | ENSG00000166501 | PRKCB; classical protein kinase C beta type [EC:2.7.11.13] |

| 75 | Focal adhesion | ko04510 || K02677 | ENSG00000154229 | PRKCA; classical protein kinase C alpha type [EC:2.7.11.13] |
| K06236 | ENSG00000108821 | COL1A; collagen, type I, alpha |
| K19662 | ENSG00000166501 | PRKCB; classical protein kinase C beta type [EC:2.7.11.13] |

| 76 | Chemical carcinogenesis | ko05204 || K11987 | ENSG00000073756 | PTGS2, COX2; prostaglandin-endoperoxide synthase 2 [EC:1.14.99.1] |
| K17689 | ENSG00000160868 | CYP3A4; cytochrome P450 family 3 subfamily A polypeptide 4 [EC:1.14.13.32 1.14.14.55 1.14.14.56 1.14.14.57 1.14.14.73 1.14.13.-] |

| 77 | Fc gamma R-mediated phagocytosis | ko04666 || K02677 | ENSG00000154229 | PRKCA; classical protein kinase C alpha type [EC:2.7.11.13] |
| K19662 | ENSG00000166501 | PRKCB; classical protein kinase C beta type [EC:2.7.11.13] |

| 78 | GnRH signaling pathway | ko04912 || K02677 | ENSG00000154229 | PRKCA; classical protein kinase C alpha type [EC:2.7.11.13] |
| K19662 | ENSG00000166501 | PRKCB; classical protein kinase C beta type [EC:2.7.11.13] |

| 79 | Salmonella infection | ko05132 || K04519 | ENSG00000125538 | IL1B; interleukin 1 beta |
| K04687 | ENSG00000111537 | IFNG; interferon gamma |

| 80 | Circadian entrainment | ko04713 || K02677 | ENSG00000154229 | PRKCA; classical protein kinase C alpha type [EC:2.7.11.13] |
| K19662 | ENSG00000166501 | PRKCB; classical protein kinase C beta type [EC:2.7.11.13] |

| 81 | GABAergic synapse | ko04727 || K02677 | ENSG00000154229 | PRKCA; classical protein kinase C alpha type [EC:2.7.11.13] |
| K19662 | ENSG00000166501 | PRKCB; classical protein kinase C beta type [EC:2.7.11.13] |

| 82 | Phosphatidylinositol signaling system | ko04070 || K02677 | ENSG00000154229 | PRKCA; classical protein kinase C alpha type [EC:2.7.11.13] |
| K19662 | ENSG00000166501 | PRKCB; classical protein kinase C beta type [EC:2.7.11.13] |

| 83 | Melanogenesis | ko04916 || K02677 | ENSG00000154229 | PRKCA; classical protein kinase C alpha type [EC:2.7.11.13] |
| K19662 | ENSG00000166501 | PRKCB; classical protein kinase C beta type [EC:2.7.11.13] |

| 84 | Kaposi sarcoma-associated herpesvirus infection | ko05167 || K04467 | ENSG00000213341 | IKBKA, IKKA, CHUK; inhibitor of nuclear factor kappa-B kinase subunit alpha [EC:2.7.11.10] |
| K08268 | ENSG00000100644 | HIF1A; hypoxia-inducible factor 1 alpha |
| K11987 | ENSG00000073756 | PTGS2, COX2; prostaglandin-endoperoxide synthase 2 [EC:1.14.99.1] |

| 85 | Ras signaling pathway | ko04014 || K02677 | ENSG00000154229 | PRKCA; classical protein kinase C alpha type [EC:2.7.11.13] |
| K04467 | ENSG00000213341 | IKBKA, IKKA, CHUK; inhibitor of nuclear factor kappa-B kinase subunit alpha [EC:2.7.11.10] |
| K19662 | ENSG00000166501 | PRKCB; classical protein kinase C beta type [EC:2.7.11.13] |

| 86 | Aldosterone synthesis and secretion | ko04925 || K02677 | ENSG00000154229 | PRKCA; classical protein kinase C alpha type [EC:2.7.11.13] |
| K19662 | ENSG00000166501 | PRKCB; classical protein kinase C beta type [EC:2.7.11.13] |

| 87 | T cell receptor signaling pathway | ko04660 || K04467 | ENSG00000213341 | IKBKA, IKKA, CHUK; inhibitor of nuclear factor kappa-B kinase subunit alpha [EC:2.7.11.10] |
| K04687 | ENSG00000111537 | IFNG; interferon gamma |

| 88 | Endocrine resistance | ko01522 || K01403 | ENSG00000100985 | MMP9; matrix metalloproteinase-9 (gelatinase B) [EC:3.4.24.35] |
| K08550 | ENSG00000091831 | ESR1, NR3A1; estrogen receptor alpha |

| 89 | Taste transduction | ko04742 || K04131 | ENSG00000133019 | CHRM3; muscarinic acetylcholine receptor M3 |
| K04819 | ENSG00000166736 | HTR3; 5-hydroxytryptamine receptor 3 |

| 90 | Glutamatergic synapse | ko04724 || K02677 | ENSG00000154229 | PRKCA; classical protein kinase C alpha type [EC:2.7.11.13] |
| K19662 | ENSG00000166501 | PRKCB; classical protein kinase C beta type [EC:2.7.11.13] |

| 91 | Toll-like receptor signaling pathway | ko04620 || K04467 | ENSG00000213341 | IKBKA, IKKA, CHUK; inhibitor of nuclear factor kappa-B kinase subunit alpha [EC:2.7.11.10] |
| K04519 | ENSG00000125538 | IL1B; interleukin 1 beta |

| 92 | Vascular smooth muscle contraction | ko04270 || K02677 | ENSG00000154229 | PRKCA; classical protein kinase C alpha type [EC:2.7.11.13] |
| K19662 | ENSG00000166501 | PRKCB; classical protein kinase C beta type [EC:2.7.11.13] |

| 93 | Sphingolipid signaling pathway | ko04071 || K02677 | ENSG00000154229 | PRKCA; classical protein kinase C alpha type [EC:2.7.11.13] |
| K19662 | ENSG00000166501 | PRKCB; classical protein kinase C beta type [EC:2.7.11.13] |

| 94 | Platelet activation | ko04611 || K00509 | ENSG00000095303 | PTGS1, COX1; prostaglandin-endoperoxide synthase 1 [EC:1.14.99.1] |
| K06236 | ENSG00000108821 | COL1A; collagen, type I, alpha |

| 95 | Hepatitis C | ko05160 || K04467 | ENSG00000213341 | IKBKA, IKKA, CHUK; inhibitor of nuclear factor kappa-B kinase subunit alpha [EC:2.7.11.10] |
| K08524 | ENSG00000186350 | RXRA, NR2B1; retinoid X receptor alpha |

| 96 | Inflammatiory bowel disease (IBD) | ko05321 || K04519 | ENSG00000125538 | IL1B; interleukin 1 beta |
| K04687 | ENSG00000111537 | IFNG; interferon gamma |

| 97 | Linoleic acid metabolism | ko00591 | K17689 | ENSG00000160868 | CYP3A4; cytochrome P450 family 3 subfamily A polypeptide 4 [EC:1.14.13.32 1.14.14.55 1.14.14.56 1.14.14.57 1.14.14.73 1.14.13.-] |
| 98 | Wnt signaling pathway | ko04310 || K02677 | ENSG00000154229 | PRKCA; classical protein kinase C alpha type [EC:2.7.11.13] |
| K19662 | ENSG00000166501 | PRKCB; classical protein kinase C beta type [EC:2.7.11.13] |

| 99 | Type I diabetes mellitus | ko04940 || K04519 | ENSG00000125538 | IL1B; interleukin 1 beta |
| K04687 | ENSG00000111537 | IFNG; interferon gamma |

| 100 | Th1 and Th2 cell differentiation | ko04658 || K04467 | ENSG00000213341 | IKBKA, IKKA, CHUK; inhibitor of nuclear factor kappa-B kinase subunit alpha [EC:2.7.11.10] |
| K04687 | ENSG00000111537 | IFNG; interferon gamma |

| 101 | Herpes simplex infection | ko05168 || K04467 | ENSG00000213341 | IKBKA, IKKA, CHUK; inhibitor of nuclear factor kappa-B kinase subunit alpha [EC:2.7.11.10] |
| K04519 | ENSG00000125538 | IL1B; interleukin 1 beta |
| K04687 | ENSG00000111537 | IFNG; interferon gamma |

| 102 | Necroptosis | ko04217 || K04519 | ENSG00000125538 | IL1B; interleukin 1 beta |
| K04687 | ENSG00000111537 | IFNG; interferon gamma |

| 103 | Prion diseases | ko05020 | K04519 | ENSG00000125538 | IL1B; interleukin 1 beta |
| 104 | Non-alcoholic fatty liver disease (NAFLD) | ko04932 || K04519 | ENSG00000125538 | IL1B; interleukin 1 beta |
| K08524 | ENSG00000186350 | RXRA, NR2B1; retinoid X receptor alpha |

| 105 | Hepatocellular carcinoma | ko05225 || K02677 | ENSG00000154229 | PRKCA; classical protein kinase C alpha type [EC:2.7.11.13] |
| K19662 | ENSG00000166501 | PRKCB; classical protein kinase C beta type [EC:2.7.11.13] |

| 106 | Thyroid cancer | ko05216 | K08524 | ENSG00000186350 | RXRA, NR2B1; retinoid X receptor alpha |
| 107 | Bladder cancer | ko05219 | K01403 | ENSG00000100985 | MMP9; matrix metalloproteinase-9 (gelatinase B) [EC:3.4.24.35] |
| 108 | Rheumatoid arthritis | ko05323 || K04519 | ENSG00000125538 | IL1B; interleukin 1 beta |
| K04687 | ENSG00000111537 | IFNG; interferon gamma |

| 109 | NOD-like receptor signaling pathway | ko04621 || K04467 | ENSG00000213341 | IKBKA, IKKA, CHUK; inhibitor of nuclear factor kappa-B kinase subunit alpha [EC:2.7.11.10] |
| K04519 | ENSG00000125538 | IL1B; interleukin 1 beta |

| 110 | Alzheimer disease | ko05010 || K01059 | ENSG00000175445 | LPL; lipoprotein lipase [EC:3.1.1.34] |
| K04519 | ENSG00000125538 | IL1B; interleukin 1 beta |

| 111 | Carbohydrate digestion and absorption | ko04973 | K19662 | ENSG00000166501 | PRKCB; classical protein kinase C beta type [EC:2.7.11.13] |
| 112 | Cholesterol metabolism | ko04979 | K01059 | ENSG00000175445 | LPL; lipoprotein lipase [EC:3.1.1.34] |
| 113 | Chemokine signaling pathway | ko04062 || K04467 | ENSG00000213341 | IKBKA, IKKA, CHUK; inhibitor of nuclear factor kappa-B kinase subunit alpha [EC:2.7.11.10] |
| K19662 | ENSG00000166501 | PRKCB; classical protein kinase C beta type [EC:2.7.11.13] |

| 114 | Cocaine addiction | ko05030 | K04145 | ENSG00000149295 | DRD2; dopamine receptor D2 |
| 115 | Regulation of actin cytoskeleton | ko04810 || K04130 | ENSG00000181072 | CHRM2; muscarinic acetylcholine receptor M2 |
| K04131 | ENSG00000133019 | CHRM3; muscarinic acetylcholine receptor M3 |

| 116 | Human papillomavirus infection | ko05165 || K04467 | ENSG00000213341 | IKBKA, IKKA, CHUK; inhibitor of nuclear factor kappa-B kinase subunit alpha [EC:2.7.11.10] |
| K06236 | ENSG00000108821 | COL1A; collagen, type I, alpha |
| K11987 | ENSG00000073756 | PTGS2, COX2; prostaglandin-endoperoxide synthase 2 [EC:1.14.99.1] |

| 117 | Vibrio cholerae infection | ko05110 | K02677 | ENSG00000154229 | PRKCA; classical protein kinase C alpha type [EC:2.7.11.13] |
| 118 | Pathogenic Escherichia coli infection | ko05130 | K02677 | ENSG00000154229 | PRKCA; classical protein kinase C alpha type [EC:2.7.11.13] |
| 119 | Proteasome | ko03050 | K04687 | ENSG00000111537 | IFNG; interferon gamma |
| 120 | Shigellosis | ko05131 | K04467 | ENSG00000213341 | IKBKA, IKKA, CHUK; inhibitor of nuclear factor kappa-B kinase subunit alpha [EC:2.7.11.10] |
| 121 | Central carbon metabolism in cancer | ko05230 | K08268 | ENSG00000100644 | HIF1A; hypoxia-inducible factor 1 alpha |
| 122 | Glycerolipid metabolism | ko00561 | K01059 | ENSG00000175445 | LPL; lipoprotein lipase [EC:3.1.1.34] |
| 123 | Renal cell carcinoma | ko05211 | K08268 | ENSG00000100644 | HIF1A; hypoxia-inducible factor 1 alpha |
| 124 | Steroid hormone biosynthesis | ko00140 | K17689 | ENSG00000160868 | CYP3A4; cytochrome P450 family 3 subfamily A polypeptide 4 [EC:1.14.13.32 1.14.14.55 1.14.14.56 1.14.14.57 1.14.14.73 1.14.13.-] |
| 125 | p53 signaling pathway | ko04115 | K03982 | ENSG00000106366 | SERPINE1, PAI1; plasminogen activator inhibitor 1 |
| 126 | Mitophagy - animal | ko04137 | K08268 | ENSG00000100644 | HIF1A; hypoxia-inducible factor 1 alpha |
| 127 | Prolactin signaling pathway | ko04917 | K08550 | ENSG00000091831 | ESR1, NR3A1; estrogen receptor alpha |
| 128 | Retinol metabolism | ko00830 | K17689 | ENSG00000160868 | CYP3A4; cytochrome P450 family 3 subfamily A polypeptide 4 [EC:1.14.13.32 1.14.14.55 1.14.14.56 1.14.14.57 1.14.14.73 1.14.13.-] |
| 129 | Tuberculosis | ko05152 || K04519 | ENSG00000125538 | IL1B; interleukin 1 beta |
| K04687 | ENSG00000111537 | IFNG; interferon gamma |

| 130 | Legionellosis | ko05134 | K04519 | ENSG00000125538 | IL1B; interleukin 1 beta |
| 131 | Pancreatic cancer | ko05212 | K04467 | ENSG00000213341 | IKBKA, IKKA, CHUK; inhibitor of nuclear factor kappa-B kinase subunit alpha [EC:2.7.11.10] |
| 132 | Epithelial cell signaling in Helicobacter pylori infection | ko05120 | K04467 | ENSG00000213341 | IKBKA, IKKA, CHUK; inhibitor of nuclear factor kappa-B kinase subunit alpha [EC:2.7.11.10] |
| 133 | RIG-I-like receptor signaling pathway | ko04622 | K04467 | ENSG00000213341 | IKBKA, IKKA, CHUK; inhibitor of nuclear factor kappa-B kinase subunit alpha [EC:2.7.11.10] |
| 134 | Chronic myeloid leukemia | ko05220 | K04467 | ENSG00000213341 | IKBKA, IKKA, CHUK; inhibitor of nuclear factor kappa-B kinase subunit alpha [EC:2.7.11.10] |
| 135 | Drug metabolism - cytochrome P450 | ko00982 | K17689 | ENSG00000160868 | CYP3A4; cytochrome P450 family 3 subfamily A polypeptide 4 [EC:1.14.13.32 1.14.14.55 1.14.14.56 1.14.14.57 1.14.14.73 1.14.13.-] |
| 136 | Metabolism of xenobiotics by cytochrome P450 | ko00980 | K17689 | ENSG00000160868 | CYP3A4; cytochrome P450 family 3 subfamily A polypeptide 4 [EC:1.14.13.32 1.14.14.55 1.14.14.56 1.14.14.57 1.14.14.73 1.14.13.-] |
| 137 | TGF-beta signaling pathway | ko04350 | K04687 | ENSG00000111537 | IFNG; interferon gamma |
| 138 | Pertussis | ko05133 | K04519 | ENSG00000125538 | IL1B; interleukin 1 beta |
| 139 | Natural killer cell mediated cytotoxicity | ko04650 || K02677 | ENSG00000154229 | PRKCA; classical protein kinase C alpha type [EC:2.7.11.13] |
| K04687 | ENSG00000111537 | IFNG; interferon gamma |
| K19662 | ENSG00000166501 | PRKCB; classical protein kinase C beta type [EC:2.7.11.13] |

| 140 | Complement and coagulation cascades | ko04610 | K03982 | ENSG00000106366 | SERPINE1, PAI1; plasminogen activator inhibitor 1 |
| 141 | ECM-receptor interaction | ko04512 | K06236 | ENSG00000108821 | COL1A; collagen, type I, alpha |
| 142 | Protein digestion and absorption | ko04974 | K06236 | ENSG00000108821 | COL1A; collagen, type I, alpha |
| 143 | Epstein-Barr virus infection | ko05169 || K04467 | ENSG00000213341 | IKBKA, IKKA, CHUK; inhibitor of nuclear factor kappa-B kinase subunit alpha [EC:2.7.11.10] |
| K04687 | ENSG00000111537 | IFNG; interferon gamma |

| 144 | Graft-versus-host disease | ko05332 || K04519 | ENSG00000125538 | IL1B; interleukin 1 beta |
| K04687 | ENSG00000111537 | IFNG; interferon gamma |

| 145 | Cytokine-cytokine receptor interaction | ko04060 || K04519 | ENSG00000125538 | IL1B; interleukin 1 beta |
| K04687 | ENSG00000111537 | IFNG; interferon gamma |

| 146 | Glycerophospholipid metabolism | ko00564 | K01049 | ENSG00000087085 | ACHE; acetylcholinesterase [EC:3.1.1.7] |
| 147 | Insulin resistance | ko04931 | K19662 | ENSG00000166501 | PRKCB; classical protein kinase C beta type [EC:2.7.11.13] |
| 148 | Oocyte meiosis | ko04114 | K08557 | ENSG00000169083 | AR, NR3C4; androgen receptor |
| 149 | Autophagy - animal | ko04140 | K08268 | ENSG00000100644 | HIF1A; hypoxia-inducible factor 1 alpha |
| 150 | FoxO signaling pathway | ko04068 | K04467 | ENSG00000213341 | IKBKA, IKKA, CHUK; inhibitor of nuclear factor kappa-B kinase subunit alpha [EC:2.7.11.10] |
| 151 | Apelin signaling pathway | ko04371 | K03982 | ENSG00000106366 | SERPINE1, PAI1; plasminogen activator inhibitor 1 |
| 152 | Apoptosis | ko04210 | K04467 | ENSG00000213341 | IKBKA, IKKA, CHUK; inhibitor of nuclear factor kappa-B kinase subunit alpha [EC:2.7.11.10] |
| 153 | Adrenergic signaling in cardiomyocytes | ko04261 | K02677 | ENSG00000154229 | PRKCA; classical protein kinase C alpha type [EC:2.7.11.13] |
| 154 | Allograft rejection | ko05330 | K04687 | ENSG00000111537 | IFNG; interferon gamma |
| 155 | Phospholipase D signaling pathway | ko04072 | K02677 | ENSG00000154229 | PRKCA; classical protein kinase C alpha type [EC:2.7.11.13] |
| 156 | Hippo signaling pathway | ko04390 | K03982 | ENSG00000106366 | SERPINE1, PAI1; plasminogen activator inhibitor 1 |
| 157 | Parkinson disease | ko05012 | K04145 | ENSG00000149295 | DRD2; dopamine receptor D2 |
| 158 | Gastric cancer | ko05226 | K08524 | ENSG00000186350 | RXRA, NR2B1; retinoid X receptor alpha |
| 159 | Breast cancer | ko05224 | K08550 | ENSG00000091831 | ESR1, NR3A1; estrogen receptor alpha |
| 160 | Jak-STAT signaling pathway | ko04630 | K04687 | ENSG00000111537 | IFNG; interferon gamma |
| 161 | Hematopoietic cell lineage | ko04640 | K04519 | ENSG00000125538 | IL1B; interleukin 1 beta |
| 162 | Axon guidance | ko04360 | K02677 | ENSG00000154229 | PRKCA; classical protein kinase C alpha type [EC:2.7.11.13] |
| 163 | Alcoholism | ko05034 | K04145 | ENSG00000149295 | DRD2; dopamine receptor D2 |
| 164 | Systemic lupus erythematosus | ko05322 | K04687 | ENSG00000111537 | IFNG; interferon gamma |
| 165 | Phagosome | ko04145 | K10789 | ENSG00000005381 | MPO; myeloperoxidase [EC:1.11.2.2] |
| 166 | Cellular senescence | ko04218 | K03982 | ENSG00000106366 | SERPINE1, PAI1; plasminogen activator inhibitor 1 |
| 167 | HTLV-I infection | ko05166 | K04467 | ENSG00000213341 | IKBKA, IKKA, CHUK; inhibitor of nuclear factor kappa-B kinase subunit alpha [EC:2.7.11.10] |
| 168 | Metabolic pathways | ko01100 || K00461 | ENSG00000012779 | ALOX5; arachidonate 5-lipoxygenase [EC:1.13.11.34] |
| K00509 | ENSG00000095303 | PTGS1, COX1; prostaglandin-endoperoxide synthase 1 [EC:1.14.99.1] |
| K11987 | ENSG00000073756 | PTGS2, COX2; prostaglandin-endoperoxide synthase 2 [EC:1.14.99.1] |
| K17689 | ENSG00000160868 | CYP3A4; cytochrome P450 family 3 subfamily A polypeptide 4 [EC:1.14.13.32 1.14.14.55 1.14.14.56 1.14.14.57 1.14.14.73 1.14.13.-] |

| 169 | Antigen processing and presentation | ko04612 | K04687 | ENSG00000111537 | IFNG; interferon gamma |

Back Top
