# Supplementary material for: Underlying Mechanism and Active Ingredients of Tianma Gouteng Acting on Cerebral Infarction as Determined via Network Pharmacology Analysis Combined With Experimental Validation
Source: Front Pharmacol. 2021 Nov 16;12:760503. doi: 10.3389/fphar.2021.760503 (PMC8635202; doi:10.3389/fphar.2021.760503)
Supplement: Supplementary file 2 [file DataSheet1.zip › original data/HPLC/STANDARD.pdf]

SHIMADZU LabSolutions

分析报告

<样品信息>

样品名	:		
样品ID	:		
数据文件名	: 混标0716.lcd		
方法文件名	: 2C乙腈方法68min-0714.lcm		
批处理文件名	: 0714.lcb		
样品瓶号	: 1-1	样品类型	: 未知
进样体积	: 10 uL		
分析日期	: 2021/7/16 13:56:01	分析者	: System Administrator
处理日期	: 2021/7/16 17:39:59	处理者	: System Administrator

<色谱图>

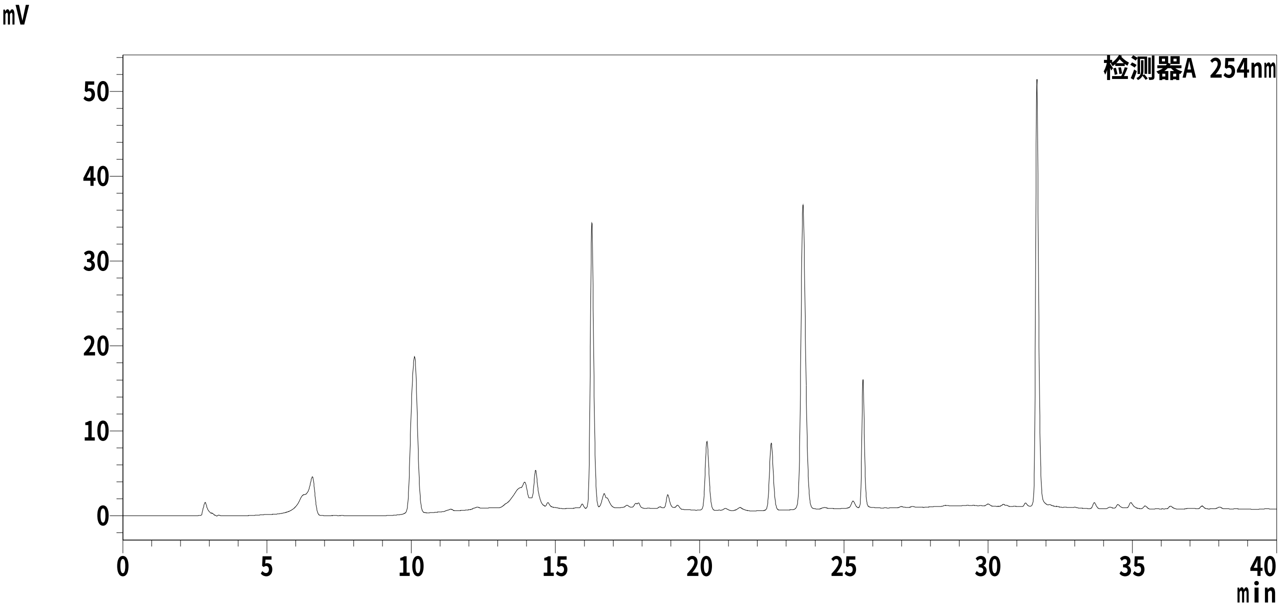

<峰表>

检测器A 254nm

峰号	保留时间	面积	高度	浓度	浓度单位	标记	化合物名
1	2.857	19569	1582	0.000			
2	5.125	3654	141	0.000			
3	6.572	134299	4563	0.000	mg/L	V	RT:6.572
4	10.115	288643	18511	0.000	mg/L		RT:10.115
5	11.375	2994	234	0.000		V	
6	12.295	2240	188	0.000			
7	13.932	92068	3063	0.000			
8	14.311	57243	4486	0.000		V	
9	14.739	7980	701	0.000		V	
10	15.773	1466	131	0.000		V	
11	15.929	4132	570	0.000		V	
12	16.261	280533	33701	0.000	mg/L	V	RT:16.261
13	16.690	30063	1822	0.000		V	
14	17.481	8908	487	0.000		V	
15	17.785	5585	720	0.000		V	
16	17.877	6864	784	0.000		V	
17	18.218	2041	192	0.000		V	
18	18.383	1221	149	0.000		V	
19	18.621	3874	344	0.000		V	
20	18.894	17709	1780	0.000		V	
21	19.235	6754	568	0.000		V	
22	20.251	76732	8101	0.000	mg/L		RT:20.251

峰号	保留时间	面积	高度	浓度	浓度单位	标记	化合物名
23	20.894	2484	237	0.000		V	
24	21.401	5412	381	0.000			
25	22.482	75308	7902	0.000	mg/L		RT:22.482
26	23.583	404162	35871	0.000	mg/L		RT:23.583
27	24.330	2469	178	0.000		V	
28	25.317	8979	863	0.000		V	
29	25.663	93161	15141	0.000	mg/L	SV	RT:25.663
30	26.987	1508	142	0.000		TV	
31	27.373	1339	128	0.000		TV	
32	28.530	2511	152	0.000		TV	
33	28.900	1085	66	0.000		TV	
34	29.288	1316	82	0.000		TV	
35	30.000	1964	235	0.000			
36	30.530	2720	250	0.000			
37	31.297	3500	449	0.000		V	
38	31.691	375911	50385	0.000	mg/L	SV	RT:31.691
39	33.682	5813	708	0.000			
40	34.224	2294	213	0.000			
41	34.500	6040	523	0.000		V	
42	34.947	9389	763	0.000		V	
43	35.444	3379	374	0.000		V	
44	36.322	3113	328	0.000			
45	37.020	1681	80	0.000			
46	37.415	3403	360	0.000		V	
47	38.021	2570	235	0.000		V	
48	39.658	1170	91	0.000		V	
49	40.259	2120	218	0.000		V	
50	40.547	1321	170	0.000		V	
51	40.880	3316	234	0.000		V	
52	41.732	2799	259	0.000			
53	42.162	22042	1336	0.000		SV	
54	43.277	5592	542	0.000			
55	43.437	2726	529	0.000		V	
56	43.575	9486	678	0.000		V	
57	43.850	3249	371	0.000		V	
58	43.964	3597	365	0.000		V	
59	44.126	2076	326	0.000		V	
60	44.283	1135	197	0.000		V	
61	44.394	2510	274	0.000		V	
62	44.915	1542	141	0.000		V	
63	45.571	6501	458	0.000			
64	46.793	18200	1140	0.000			
65	50.057	7549	157	0.000			
总计		2173015	206354				
