# Supplementary material for: Underlying Mechanism and Active Ingredients of Tianma Gouteng Acting on Cerebral Infarction as Determined via Network Pharmacology Analysis Combined With Experimental Validation
Source: Front Pharmacol. 2021 Nov 16;12:760503. doi: 10.3389/fphar.2021.760503 (PMC8635202; doi:10.3389/fphar.2021.760503)
Supplement: Supplementary file 2 [file DataSheet1.zip › original data/HPLC/TMGT.PDF]

SHIMADZU LabSolutions

分析报告

<样品信息>

样品名 :
样品ID :
数据文件名 : 样品4.lcd
方法文件名 : 2C乙腈方法68min-0714.lcm
批处理文件名 : 0714.lcb
样品瓶号 : 1-12　　样品类型 : 未知
进样体积 : 10 uL
分析日期 : 2021/7/14 21:26:28　　分析者 : System Administrator
处理日期 : 2021/7/14 22:34:35　　处理者 : System Administrator

<色谱图>

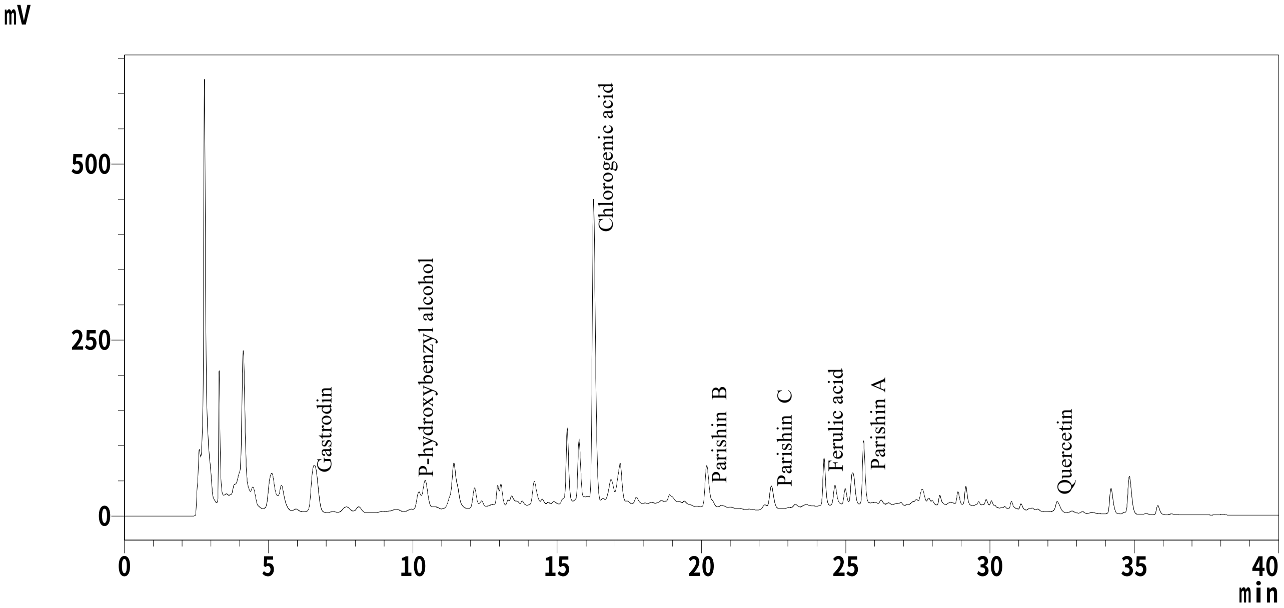

<峰表>

检测器A 254nm

峰号	保留时间	面积	高度	浓度	浓度单位	标记	化合物名
1	2.598	655541	94271	0.000			
2	2.776	4623088	620305	0.000		V	
3	3.288	1039395	205730	0.000		V	
4	3.544	319781	31478	0.000		V	
5	4.120	3320384	234815	0.000		V	
6	4.453	619150	41146	0.000		V	
7	5.111	1110884	60761	0.000		V	
8	5.447	677423	43601	0.000		V	
9	5.948	197969	9806	0.000		V	
10	6.589	1313473	72159	0.000	mg/L	V	RT:6.589
11	7.237	117288	6169	0.000		V	
12	7.698	296709	12802	0.000		V	
13	8.124	246112	12965	0.000		V	
14	8.952	116432	6430	0.000		V	
15	9.432	294184	9284	0.000		V	
16	9.959	152847	9555	0.000		V	
17	10.209	404105	34142	0.000	mg/L	V	RT:10.209
18	10.426	716176	50673	0.000		V	
19	10.767	170548	12976	0.000		V	
20	11.424	1523147	75048	0.000		V	
21	12.138	513223	39631	0.000		V	
22	12.388	265952	21516	0.000		V	

峰号	保留时间	面积	高度	浓度	浓度单位	标记	化合物名
23	12.939	579562	43214	0.000		V	
24	13.052	394448	45299	0.000		V	
25	13.301	160690	22578	0.000		V	
26	13.425	506338	28338	0.000		V	
27	13.788	267267	21219	0.000		V	
28	14.208	760401	49211	0.000		V	
29	14.483	251409	23661	0.000		V	
30	14.688	183479	19371	0.000		V	
31	14.884	315687	20354	0.000		V	
32	15.351	1254125	123577	0.000		V	
33	15.759	1083314	106798	0.000		V	
34	15.949	122335	27302	0.000		V	
35	16.060	192408	27907	0.000		V	
36	16.263	3863236	449946	0.000	mg/L	V	RT:16.263
37	16.585	238109	24808	0.000		V	
38	16.866	777535	51626	0.000		V	
39	17.184	1055170	74501	0.000		V	
40	17.432	234410	21116	0.000		V	
41	17.746	469334	26549	0.000		V	
42	18.048	196447	18380	0.000		V	
43	18.278	286175	19161	0.000		V	
44	18.611	369806	21795	0.000		V	
45	18.895	665930	29846	0.000		V	
46	19.228	188967	20731	0.000		V	
47	19.408	456777	20900	0.000		V	
48	19.849	160684	13836	0.000		V	
49	20.184	1071036	71461	0.000	mg/L	V	RT:20.184
50	20.689	284580	14726	0.000		V	
51	21.012	253515	12387	0.000		V	
52	21.317	136437	10369	0.000		V	
53	21.650	213558	9397	0.000		V	
54	22.196	245148	15638	0.000		V	
55	22.423	580432	42362	0.000	mg/L	V	RT:22.423
56	22.810	110286	10569	0.000		V	
57	23.039	145768	11704	0.000		V	
58	23.257	270333	16087	0.000		V	
59	23.643	490218	15918	0.000	mg/L	V	RT:23.643
60	24.250	839942	81887	0.000		V	
61	24.627	602713	43373	0.000		V	
62	24.983	400773	38491	0.000		V	
63	25.238	792883	60975	0.000		V	
64	25.620	1002211	106253	0.000	mg/L	V	RT:25.620
65	25.938	150218	19157	0.000		V	
66	26.038	128377	18720	0.000		V	
67	26.225	299309	22377	0.000		V	
68	26.485	256892	18375	0.000		V	
69	26.746	150897	17440	0.000		V	
70	26.913	274159	18847	0.000		V	
71	27.137	132924	16200	0.000		V	
72	27.455	400868	23301	0.000		V	
73	27.651	499915	37679	0.000		V	
74	27.879	217827	25115	0.000		V	
75	27.996	217695	21310	0.000		V	
76	28.261	374311	29113	0.000		V	
77	28.622	224618	19316	0.000		V	
78	28.700	108325	18726	0.000		V	
79	28.891	365270	33943	0.000		V	
80	29.164	520025	41636	0.000		V	
81	29.605	273991	19934	0.000		V	
82	29.871	296081	22777	0.000		V	
83	30.050	315648	21189	0.000		V	
84	30.508	211472	13005	0.000		V	

峰号	保留时间	面积	高度	浓度	浓度单位	标记	化合物名
85	30.743	274667	20319	0.000		V	
86	31.077	191957	16903	0.000		V	
87	31.655	172831	9435	0.000	mg/L	V	RT:31.655
88	32.328	318898	20112	0.000		V	
89	32.845	132212	6535	0.000		V	
90	34.194	394547	38693	0.000		V	
91	34.831	637573	55733	0.000		SV	
92	35.817	142985	14516	0.000		V	
总计		48852179	4259292				
