# Supplementary figures and images for: Underlying Mechanism and Active Ingredients of Tianma Gouteng Acting on Cerebral Infarction as Determined via Network Pharmacology Analysis Combined With Experimental Validation
Source: Front Pharmacol. 2021 Nov 16;12:760503. doi: 10.3389/fphar.2021.760503 (PMC8635202; doi:10.3389/fphar.2021.760503)

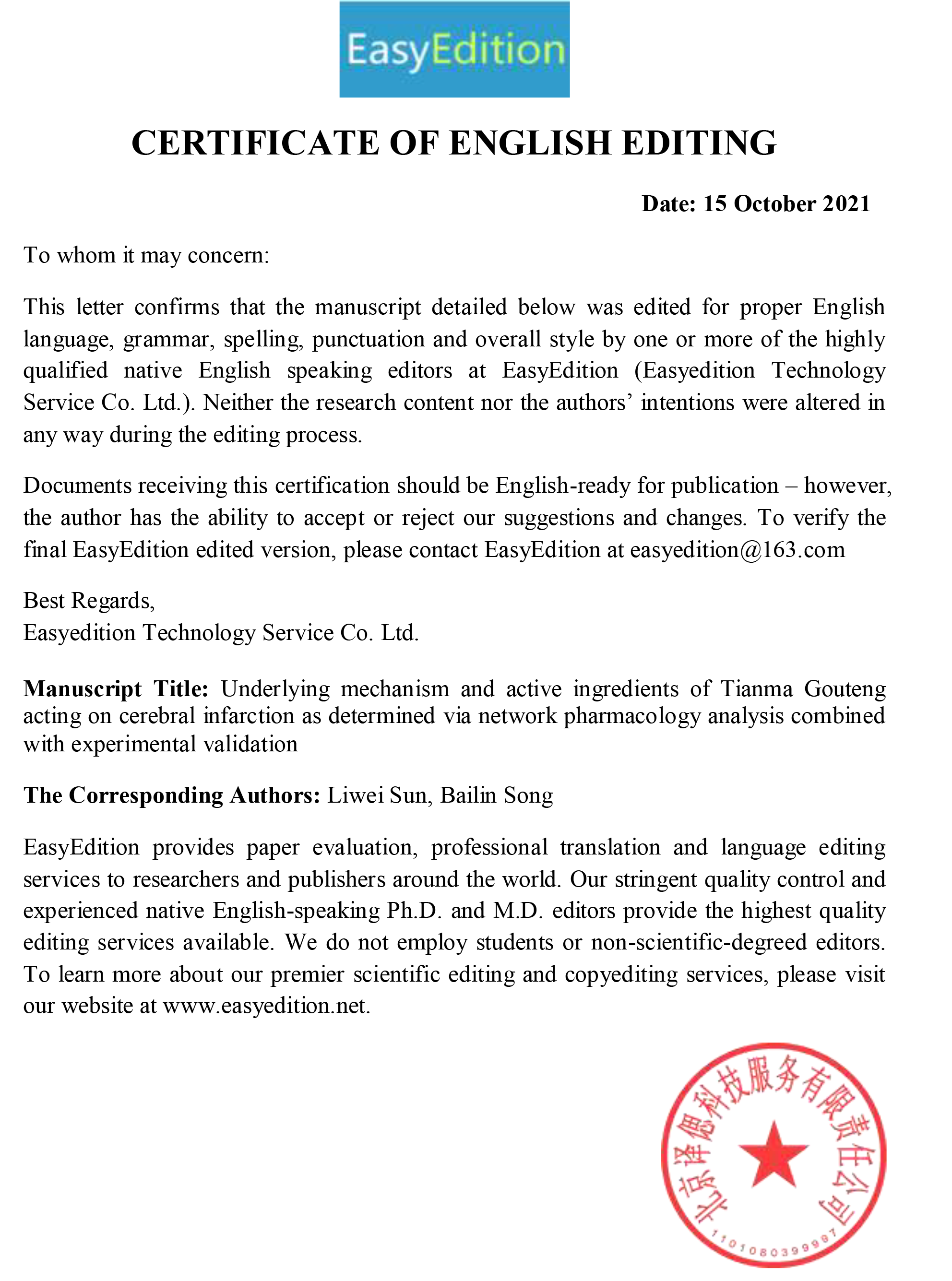

Supplement: Supplementary file 2 [file DataSheet1.zip › original data/English Editing Certification.tif]

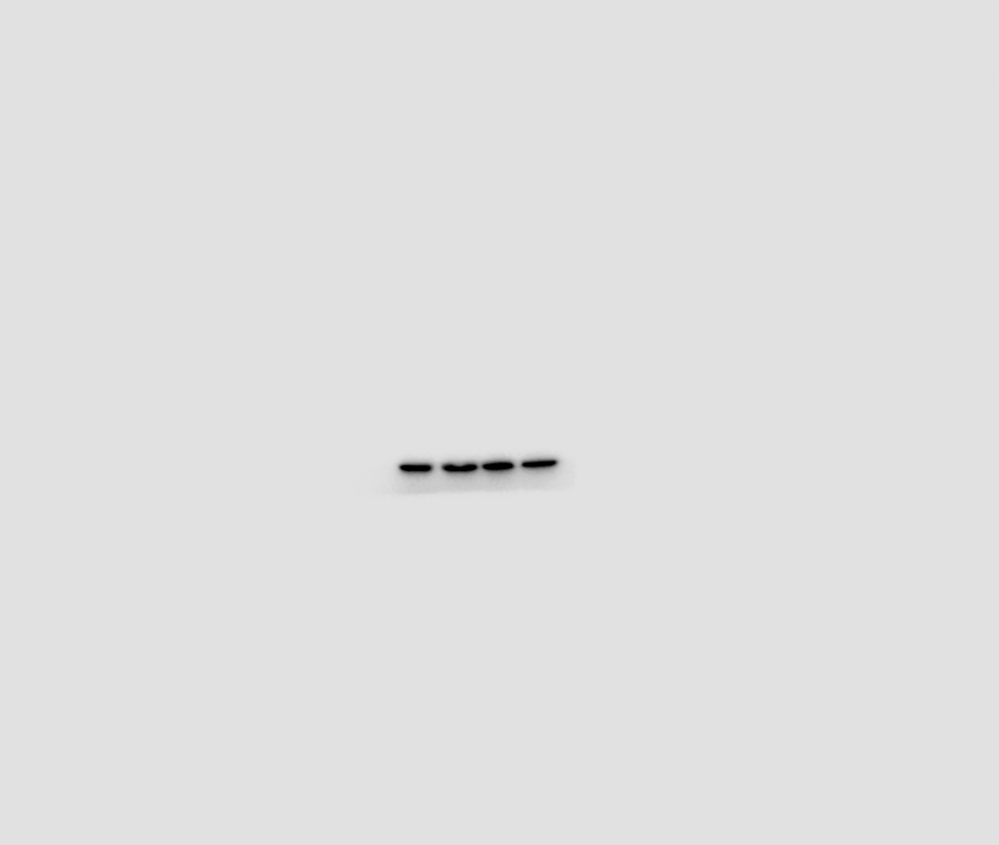

Supplement: Supplementary file 2 [file DataSheet1.zip › original data/TMGT-WB-TIFF/ACTIN-BV2-4-TMGT.tif]

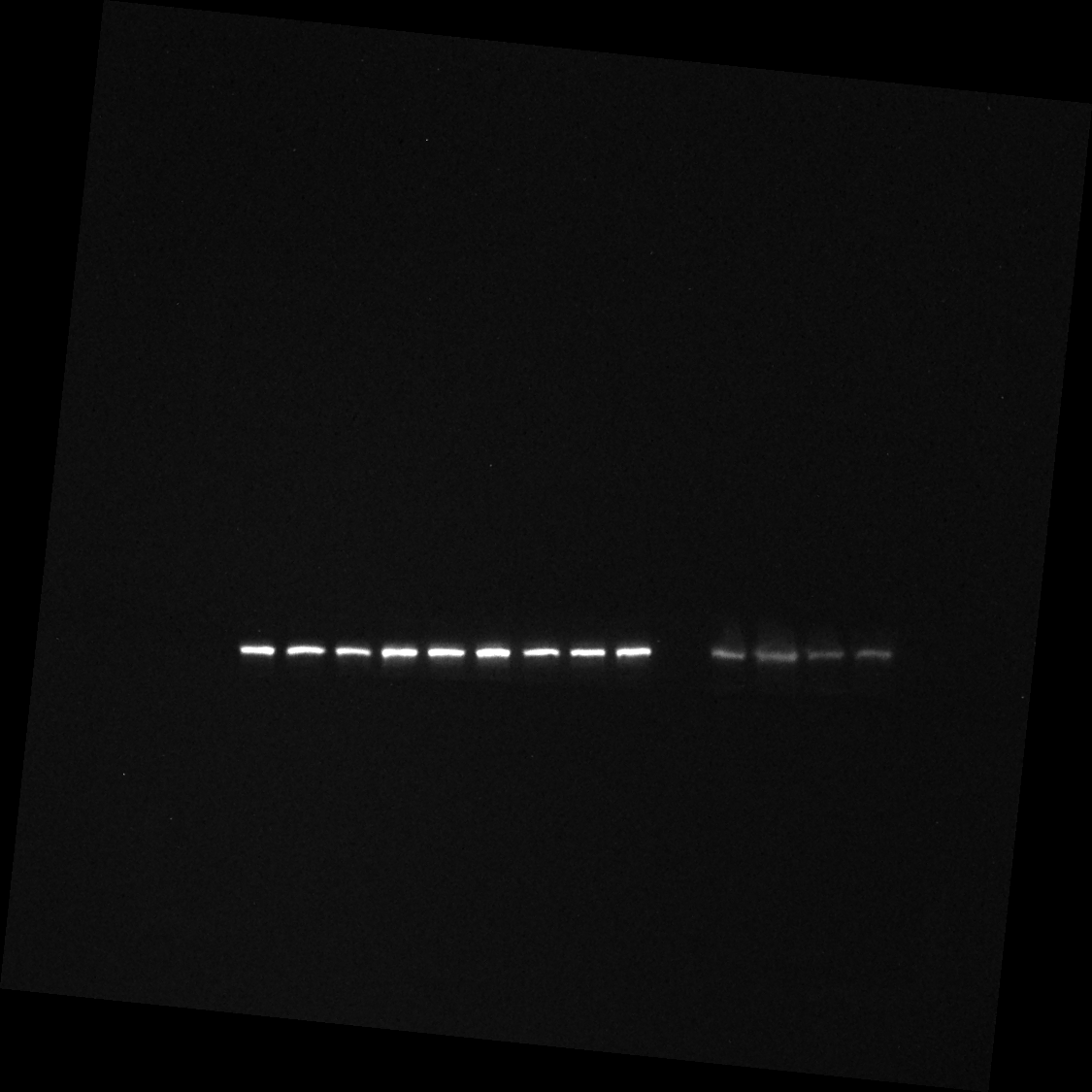

Supplement: Supplementary file 2 [file DataSheet1.zip › original data/TMGT-WB-TIFF/BV2-4-HIF-1A-TMGT.tif]

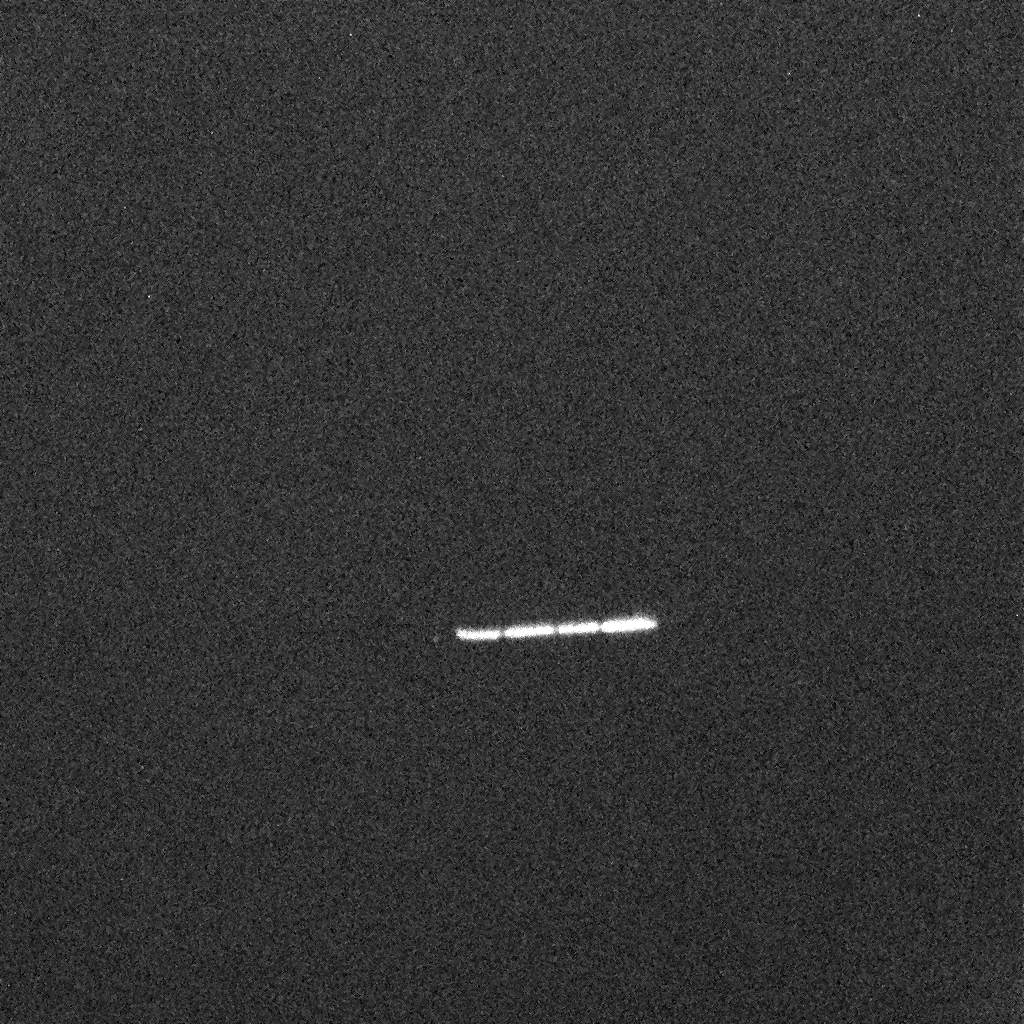

Supplement: Supplementary file 2 [file DataSheet1.zip › original data/TMGT-WB-TIFF/BV2-PPARG-TMGT-4.tif]

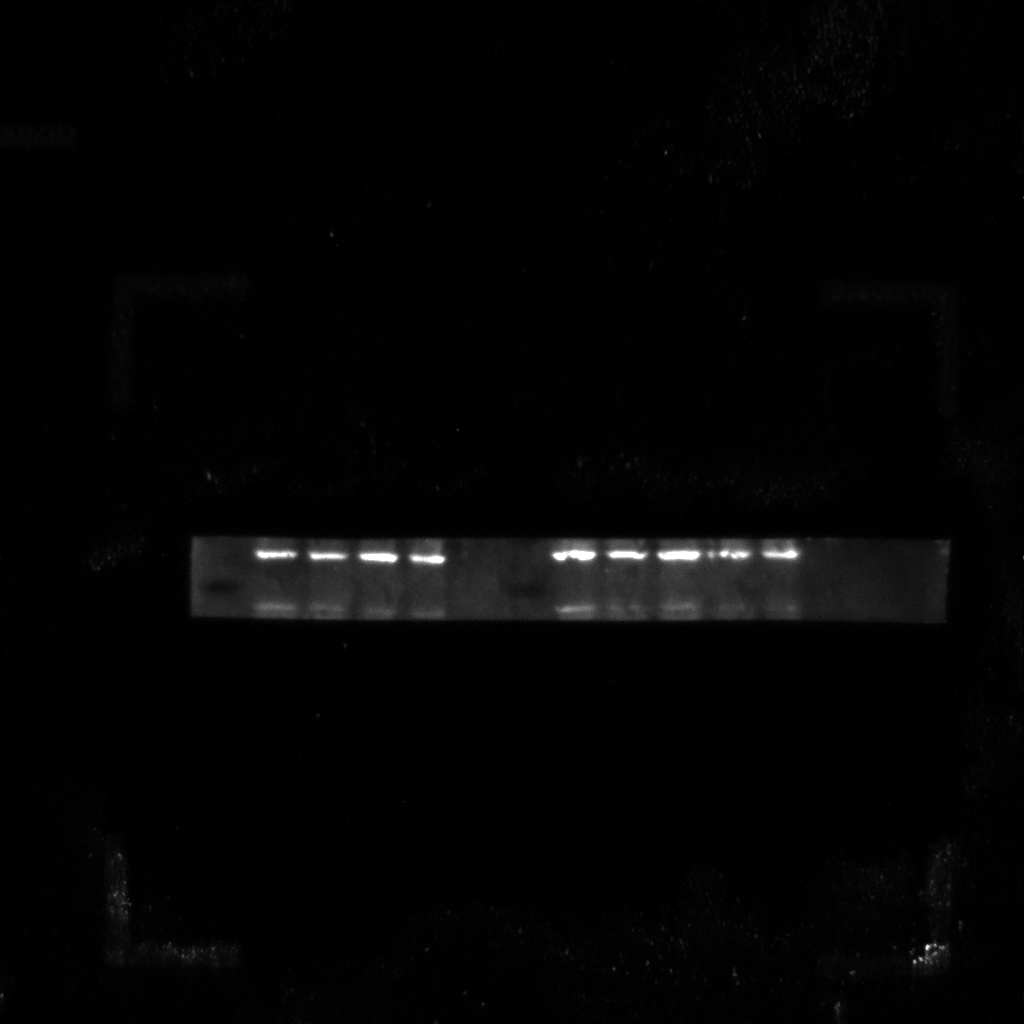

Supplement: Supplementary file 2 [file DataSheet1.zip › original data/TMGT-WB-TIFF/hif-a-pc12-4tmgt.tif]

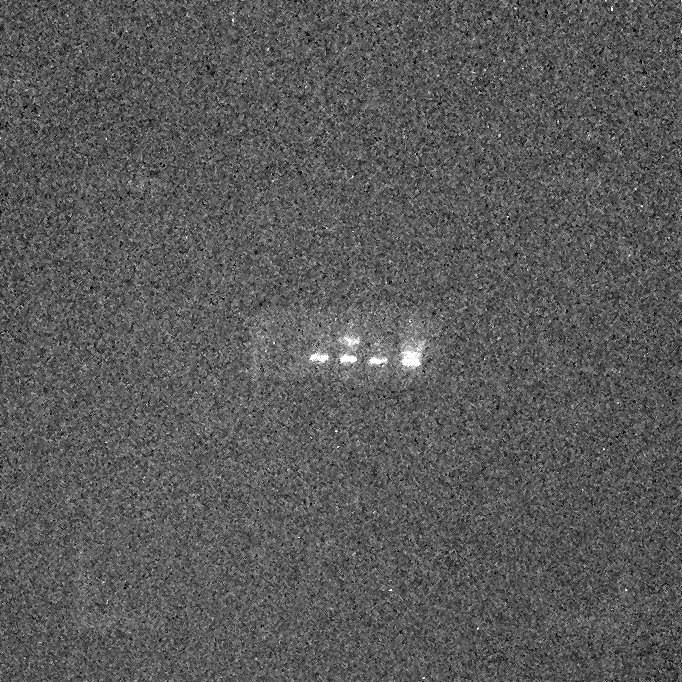

Supplement: Supplementary file 2 [file DataSheet1.zip › original data/TMGT-WB-TIFF/IKBA-BV2-4-TMGT.tif]

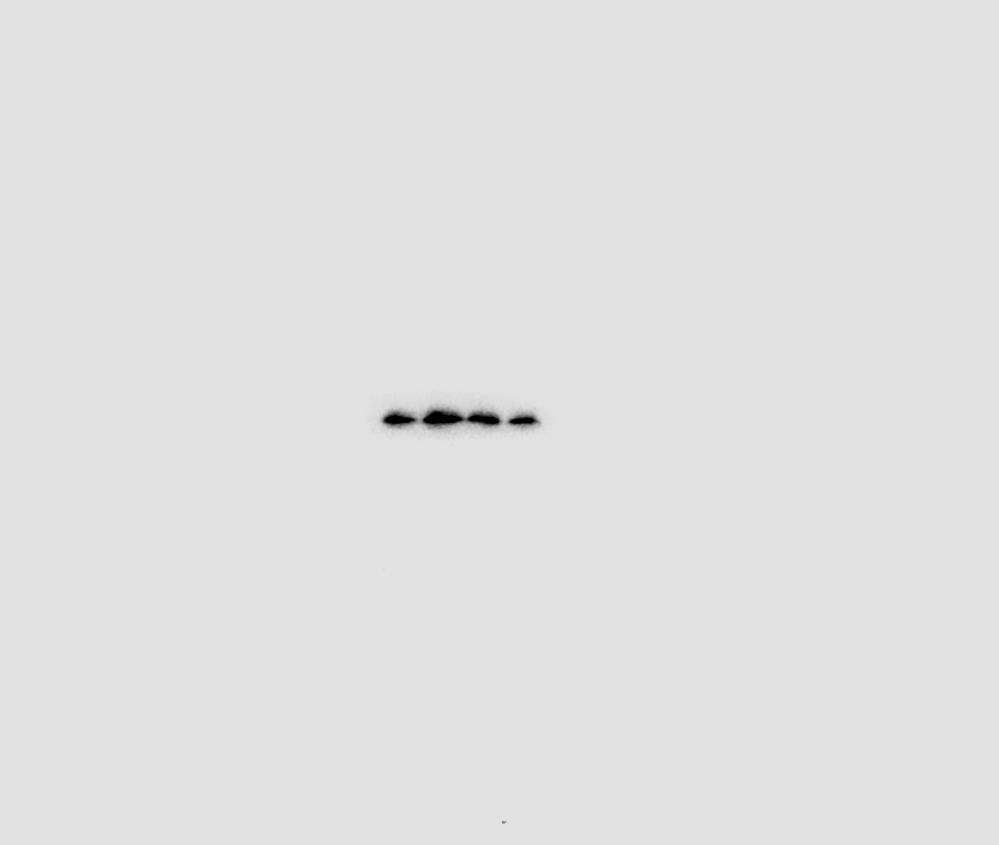

Supplement: Supplementary file 2 [file DataSheet1.zip › original data/TMGT-WB-TIFF/P-IKBA-PC12-4-TMGT.tif]

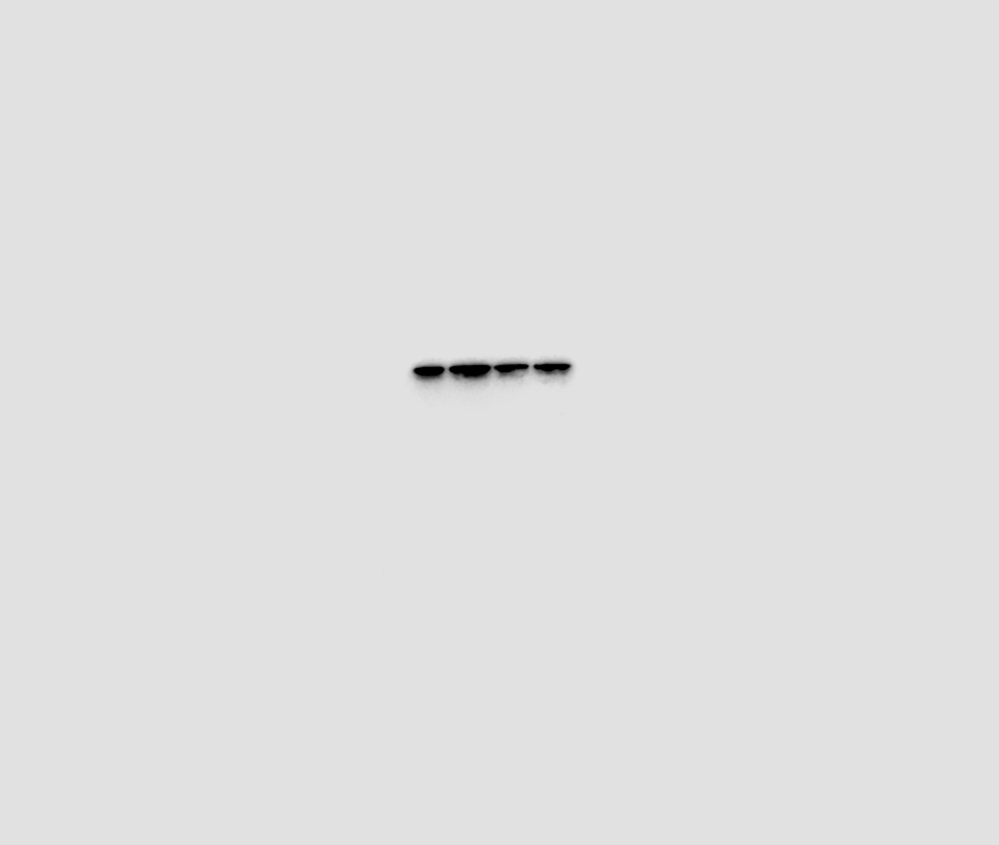

Supplement: Supplementary file 2 [file DataSheet1.zip › original data/TMGT-WB-TIFF/P-P65-BV2-4-TMGT.tif]

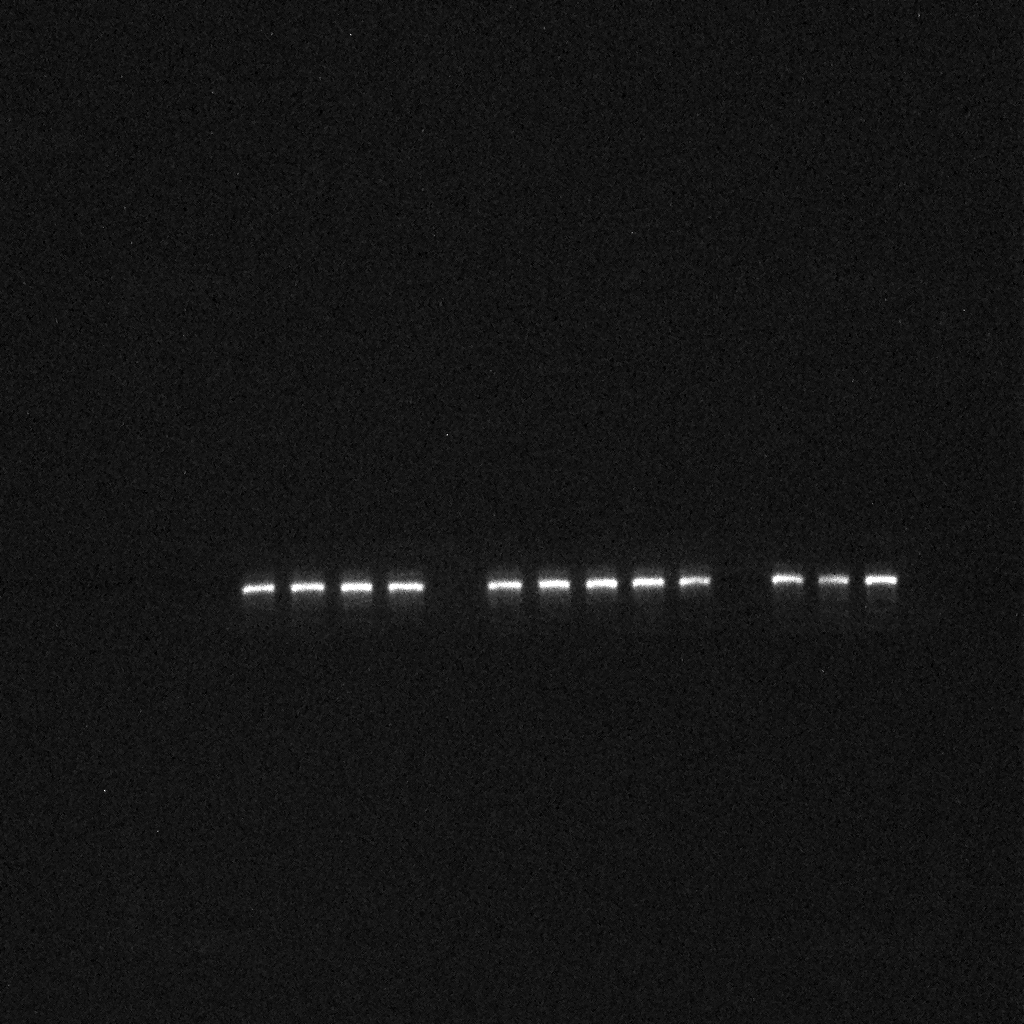

Supplement: Supplementary file 2 [file DataSheet1.zip › original data/TMGT-WB-TIFF/p65-middle 5-pc12-tmgt.tif]

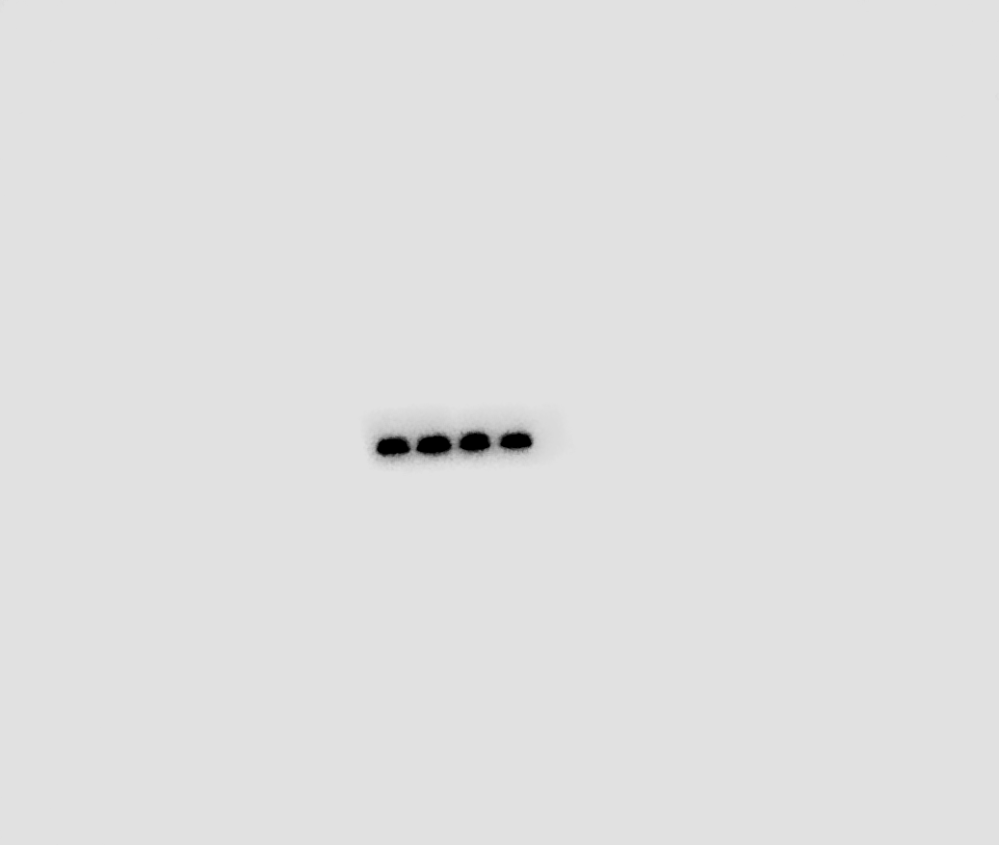

Supplement: Supplementary file 2 [file DataSheet1.zip › original data/TMGT-WB-TIFF/P65-PC12-4-TMGT.tif]

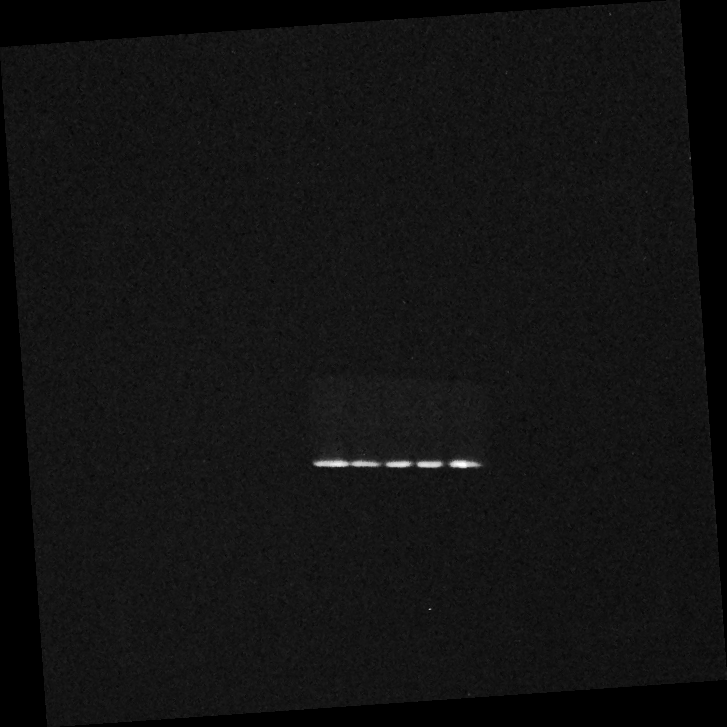

Supplement: Supplementary file 2 [file DataSheet1.zip › original data/TMGT-WB-TIFF/PC12-5-IKB-tmgt.tif]

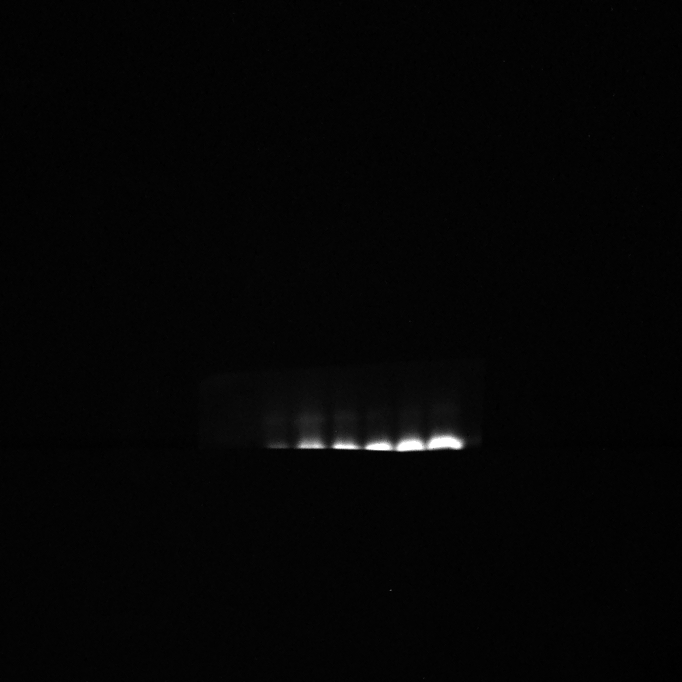

Supplement: Supplementary file 2 [file DataSheet1.zip › original data/TMGT-WB-TIFF/ppar-bv2-5-tmgt.tif]

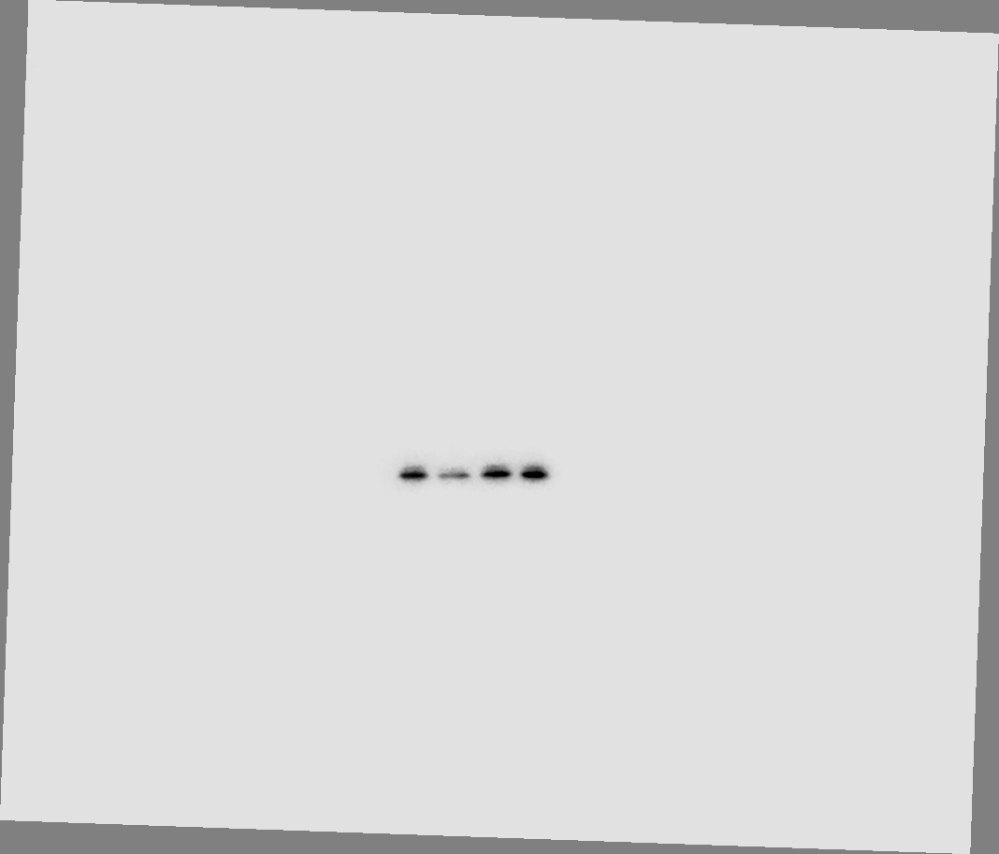

Supplement: Supplementary file 2 [file DataSheet1.zip › original data/TMGT-WB-TIFF/PPAR-PC12-4-TMGT.tif]

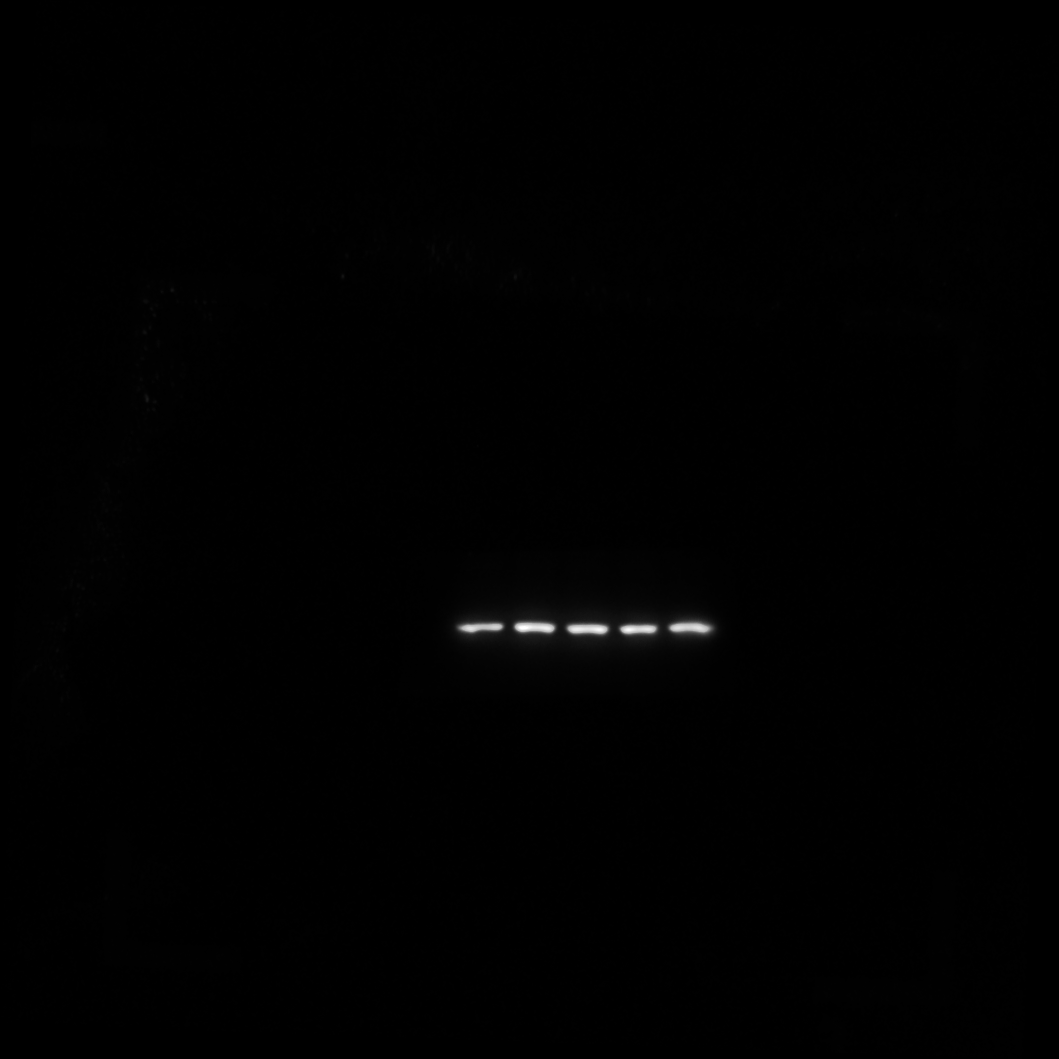

Supplement: Supplementary file 2 [file DataSheet1.zip › original data/TMGT-WB-TIFF/TM-GTPP65-5-PC12.tif]

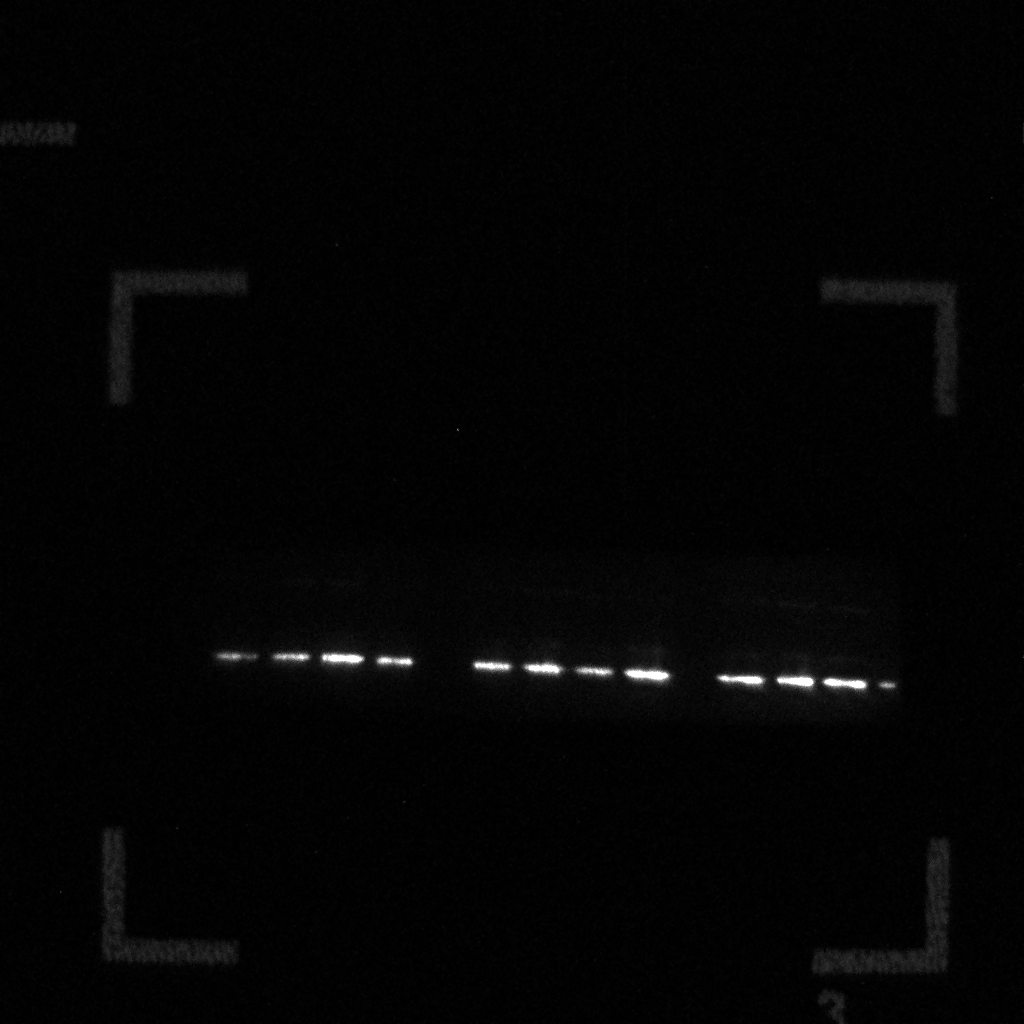

Supplement: Supplementary file 2 [file DataSheet1.zip › original data/TMGT-WB-TIFF/TMGT-4-PC12-IKB.tif]

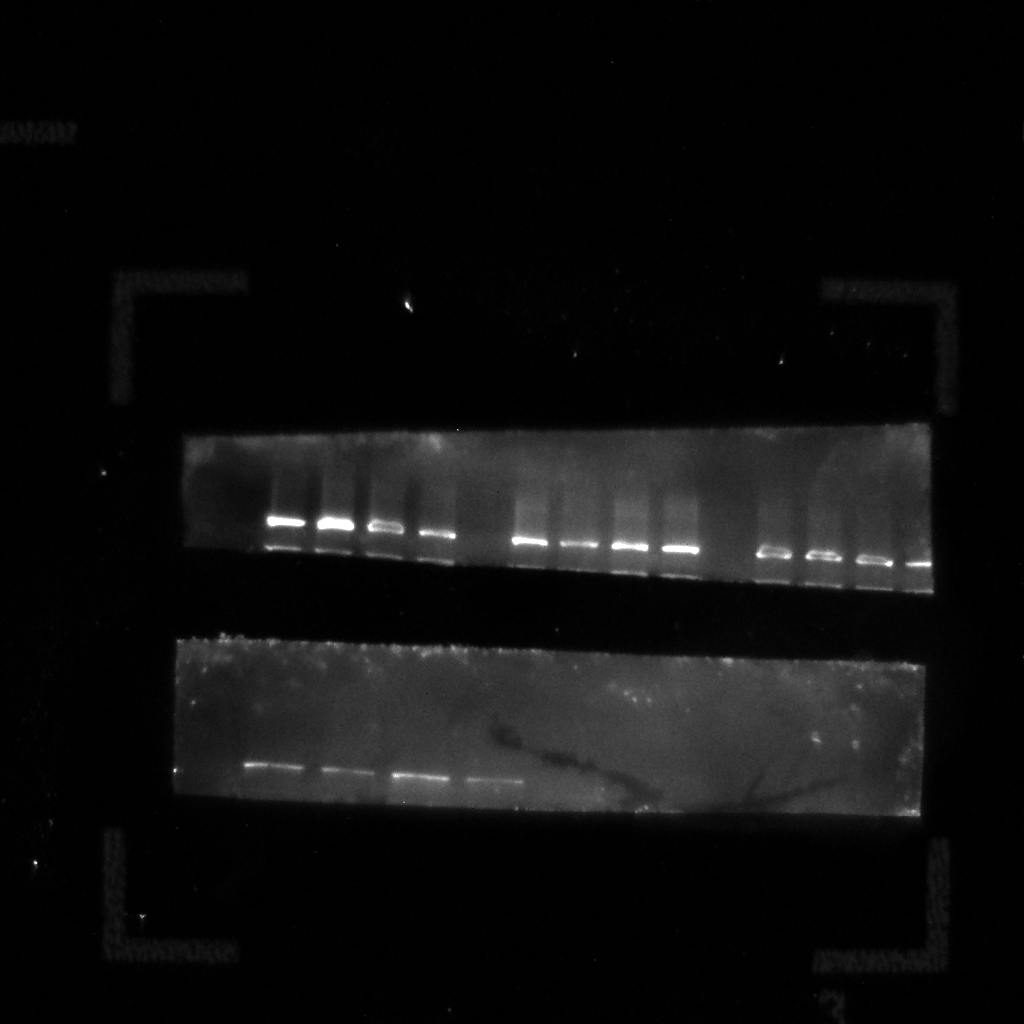

Supplement: Supplementary file 2 [file DataSheet1.zip › original data/TMGT-WB-TIFF/TMGT-4-PC12-PP65.tif]

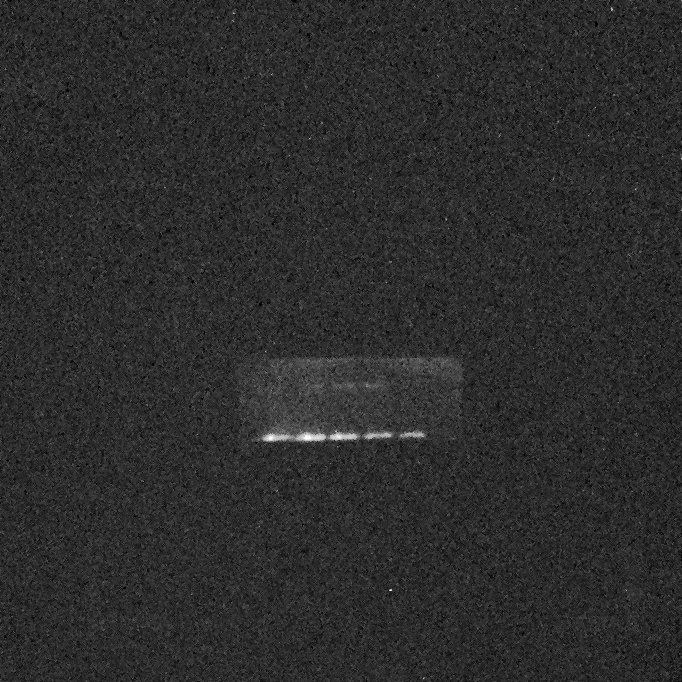

Supplement: Supplementary file 2 [file DataSheet1.zip › original data/TMGT-WB-TIFF/TMGT-5-BV2-HIF1A.tif]

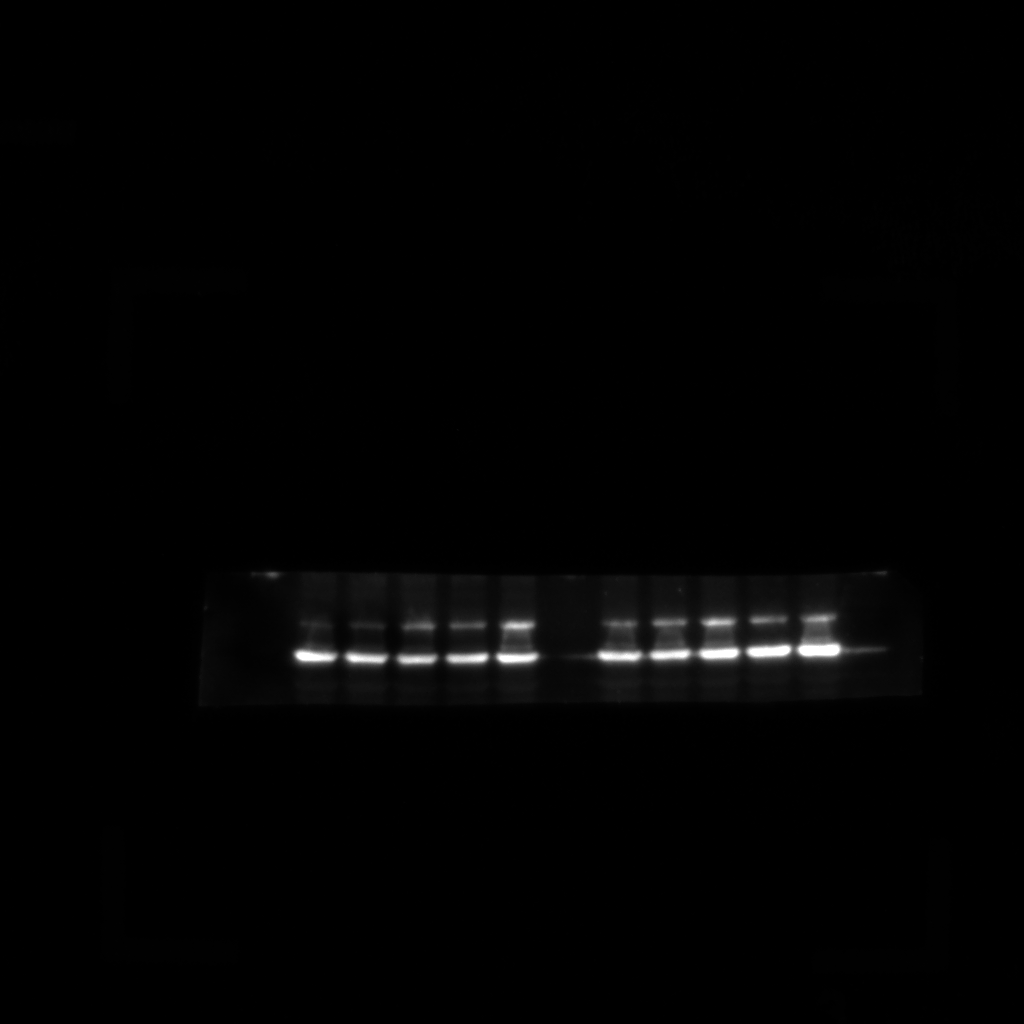

Supplement: Supplementary file 2 [file DataSheet1.zip › original data/TMGT-WB-TIFF/TMGT-5-PC12 ACTIN.tif]

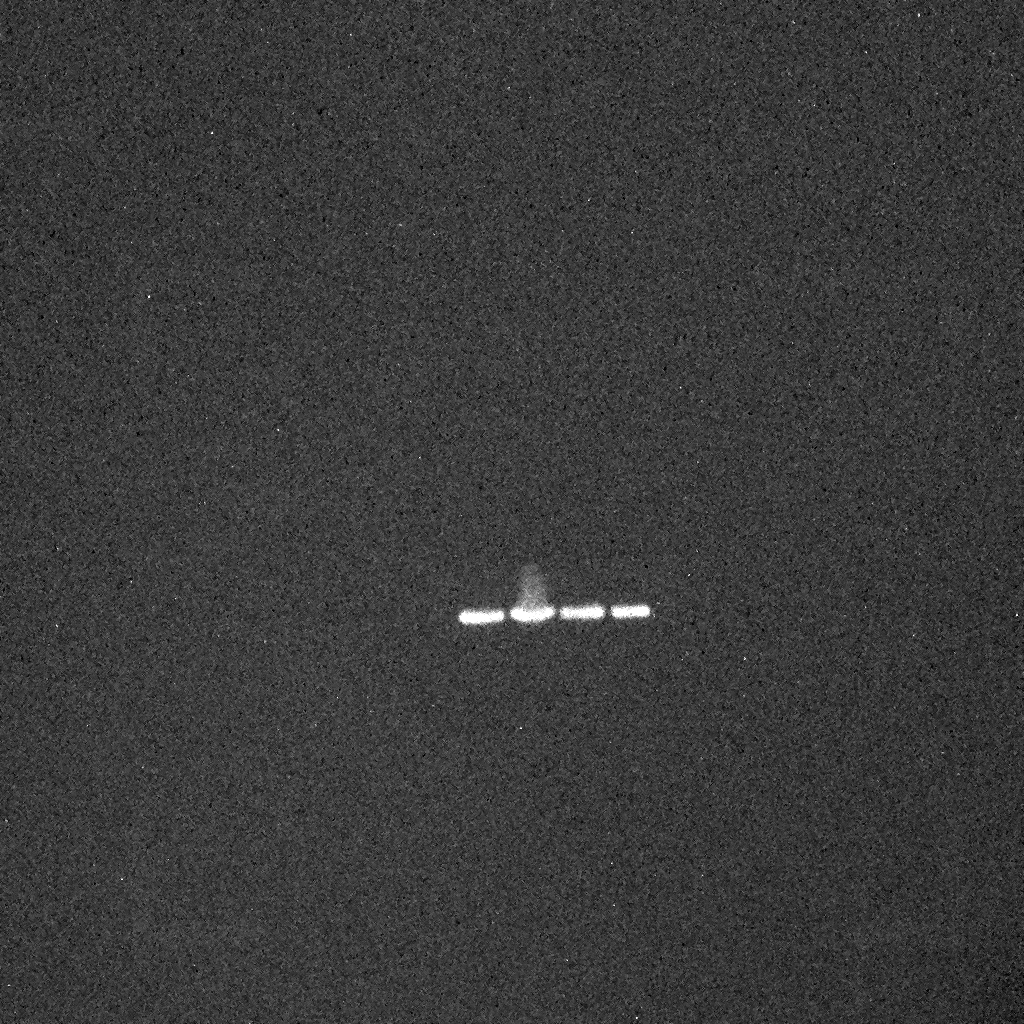

Supplement: Supplementary file 2 [file DataSheet1.zip › original data/TMGT-WB-TIFF/TMGT-BV2--4-P65.tif]

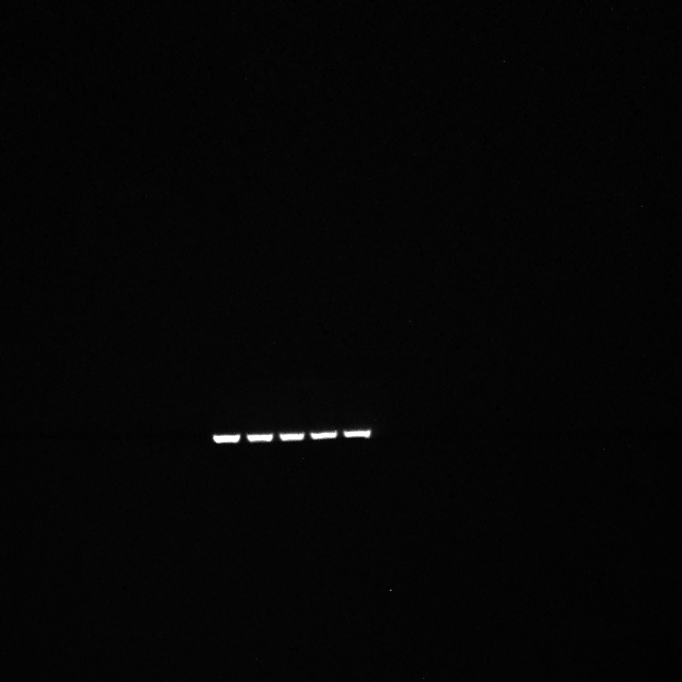

Supplement: Supplementary file 2 [file DataSheet1.zip › original data/TMGT-WB-TIFF/TMGT-BV2-5-ACTIN.tif]

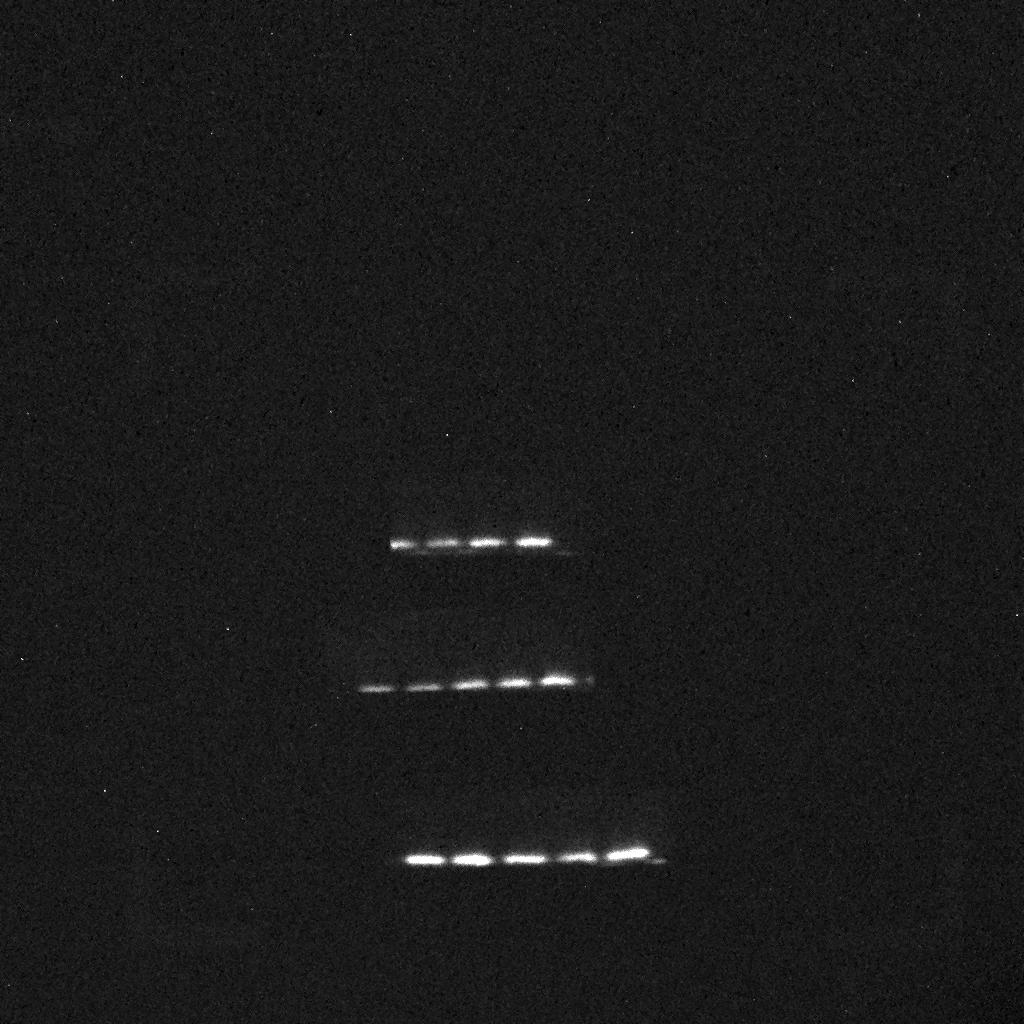

Supplement: Supplementary file 2 [file DataSheet1.zip › original data/TMGT-WB-TIFF/tmgt-bv2-ikb-5.tif]

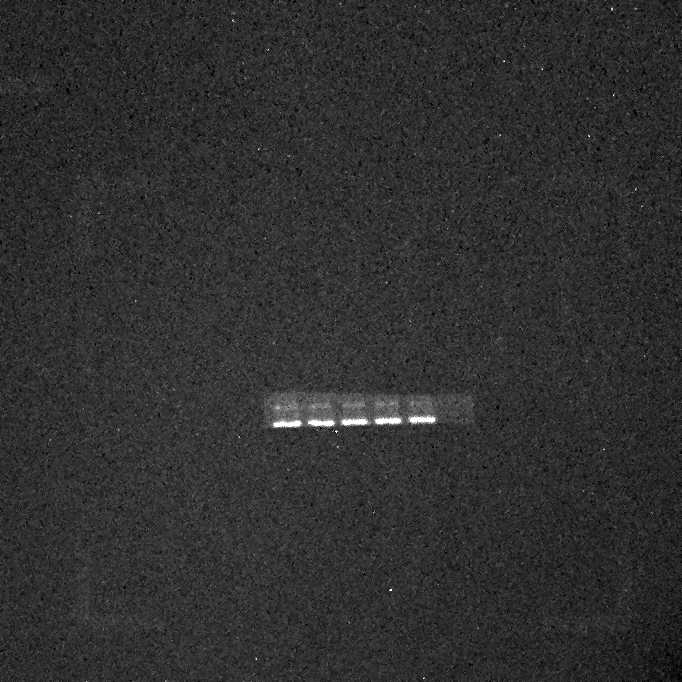

Supplement: Supplementary file 2 [file DataSheet1.zip › original data/TMGT-WB-TIFF/tmgt-bv2-p65-5.tif]

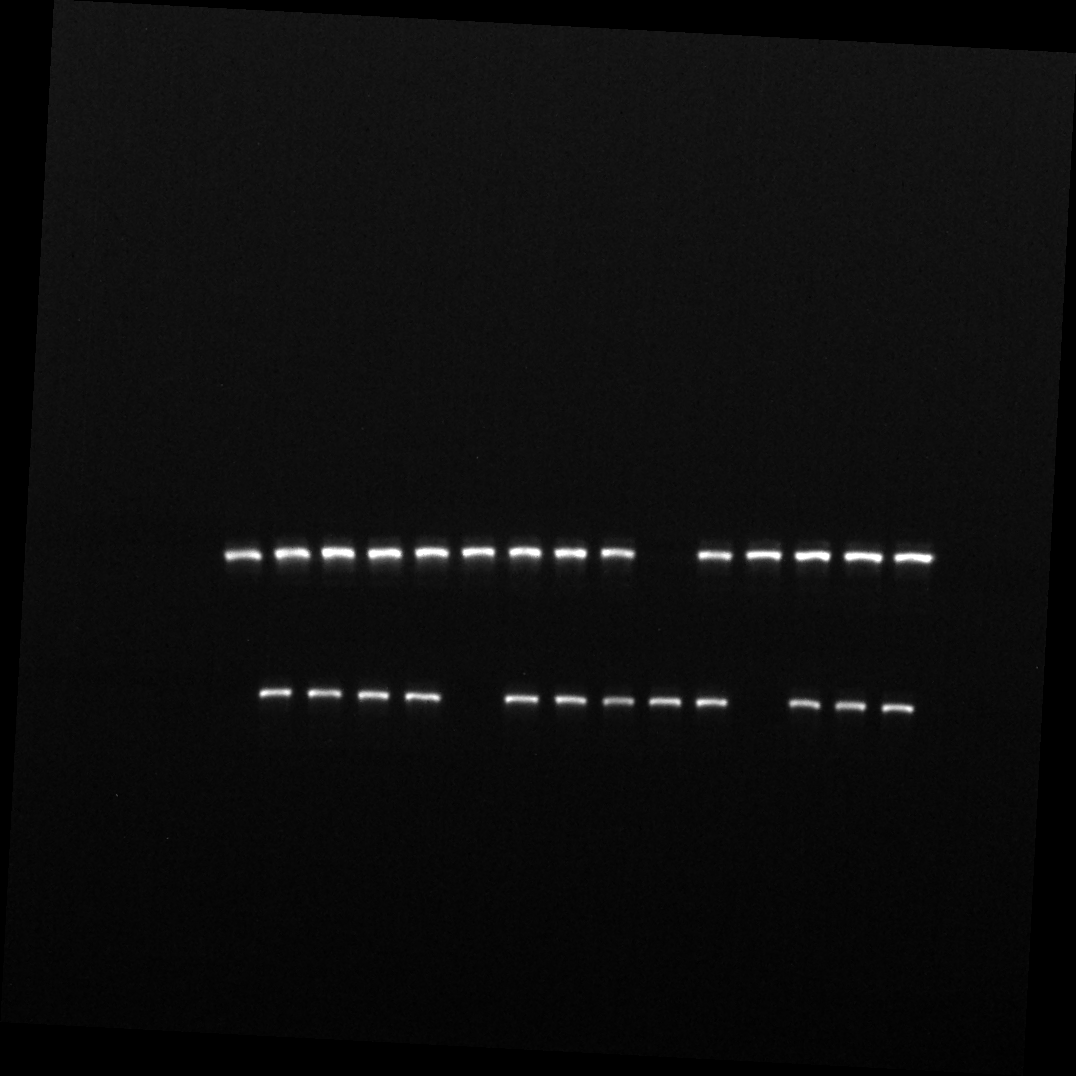

Supplement: Supplementary file 2 [file DataSheet1.zip › original data/TMGT-WB-TIFF/TMGT-PC12-4-ACTIN.tif]

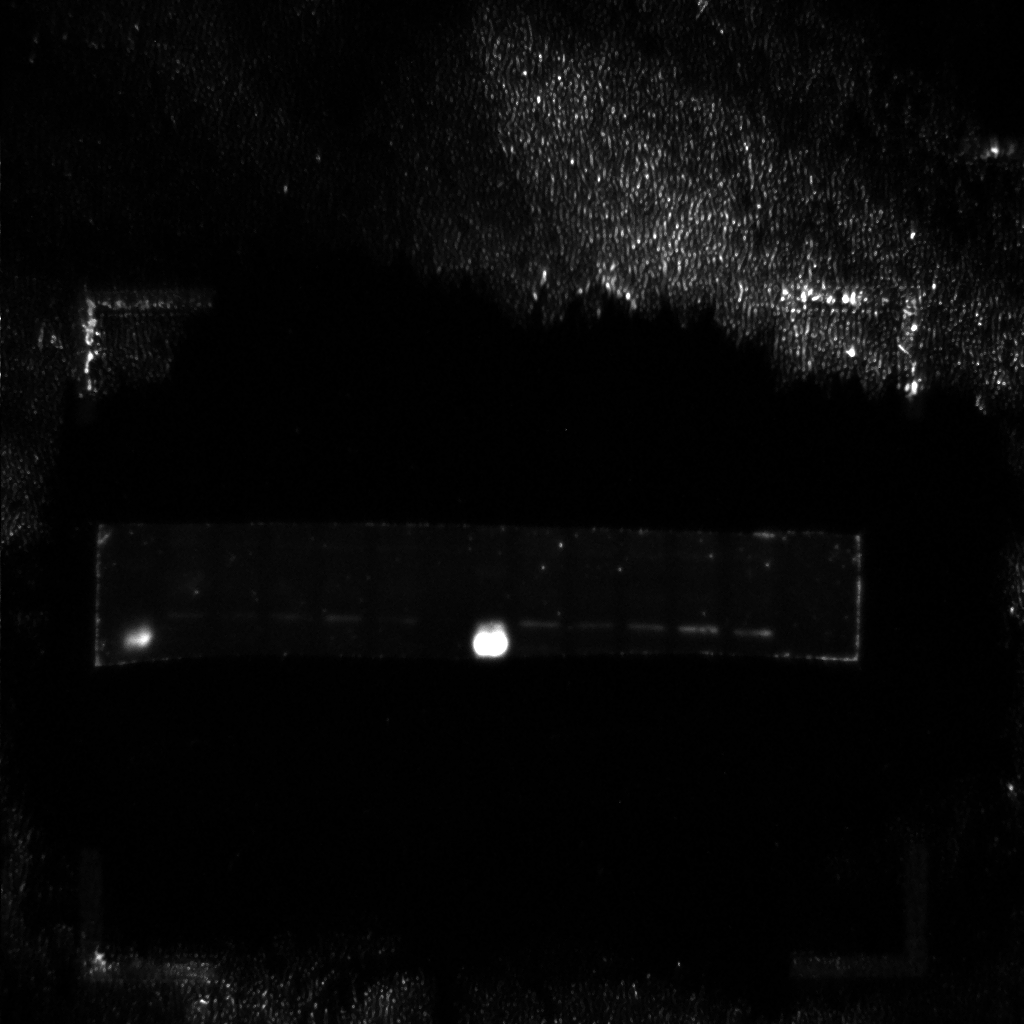

Supplement: Supplementary file 2 [file DataSheet1.zip › original data/TMGT-WB-TIFF/TMGT-PC12-5-PIKB.tif]

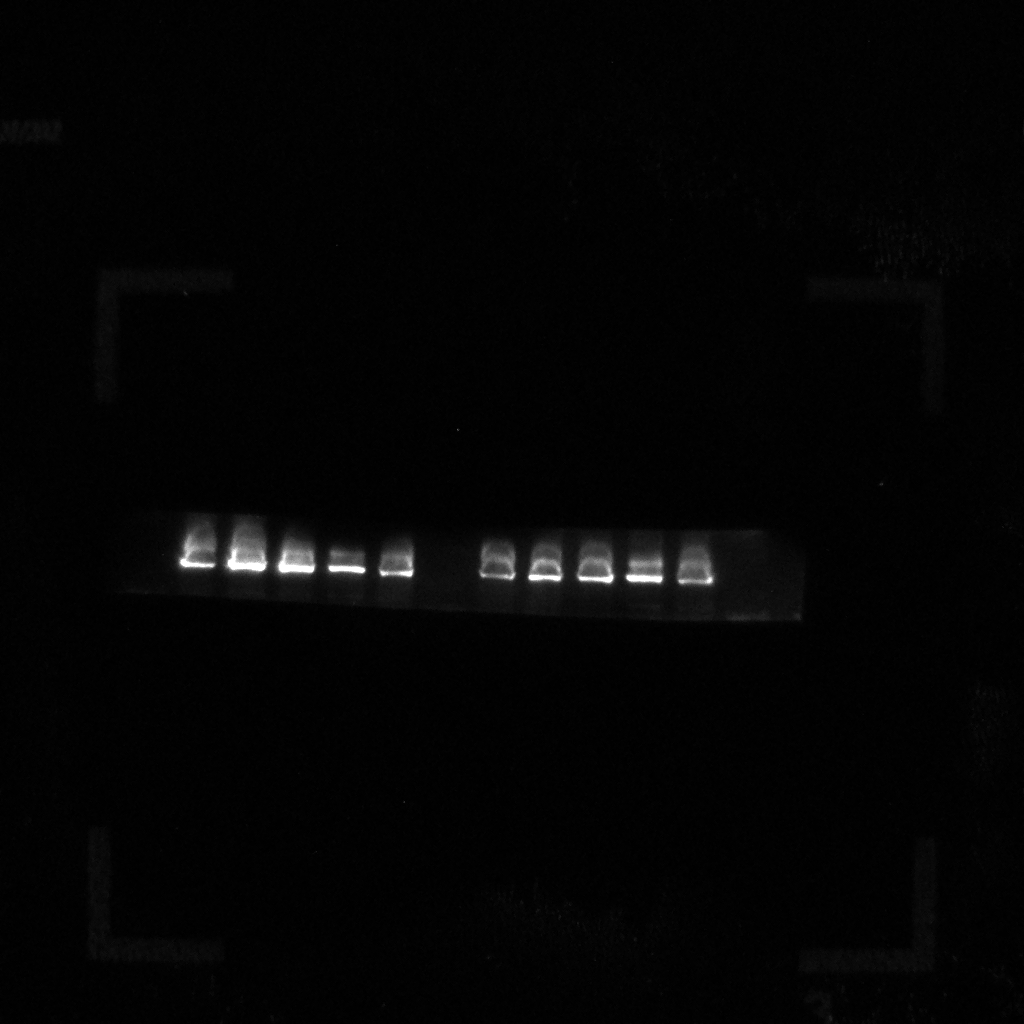

Supplement: Supplementary file 2 [file DataSheet1.zip › original data/TMGT-WB-TIFF/TMGT-PC12-HIF1A-5.tif]

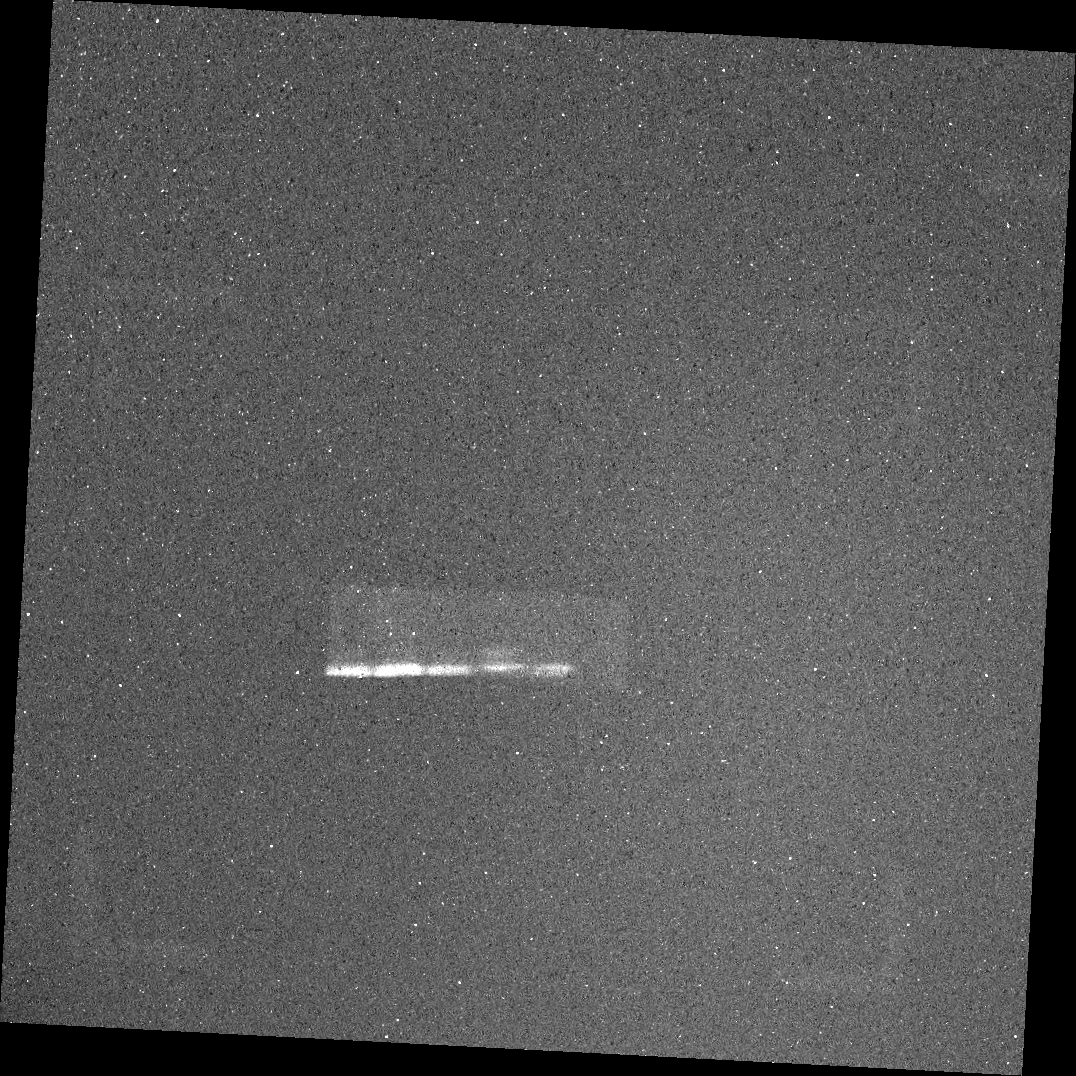

Supplement: Supplementary file 2 [file DataSheet1.zip › original data/TMGT-WB-TIFF/tmgt-pikb-bv2-5.tif]

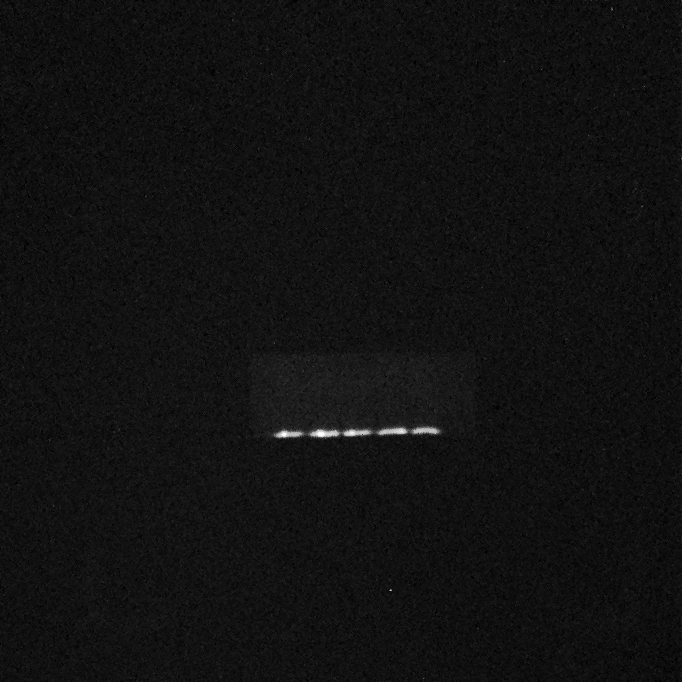

Supplement: Supplementary file 2 [file DataSheet1.zip › original data/TMGT-WB-TIFF/tmgt-pp65-5-bv2.tif]

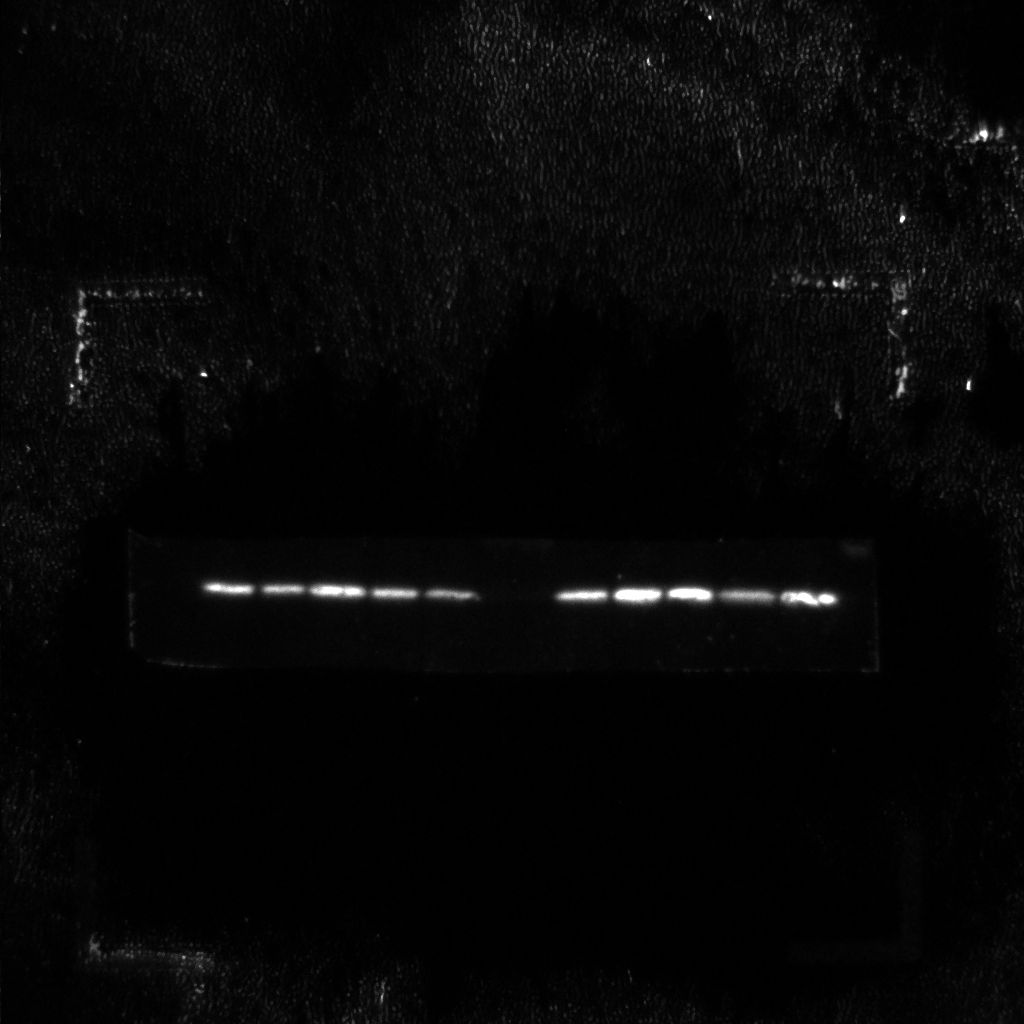

Supplement: Supplementary file 2 [file DataSheet1.zip › original data/TMGT-WB-TIFF/TMGT-pparg-pc12-5.tif]

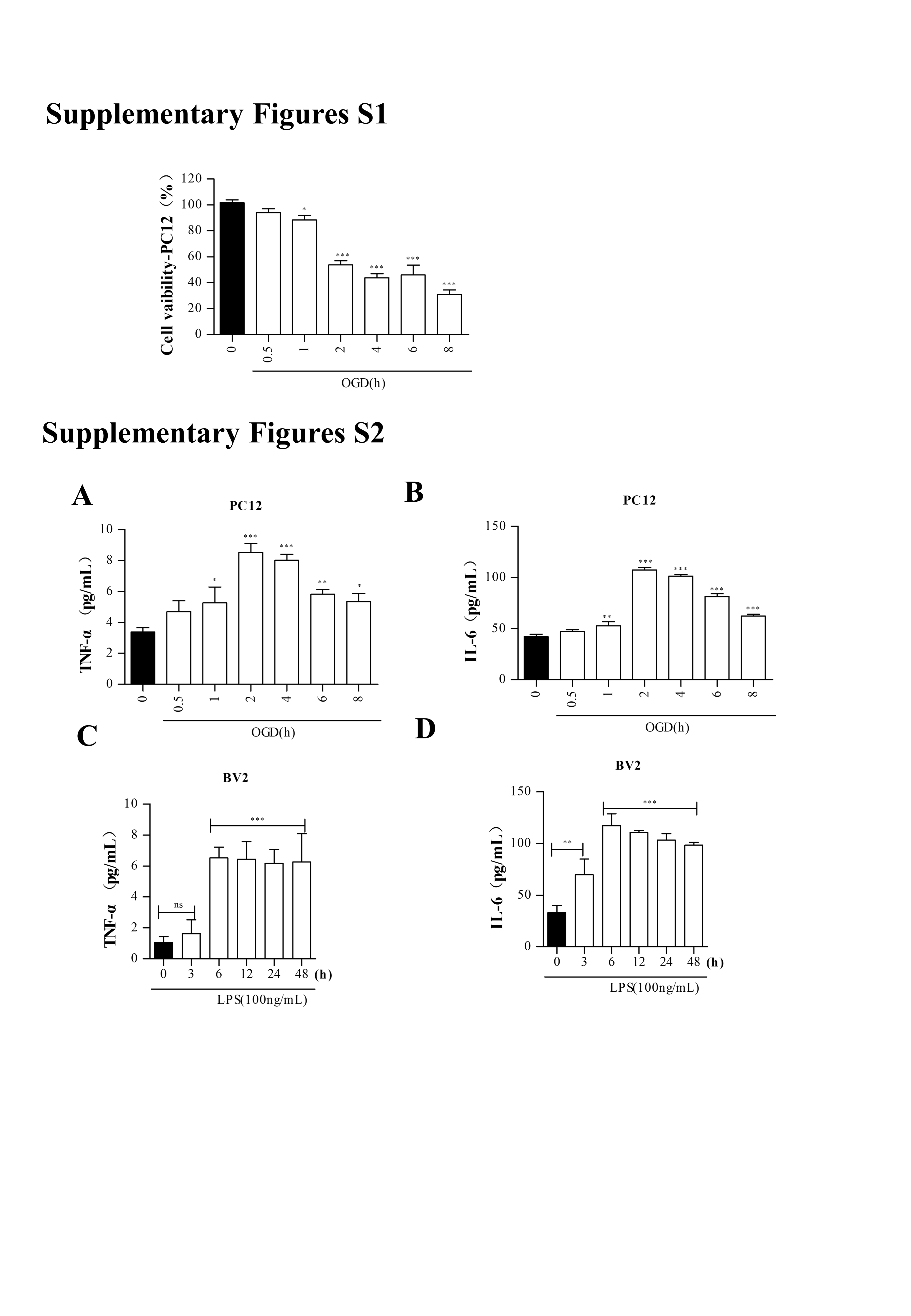

Supplement: Supplementary file 3 [file Image1.tif]
