# Supplementary material for: Underlying Mechanism and Active Ingredients of Tianma Gouteng Acting on Cerebral Infarction as Determined via Network Pharmacology Analysis Combined With Experimental Validation
Source: Front Pharmacol. 2021 Nov 16;12:760503. doi: 10.3389/fphar.2021.760503 (PMC8635202; doi:10.3389/fphar.2021.760503)
Supplement: Supplementary file 4 [file Table2.docx]

**Supplementary Table S2** Description of TM-GT active ingredients

| **Molecular ID** | **Compounds Name** |
| --- | --- |
| MOL005385 | Suffruticoside A |
| MOL005384 | Suchilactone |
| MOL001843 | P-Hydroxybenzaldehyde |
| MOL006927 | P-Hydroxybenzyl Alcohol |
| MOL011957 | M-Hydroxybenzoic Acid |
| MOL011455 | 20-Hexadecanoylingenol |
| MOL001965 | Dauricine |
| MOL007986 | Gastrodin |
| MOL000635 | Vanillin |
| MOL002647 | Vanillyl Alcohol |
| MOL000842 | Sucrose |
| MOL004650 | Vanillin Acetate |
| MOL000774 | Citronellal |
| MOL012237 | Daucosterol |
| MOL000358 | Beta-sitosterol |
| MOL000359 | Sitosterol |
| MOL000422 | Kaempferol |
| MOL000073 | Ent-Epicatechin |
| MOL008455 | 3-oxo-22α-hydroxyurs-12-en-27,28-dioc acid |
| MOL008456 | (3E,4R)-4-(1,3-benzodioxol-5-ylmethyl)-3- [(3,4,5-trimethoxyphenyl)methylidene] oxolan-2- one |
| MOL008457 | Tetrahydroalstonine |
| MOL008458 | Angustidine |
| MOL008460 | Geissoschizinc acid |
| MOL008463 | SMR000232338 |
| MOL008465 | (E)-16,17-Didehydro-17-methoxy-17,18-seco-3-beta-yohimban-16-carboxylic acid methylester |
| MOL008467 | Rhynchophylline A |
| MOL008468 | Methyl (E)-2-[(2S,3Z,12bS)-3-ethylidene-2,4,6,7,12,12b-hexahydro-1H-indolo [3,2-  h]quinolizin-2-yl]-3-methoxyprop-2-enoate |
| MOL008469 | Rhynchophylline |
| MOL008470 | SMR000232333 |
| MOL008471 | Isorhyncophylline |
| MOL008472 | HirsutasideA |
| MOL008473 | (E)-2-[(3S,6'S,7'S,8'aS)-6'-ethyl-2-keto-spiro[indoline-3,1'-indolizidine]-7'-yl]-3-methoxy- acrylic acid methyl ester |
| MOL008475 | Mitraphyllic acid |
| MOL008476 | HirsutasideB |
| MOL008477 | corynoxeine |
| MOL008478 | methyl (E)-2-[(2S,3R,12bS)-3-vinyl-1,2,3,4,6,7,12,12b-octahydroindolo[3,2-h] quinolizin-2-yl]-3-methoxy-prop-2-enoate |
| MOL008481 | (1'R,3S,4a'S,5a'S,10a'R)-1'-methyl-2-oxo-1',4a',5',5a',7',8',10',10a'-octahydrospiro [indoline-3,6'-pyrano[3,4-f]indolizine]-4'-carboxylic acid |
| MOL008482 | (2S,12bR)-methyl 2-((E)-1-oxobut-2-en-2-yl)-1,2,6,7,12,12b-hexahydroindolo [2,3-a]quinolizine-3-carboxylate |
| MOL008484 | Vincoside lactam_qt |
| MOL008485 | HirsutasideC |
| MOL008487 | Hirsutine |
| MOL008488 | Yohimbine |
| MOL008489 | Delta(sup 18)-Hirsutine |
| MOL008490 | Isocorynantheic acid |
| MOL000098 | Quercetin |
| MOL008635 | Coryincine |
