# Supplementary material for: Underlying Mechanism and Active Ingredients of Tianma Gouteng Acting on Cerebral Infarction as Determined via Network Pharmacology Analysis Combined With Experimental Validation
Source: Front Pharmacol. 2021 Nov 16;12:760503. doi: 10.3389/fphar.2021.760503 (PMC8635202; doi:10.3389/fphar.2021.760503)
Supplement: Supplementary file 5 [file Table3.docx]

**Supplementary Table S3** Out Pathway Enrichment

| **#** | **Pathway** | **Candidate genes with pathway annotation** | **All genes with pathway**  **annotation** | **P value** | **Q value** | **Pathway ID** |
| --- | --- | --- | --- | --- | --- | --- |
| 1 | Neuroactive ligand-receptor  interaction | 10 (32.26%) | 293 (3.44%) | 0 | 0.000008 | ko04080 |
| 2 | Serotonergic synapse | 7 (22.58%) | 125 (1.47%) | 0 | 0.000021 | ko04726 |
| 3 | Pathways in cancer | 11 (35.48%) | 550 (6.46%) | 0.000002 | 0.000108 | ko05200 |
| 4 | African trypanosomiasis | 4 (12.9%) | 42 (0.49%) | 0.000015 | 0.000618 | ko05143 |
| 5 | HIF-1 signaling pathway | 5 (16.13%) | 102 (1.2%) | 0.00003 | 0.000863 | ko04066 |
| 6 | IL-17 signaling pathway | 5 (16.13%) | 106 (1.25%) | 0.000036 | 0.000863 | ko04657 |
| 7 | Amoebiasis | 5 (16.13%) | 106 (1.25%) | 0.000036 | 0.000863 | ko05146 |
| 8 | AGE-RAGE signaling pathway  in diabetic complications | 5 (16.13%) | 114 (1.34%) | 0.000051 | 0.001071 | ko04933 |
| 9 | Cholinergic synapse | 5 (16.13%) | 121 (1.42%) | 0.000067 | 0.001267 | ko04725 |
| 10 | Thyroid hormone signaling  pathway | 5 (16.13%) | 131 (1.54%) | 0.000098 | 0.001664 | ko04919 |
| 11 | Th17 cell differentiation | 5 (16.13%) | 186 (2.18%) | 0.000505 | 0.007651 | ko04659 |
| 12 | Calcium signaling pathway | 5 (16.13%) | 189 (2.22%) | 0.000543 | 0.007651 | ko04020 |
| 13 | Human cytomegalovirus  infection | 6 (19.35%) | 307 (3.61%) | 0.000719 | 0.00873 | ko05163 |
| 14 | Endocrine and other factor-  regulated calcium reabsorption | 3 (9.68%) | 50 (0.59%) | 0.000763 | 0.00873 | ko04961 |
| 15 | Chagas disease (American  trypanosomiasis) | 4 (12.9%) | 116 (1.36%) | 0.000775 | 0.00873 | ko05142 |
| 16 | Proteoglycans in cancer | 5 (16.13%) | 210 (2.47%) | 0.000877 | 0.009259 | ko05205 |
| 17 | Regulation of lipolysis in  adipocyte | 3 (9.68%) | 56 (0.66%) | 0.001063 | 0.010211 | ko04923 |
| 18 | NF-kappa B signaling pathway | 4 (12.9%) | 127 (1.49%) | 0.001088 | 0.010211 | ko04064 |
| 19 | TNF signaling pathway | 4 (12.9%) | 130 (1.53%) | 0.001186 | 0.010552 | ko04668 |
| 20 | VEGF signaling pathway | 3 (9.68%) | 61 (0.72%) | 0.001364 | 0.011523 | ko04370 |
