# Supplementary material for: Underlying Mechanism and Active Ingredients of Tianma Gouteng Acting on Cerebral Infarction as Determined via Network Pharmacology Analysis Combined With Experimental Validation
Source: Front Pharmacol. 2021 Nov 16;12:760503. doi: 10.3389/fphar.2021.760503 (PMC8635202; doi:10.3389/fphar.2021.760503)
Supplement: Supplementary file 6 [file Table4.docx]

**Supplementary Table S4** Key targets molecule binding energies

| **Key Targets** | **Active Ingredients** | **PDB：ID** | **Binding Energy（kcal / mol）** |
| --- | --- | --- | --- |
| NFKBIA | Quercetin | 6Y1J | -5.21 |
| HIF1A | Quercetin | 3HQU | -8.7 |
| PPARG | Tetrahydroalstonine | 6FZG | -7.63 |
|  | Quercetin |  | -9.77 |
|  | Vincoside lactam_qt |  | -11.54 |
|  | Yohimbine |  | -3.55 |
